# Fossil mammals from the Gondolin Dump A ex situ hominin deposits, South Africa

**DOI:** 10.7717/peerj.5393

**Published:** 2018-08-06

**Authors:** Justin W. Adams

**Affiliations:** 1 Centre for Human Anatomy Education, Department of Anatomy and Developmental Biology, Monash University, Melbourne, VIC, Australia; 2 Centre for Anthropological Research, University of Johannesburg, Johannesburg, South Africa

**Keywords:** *Paranthropus robustus*, *Homo*, Pleistocene, Taphonomy

## Abstract

The Gondolin palaeokarstic system, located in the UNESCO Fossil Hominids of South Africa World Heritage Site, has been sporadically excavated since the 1970s. Sampling of ex situ dumpsites in 1997 recovered the only two fossil hominin specimens recovered thus far from Gondolin. While one partial mandibular molar (GA 1) remains unattributed, the complete mandibular second molar (GA 2) represents the largest *Paranthropus robustus* Broom, 1938 tooth identified to date. While subsequent excavations and research at Gondolin has clarified the geological, temporal, taphonomic, and palaeoecologic context for the in situ deposits, this paper presents the first comprehensive description of the fossil assemblage ‘associated’ with the two ex situ hominins. Analysis of 42 calcified sediment blocks and naturally decalcified sediments excavated from three cubic metres of the Dump A deposits reinforce that the dump contains a heterogeneous aggregation of materials from across the Gondolin sedimentary deposits. A total of 15,250 individual fossil specimens were processed (via sifting or acetic-acid mediated processing of calcified sediment blocks), yielding a faunal record that largely mirrors that described from either (or both) the GD 1 and GD 2 in situ assemblages but includes representatives of four novel mammal groups (Families Cercopithecidae, Felidae, Herpestidae, Giraffidae) not recorded in either in situ sample. While basic assemblage characteristics including primary taphonomic data is presented, analysis and interpretation is limited by the ex situ origin of the sample. Ultimately, these results reinforce that the substantial mining-mediated obliteration of palaeokarstic deposits at Gondolin continue to obscure a clear association between the Gondolin Dump A hominins and any of the sampled and dated in situ deposits.

## Introduction

**Figure 1:**
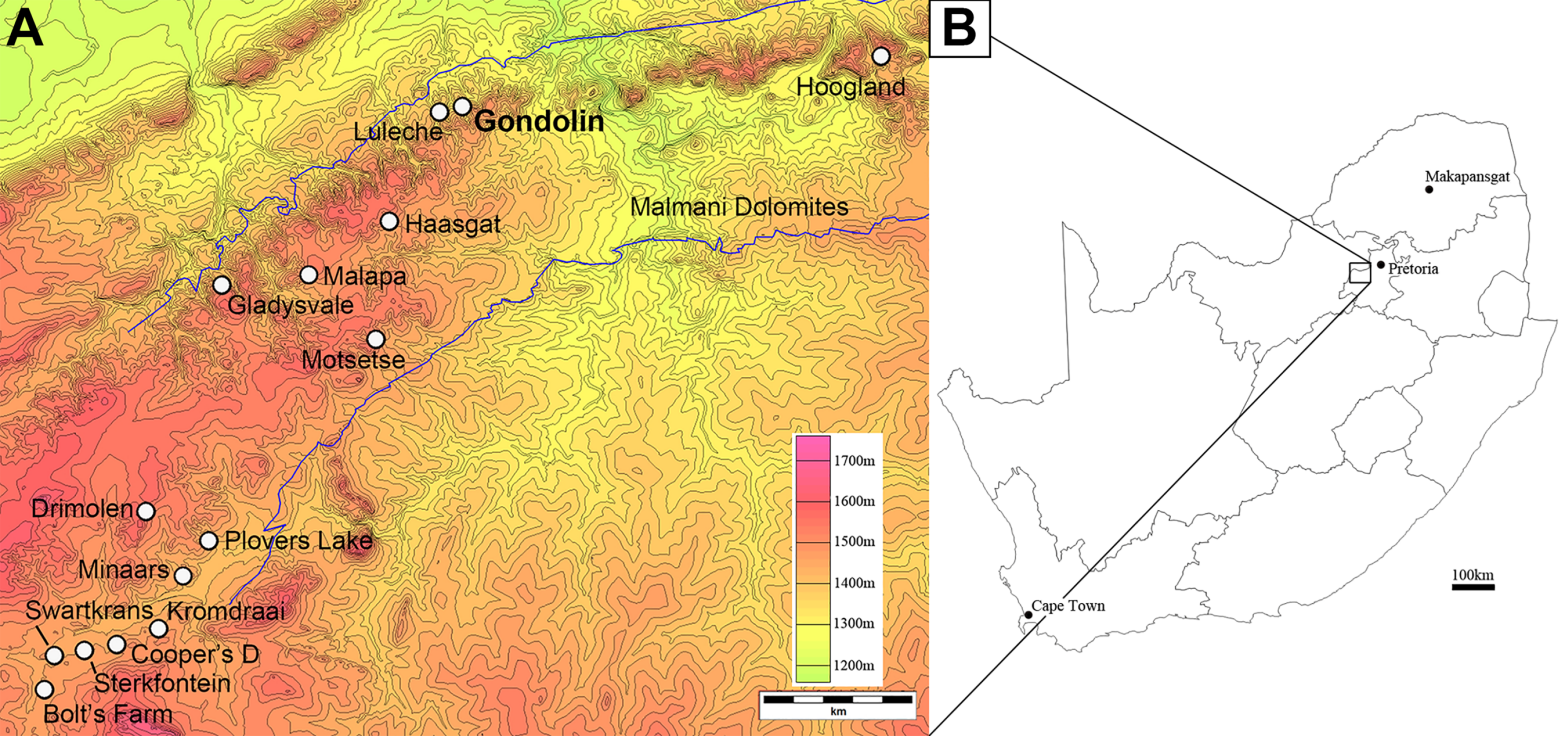
Topographic map of the UNESCO Fossil Hominids of South Africa World Heritage Site region (A) in reference to a provincial map of South Africa (B). Major fossil sites are indicated, with Gondolin designated with larger font.

Gondolin is a palaeokarstic system located 35 km north–northeast of the historically established Bloubank River valley hominin-bearing sites (e.g. Sterkfontein, Swartkrans, Kromdraai) in the UNESCO Fossil Hominids of South Africa World Heritage Site (locally termed ‘The Cradle of Humankind’) (Fig. 1). At the time Gondolin was originally discovered and excavated in the 1970s, the locality represented a geographic isolate relative to most other fossil localities in the Cradle. In the intervening decades discovery and research at a series of other fossil localities (e.g. Drimolen, Malapa, Haasgat, Luleche) have started to fill in some of the highly variable landscape across the region (Vrba, 1982; MacKenzie, 1994; Keyser et al., 2000; Dirks et al., 2010; Adams et al., 2007a, 2016; Adams, 2012b; Herries et al., 2014; Rovinsky et al., 2015).

Despite this increasing catalogue of described sites and fossil samples, Gondolin remains a unique palaeontological site in several key respects. Geologically, Gondolin lies in a heavily dissected area of the chert-rich Eccles Formation, Malmani Sub-group, of the Chuniespoort Group carbonate-Bif Marine Platform between the Magalies and Crocodile Rivers (Herries et al., 2006; Adams et al., in press). As a result of these differences in regional erosion during the Neogene and Quaternary, Gondolin lies at a slightly higher elevation (∼1,380 m) than many of the Bloubank sites (∼1,160–1,200 m) but is at the apex of a hill in a topographically relieved region (part of the Skurweberg Range) with steep rocky hillsides and narrow valley floors (Adams, 2010; Adams et al., in press). Ecologically, this topography influences the modern (and presumably prehistoric) density and distribution of vegetation, yielding a rough gradient of open habitats (grassland, bushland) to open-to-closed woodland vegetation when traversing from hill summits to valley floors. While currently the valley floors only serve as temporary catchments for seasonal runoff that supports denser vegetation, prior to the transition to current climate and rainfall patterns they may have retained more extensive surface/subsurface water as part of the drainage basin of the nearby Crocodile River during the early Pleistocene given the abundance of closed habitat and browse-adapted taxa at Gondolin and Haasgat (Adams, 2012b; Adams et al., in press). And finally, in terms of palaeontology, this difference in landscape has likely existed since at least the early Pleistocene; the in situ Gondolin deposits, which were deposited just before (GD 2) and after (GD 1) the Olduvai SubChron at 1.78 million years ago (Ma) are also unique relative to other penecontemporaneous site deposits in both taxon occurrence and abundance (Menter et al., 1999; Adams & Conroy, 2005; Herries et al., 2006; Adams et al., 2007b; Adams, 2010). This includes the dominance of infrequently encountered extinct faunal lineages (e.g. reedbuck, klipspringer) and the absence of identifiable hominin or other primate remains across the substantial samples from the GD 1 (number of individual specimens [NISP]: 4,843) and GD 2 in situ deposits (NISP: 95,549) (Adams & Conroy, 2005; Adams et al., 2007b; Adams, 2010).

**Figure 2:**
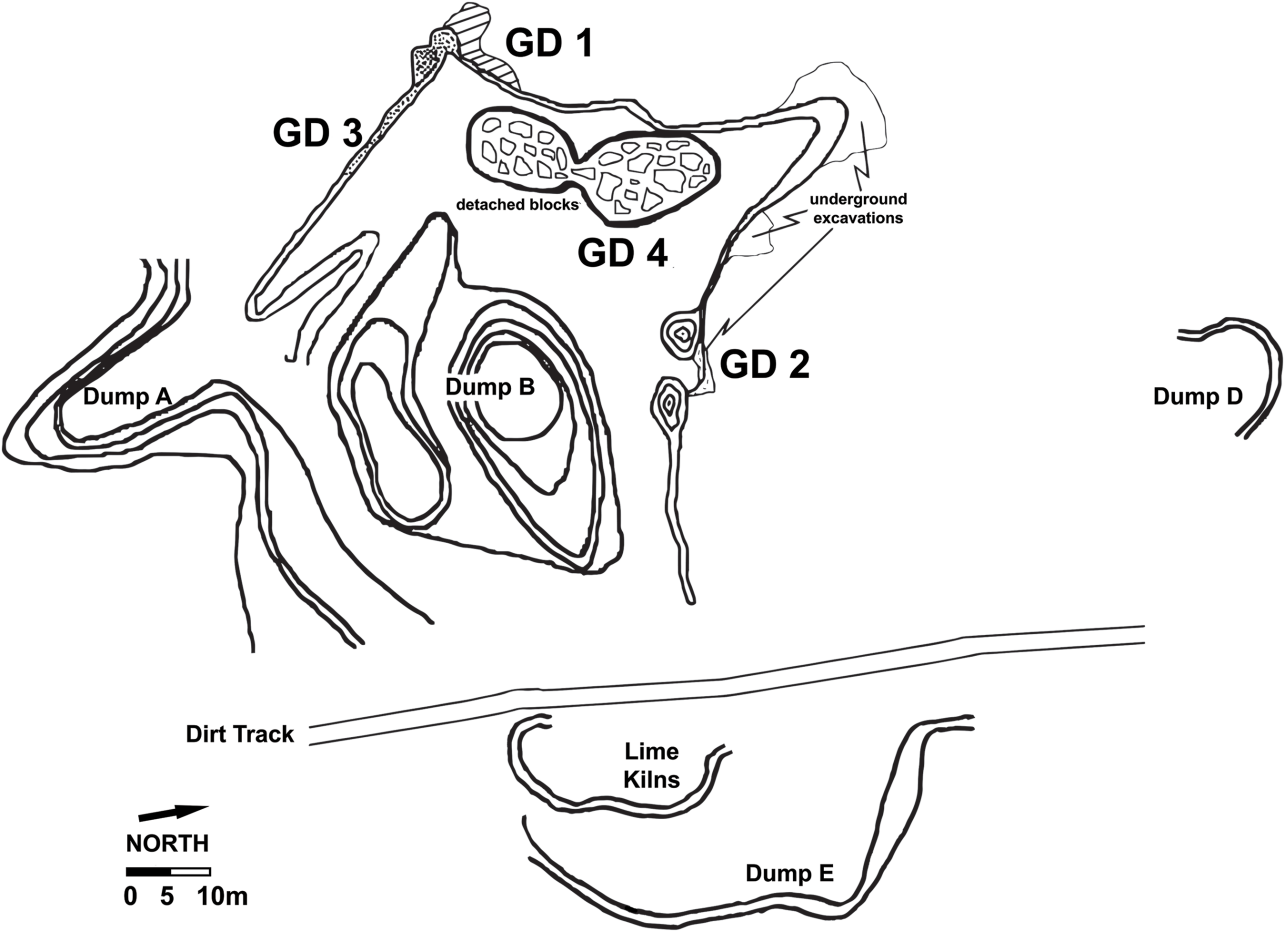
Site plan of the modern Gondolin cave, including the location of the in situ sediment remnants (GD 1–GD 4) and the location of Dump A deposits sampled to produce the GD A assemblage. Adapted from Menter et al. (1999) with revised positioning of elements to correct erroneous placement of site features and cardinal directions.

**Figure 3:**
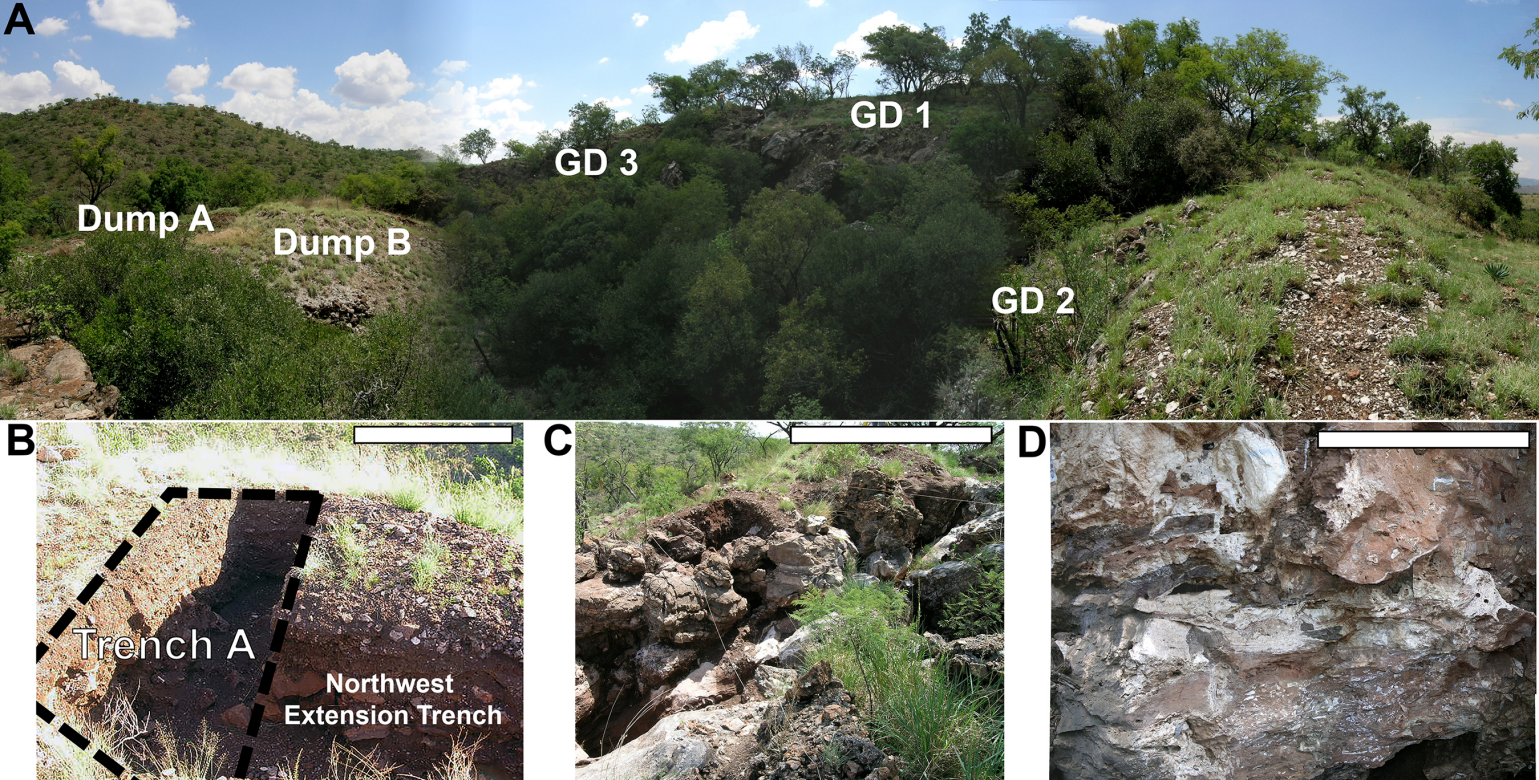
Photographs of the modern Gondolin site. (A) Panorama of Gondolin, including the location of the in situ sediment remnants GD 1–GD 3, Dump B, and Dump A deposits. (B) The excavated Trench A which yielded the GD A assemblage and the Northwest Extension Trench excavated in 2003. (C) Makondos of the GD 1 deposits with exposed grey calcified sediments. (D) The GD 2 in situ hanging remnant with exposed fossil bed within red calcified sediment matrix. Scale bars equal one m; no scale provided for (A) given distortion of the panoramic image. Photo credits: Justin W. Adams.

Although neither of the sampled GD 1 or GD 2 in situ deposits have yielded hominin or primate remains, Gondolin is nonetheless a hominin-bearing fossil site. A phase of survey and excavation into extensive ex situ dumpsites at Gondolin in 1997 included excavation of two adjacent one m units (Trench A) into one of the larger dumpsites (Dump A) (Figs. 2 and 3; Menter et al., 1999). The ex situ dumpsite included representative samples from most, if not all, of the stratigraphic units that were originally present at the site prior to commercial mining activity (Menter et al., 1999; Adams et al., 2007b). From the test trench sample Menter et al. (1999) described the only two hominin specimens currently known from the site. The first, GDA-1 (as described *per* Menter et al.; formally catalogued as GA 1 *per* Adams, 2006), is a worn and fractured left mandibular first or second molar potentially attributed to *Homo*. The second, GDA-2 (as described *per* Menter et al.; formally catalogued as GA 2 *per* Adams, 2006) is a complete left mandibular second molar recently reanalysed and attributed to *Paranthropus robustus* (Grine et al., 2012). Although substantial collections of both sterile and fossiliferous calcified sediment blocks were removed from Trench A (as well as loose fossils from sediments screened during excavation), the materials were only sporadically processed and no systematic attempt to process these blocks (or analyse the fossil specimens from these ex situ materials) was undertaken at that time.

A comprehensive phase of research activity at the Gondolin locality and on the fossil assemblages from site deposits has clarified the primary geological context of the palaeokarstic system (Menter et al., 1999; Herries, 2003; Herries et al., 2006; Adams et al., 2007b; Herries & Adams, 2013), depositional history and age (Herries et al., 2006; Adams et al., 2007b; Herries & Adams, 2013), the anatomy and attribution of the Gondolin Dump A (GD A) hominins (Menter et al., 1999; Kuykendall, 2012; Grine et al., 2012), site faunas (Watson, 1993b; Adams & Conroy, 2005; Adams, 2006, 2012a; Adams et al., 2007b), assemblage taphonomy (Herries et al., 2006; Adams et al., 2007b; Adams, 2010) and regional palaeoecology (Adams et al., in press). However, absent from this body of publication is a primary description and interpretation of the Trench A fossil faunas processed from calcified sediment blocks and sifted sediments excavated by Menter et al. (1999). The primary description and analysis of this faunal assemblage ‘associated’ with these two hominin specimens, named the GD A assemblage, is provided here in order to offer a more comprehensive faunal context for the ex situ Gondolin hominin specimens, along with a discussion of the fossil sample characteristics relative to the in situ GD 1 and GD 2 deposits.

## Materials and Methods

Before describing the GD A assemblage it is critical to note a few aspects of site and excavation history to establish which deposits most likely contributed to the formation of Dump A ex situ deposits that were originally sampled by Menter et al. (1999). As is the case for many of the currently described South African Neogene and Quaternary cave sites, the Gondolin palaeocave was mined for lime deposits at some point during the 20th century (Figs. 2 and 3). This mining activity was notably intense at the site, as evinced by the minimal residual in situ calcified sediment hanging remnants on the northern (GD 2), western (GD 1), and southern (GD 3) perimeter of the modern system, and the massive calcified sediment dumpsites both within and around the margins of the locality (Menter et al., 1999; Adams, 2006, 2010; Herries et al., 2006; Adams et al., 2007b). The exposed calcified sediment remnants near the GD 2 datum point established by Menter et al. (1999) were first noted by K. MacKenzie during a horseback riding trip in 1977, who reported the site to Elisabeth Vrba at the (then) Transvaal Museum (Figs. 2 and 3; Vrba, 1982; MacKenzie, 1994). This led to the excavations in 1979 by E.S. Vrba and D. Panagos, who removed approximately two cubic metres of highly fossiliferous in situ red siltstone from the northern (GD 2) hanging remnant (Vrba, 1982; Watson, 1993b; Figs. 2 and 3D). The calcified materials were removed in reference to a vertical grid, marked as 43 separate blocks, and processed using acetic acid to maximize specimen recovery. The resulting faunal assemblage, termed the GD 2 assemblage, has been thoroughly described through a series of publications (Watson, 1993b; Adams & Conroy, 2005; Adams, 2006, 2010; Herries et al., 2006) and has been interpreted as representing skeletal materials largely accumulated by a leopard or leopard-like felid, directly interred in the GD 2 region of the cave system, and likely over a short time period (Adams, 2010).

The only other fossiliferous in situ deposit noted by Menter et al. (1999) was along the western rim of the mined cave system near the GD 1 datum point, where heavily weathered calcified sediment outcrops and makondos have served as a catchment for naturally decalcified sediments (Figs. 2 and 3B; Adams et al., 2007b; to date, no fossil materials have been noted within the GD 3 in situ remnant). The GD 1 exposed sediments were excavated in 2003 and found to represent a fundamentally different in situ sample of the Gondolin deposits to the GD 2 hanging remnant in terms of both deposit formation and assemblage taphonomy (Herries et al., 2006; Adams et al., 2007b; Adams, 2010). The GD 1 deposits represent a series of inter-stratified speleothem, in-washed sediments and talus deposits that record multiple phases of sediment infilling, undercutting, and reworking of material (Adams et al., 2007b); contrasting the apparent single sediment infilling and chronologically constrained formation of the GD 2 deposits (Herries et al., 2006). The thick flowstones of the GD 1 deposits acted as false floors under which sediment and calcified sediments were eroded out and redeposited at much later periods, resulting in an inverted stratigraphic sequence (Adams et al., 2007b). Skeletal materials and a significant load of weathered chert-lag debris were introduced into the GD 1 area largely through in-washing of skeletal materials via a vertical aven entrance that was separate from the lateral, open entrance near the GD 2 depositional area that allowed for direct felid-mediated interment (Adams, 2010). This depositional process occurred at GD 1 during a period where the magnetic field was changing from normal to reversed polarity at the end of the Olduvai SubChron at ∼1.78 Ma; again contrasting with the slightly older GD 2 deposits that formed in a period of normal polarity within the Olduvai SubChron (∼1.95–1.78 Ma; Adams et al., 2007b). However, the occurrence of the same ∼1.78 Ma reversal in speleothem capping GD 2 perhaps suggests it is closer to 1.78 than 1.95 Ma (Herries et al., 2006).

Because of the extensive mining that occurred into the Gondolin palaeocave system there is more volume of ex situ than in situ calcified sediment that can be excavated and sampled (Figs. 2 and 3; Menter et al., 1999). This modern condition of the Gondolin system does explain why ex situ dumpsites were originally targeted by Menter et al. (1999); however, it equally highlights the limited ability to associate dumpsite sediments to the few exposed cubic meters of in situ sediment remnants given the loss of most of the dolomitic and sedimentary deposits of the original cave system (see Herries & Adams, 2013). There were two main ‘types’ of calcified cave sediment extracted from a two m by one m trench (Trench A) excavated to a depth of around two m from the ex situ Dump A deposits (Fig. 2): a grey ‘Conglomerate Unit’ with minimal fossil remains and a matrix characterized by large clasts, well-rounded pebbles, and CaCO_3_ crystals; and a red ‘Finer Clastic Unit’ with a higher frequency of fossil specimens and a matrix of fine, reddish silt with only small and sporadic clasts (Menter et al., 1999). Both hominin teeth (GA 1 and 2) were processed from small blocks fitting the ‘Finer Clastic Unit’ profile (Menter et al., 1999). As tempting as it is link these specimens to the GD 2 depositional region simply because it is the sole fossil-bearing red siltstone remnant (or the grey Conglomerate Unit blocks to GD 1; Adams et al., 2007b), the mining and erosional obliteration of most of the cave walls, roof, talus cone and/or other sedimentary formations means that entire unique depositional phases could be represented in the dumpsites that are unrelated to the few in situ remnants (Herries & Adams, 2013).

Approximately 50 calcified sediment blocks from the 1997 excavations, as well as loose fossil specimens and clasts that were sieved using one mm mesh screens from Trench A, were initially moved from the site and stored under the supervision of Dr. K.L. Kuykendall at the School of Anatomical Sciences, University of the Witwatersrand Medical School, Johannesburg. Over the intervening years several of the calcified sediment blocks from the excavation had been processed using acetic acid of varying concentrations (8–15%) and mechanical (air scribe) preparation, yielding a small fossil collection termed the GD A assemblage (K. Kuykendall, 2003, personal communication). Starting in 2003, I began a concentrated phase of processing and the first cataloguing of the Gondolin Trench A materials. In order to standardize the processing method an experiment was conducted in June 2003 at the School of Anatomical Sciences Fossil Preparation Lab to determine an appropriate acetic acid concentration for use on the Trench A calcified sediment blocks. A total of six small (∼20 cm diameter), relatively sterile blocks were selected for the experiment, representing three blocks each of the grey ‘Conglomerate Unit’ sediment type and the red ‘Finer Clastic Unit’ type. Each block was placed into separate plastic containers and entirely submerged in an acetic acid solution of 6%, 8% or 10%. Each container was placed inside a fume hood and allowed to react with the acid for 48 h, at which time a tri-calcium phosphate (Ca_3_(PO_4_)_2_) buffer was added. After a total of 72 h submerged each container was drained of the acetic acid solution, washed, and submerged in circulating fresh water for 9 days (three times longer than the acid cycle). When drained of the acid solution, any sediment, clasts and/or fossil specimens that were dislodged by the acid bath process were sieved using fine (one mm) mesh from the buckets, washed, and later sorted for fossil remains under a binocular dissecting microscope (#176550; Vickers Instruments, York, UK). Any macromammalian fossil specimens were removed for analysis, while microfaunal remains were bagged and stored. After the calcified sediment blocks had been allowed to soak in water for 9 days, they were removed and placed on wire racks to air dry for 5 days.

Each block and associated sediments were visually inspected to assess the effects that the varying concentrations of acetic acid had on the two types of calcified sediments. There were no perceptible differences between the different concentrations and their effect on the red siltstone ‘Finer Clastic Unit’ blocks in terms of visible signs of acid etching on the blocks (and any fossil specimens), the amount of sediment produced, or the number of specimens dislodged. Among the grey ‘Conglomerate Unit’ block specimens, however, there was a visible increase in the reduction of block mass, as well as an increase in the recovery of specimens from sediments with increasing acid concentration. At the same time, there was an increase in visible acid damage to fossil specimens from the 8% to the 10% concentrations. I therefore determined that the best combination of processing rate and conservation of the GD A fossils was to process all GD A blocks using an 8% acetic acid solution.

A total of 42 blocks (20 red siltstone and 22 grey breccia) excavated in 1997 by Menter et al. (1999), as well as decalcified materials sifted from the Trench A excavation squares, were processed to develop the GD A assemblage described here. I would also note that the onset of my fieldwork at Gondolin in 2003 included the excavation of a two m by one m trench perpendicular to Trench A (the ‘Northwest Extension’ Trench; Fig. 3B) to recover additional calcified sediment blocks and decalcified sediments. This 2003 sample of sediment blocks is currently reposited with the Ditsong National Museum of Natural History pending acetic acid processing, and therefore is not further described here. All macromammalian fossil specimens (e.g. all Orders other than Chiroptera, Macroscelidea, Insectivora, and Rodentia (exc. Family Hystridae)) were identified, sorted and coded for taxonomic, demographic, and taphonomic variables following the methods fully described in Adams (2006, 2010) and Adams & Rovinsky (in press). Although I list the small Class Aves component in Table 1 and briefly describe the specimens below, no attempt was made to provide taxonomic identifications of this material; it is being presented simply to alert the palaeontological community to their presence within the sample. Taxonomic identifications of all craniodental and postcranial specimens were made in reference to modern skeletal collections at the Ditsong National Museum of Natural History, previously described fossil specimens at the Ditsong Museum (Swartkrans, Kromdraai, Cooper’s, Sterkfontein Type Site, Gondolin GD 2, and Hoogland) and the Evolutionary Studies Institute (Makapansgat), and published diagnostic criteria (summarized in Adams, 2006). Bovid specimens that could only be confidently attributed to Family were sorted into Size Classes after Brain (1976). All measurements of specimens reported here were taken using Mitutoyo 150 mm calipers with a direct digital input, including relevant dental metrics (mesiodistal (MD) and buccolingual (BL); taken at the level of occlusion unless otherwise noted).

**Table 1:** Gondolin GD A faunal assemblage.

Taxon	NISP	MNI
Class Mammalia		
Order Primates		
Family Cercopithecidae		
Cercopithecidae indet.	1	1
Family Hominidae		
Tribe Hominini		
*Paranthropus robustus*	1	1
cf. *Homo* sp.	1	1
Primates gen. et sp. indet.	1	1
Order Carnivora		
Family Felidae		
Felidae gen. et sp. indet.	2	1
Family Hyaenidae		
Hyaenidae gen. et sp. indet.	1	1
Family Herpestidae		
Herpestidae gen. et sp. indet.	3	1
Carnivora gen. et sp. indet.	3	–
Order Cetartiodactyla		
Family Bovidae		
Tribe Alcelaphini		
Alcelaphini indet. (medium)	75	10
Alcelaphini indet. (large)	6	2
Tribe Antilopini		
*Antidorcas* sp.	5	3
Tribe Hippotragini		
*Hippotragus equinus*	4	1
Tribe Oreotragini		
*Oreotragus* sp.	10	3
Tribe Reduncini		
*Redunca* sp.	16	4
Tribe Tragelaphini		
*Tragelaphus oryx*	3	1
*Tragelaphus strepsiceros*	1	1
Tragelaphini indet.	10	3
Bovidae gen. et sp. indet.		
Class I	14	–
Class II	87	–
Class III	14	–
Indet. Class	1,259	–
Family Giraffidae		
*Giraffa* sp.	1	1
Family Suidae		
Suidae gen. et sp. indet.	4	1
Order Perissiodactyla		
Family Equidae		
*Equus* cf. *capensis*	3	3
*Equus* sp.	11	–
Order Hyracoidea		
Family Procaviidae		
*Procavia* sp.	4	1
Order Lagomorpha		
Family Leporidae		
Leporidae gen. et sp. indet.	2	1
Order Rodentia		
Family Hystricidae		
*Hystrix africaeaustralis*	1	1
*Hystrix makapanensis*	4	2
Hystricidae gen. et sp. indet.	4	
Mammal indet.	735 (12,958)	–
Class Aves		
Aves gen. et sp. indet.	4	3
Total	2,292 (15,246)	48

**Notes:**

Table includes data from Menter et al. (1999), Adams (2012b) and Grine et al. (2012). Numbers in parentheses represent those specimens coded as taxonomically unidentifiable, primarily long bone diaphysis macromammalian fragments as a subset and their contribution to the assemblage total.

A single indeterminate mammal diaphysis specimen from the decalcified sediments (GA 763) was imaged using a scanning electron microscope (SEM) to evaluate possible hominin-induced stone tool cutmarks noted during collection prior to 2003. Prior research has indicated that SEM evaluation can assist in differentiating marks produced by hominins (which exhibit a low breadth:depth ratio, V-shaped cross-sections and microstriations in internal surfaces) from those produced by carnivores (Potts & Shipman, 1981; Shipman, 1981; Shipman & Rose, 1984). A negative peel of the GA 763 specimen was taken using a liquid silicone moulding compound, from which a positive cast was produced by Mr. M. Veith of the Department of Biology at Washington University in St. Louis (USA) using Poly/Bed 812—BDMA epoxy embedding resin (Polysciences, Inc. Warrington, PA, USA). The mould was filled with liquid epoxy resin mix, placed into a vacuum oven at 70 °C, and allowed to polymerize for 26 h. The polymerized positive was attached to an aluminium specimen mount using a hot glue gun, and coated with gold in a Polaron E-5000 SEM Coating Unit. A series of images were taken using a Hitachi S-450 SEM at 20 KV accelerating voltage. The positive was oriented and viewed at several different powers and angles to examine the internal morphology of marks, with the resulting images recorded on Polaroid 55 Positive/Negative film.

Ultimately, most of the fossil specimens in the GD 1 assemblage could not be attributed to species or genus, and the assemblage preserves a range of mammals lineages that are also commonly represented in African Quaternary fossil and recent archaeological assemblages. As such, comprehensive synonym citation and summaries of the geographic and temporal range of genera and species are beyond the scope of this publication. Additional synonym information and data on the occurrence and ranges of mammals discussed here can be found in Wilson & Reeder (2005), Werdelin & Sanders (2010) and Kingdon et al. (2013). As both hominin specimens have been previously described in Menter et al. (1999) and Grine et al. (2012), I have elected not to duplicate these works and include formal systematic palaeontology for these two specimens. Equally, the GD A *Hystrix makapanensis* Greenwood, 1958 were integrated in a prior publication on the hystricid remains from the Gondolin site and are not described again here (Adams, 2012a).

## Results

The catalogued GD A assemblage from the calcified sediment blocks and decalcified sediments consists of 15,246 macromammalian and four avian fossil specimens. Only 2,292 (15.03%) of the GD A assemblage materials are craniodental or postcranial remains identifiable to taxon and/or element (Table 1; Tables S1 and S2), while the remaining 12,958 specimens (84.97%) are unidentifiable (primarily long bone diaphysis) macromammalian fossil fragments (Table S2). Decalcified sediments sifted from the trench produced 8,245 (54.07%) of the total specimens from the assemblage, of which 70 are identifiable craniodental or postcranial specimens, 1,055 are indeterminate bovid enamel fragments, 46 are indeterminate mammal enamel fragments, and the remaining 7,074 specimens are indeterminate element mammal fossil fragments. The other 7,005 specimens from the total assemblage were processed from the ex situ calcified sediment blocks removed during excavation. The processed red calcified sediment blocks (*n* = 20) yielded 2,840 specimens, of which 71 are identifiable craniodental or postcranial remains, 40 specimens are indeterminate bovid enamel fragments, 74 are indeterminate mammal enamel fragments, and 2,661 specimens are unidentifiable element mammal fragments. The grey calcified sediment blocks (*n* = 22) yielded 2,591 specimens, of which 47 are identifiable craniodental or postcranial remains, 103 are indeterminate bovid enamel fragments, 433 are indeterminate mammal enamel fragments, and 2,008 specimens are unidentifiable element mammal fragments. The remaining 1,574 processed specimens were processed from sediment blocks prior to 2003 and lacked any record of the calcified sediment type the specimens were derived from.

Of the 2,292 identifiable remains, 1,557 (67.93%) could be classified to the Family or more specific level of identification, while 85 were indeterminate postcranial elements or element portions and 650 were unidentifiable enamel fragments (of which nine were possibly bovid and seven were fragments derived either from a bovid or equid) (Table 1; Table S1). The majority of the classifiable remains (NISP = 1,259) were indeterminate bovid enamel fragments (Table S2).

## Systematic Palaeontology

Order PRIMATES Linnaeus, 1758
Family CERCOPITHECIDAE Gray, 1821
Cercopithecidae gen. et sp. indet.

Referred Specimen. GA 55, left proximal ulna.

**Figure 4:**
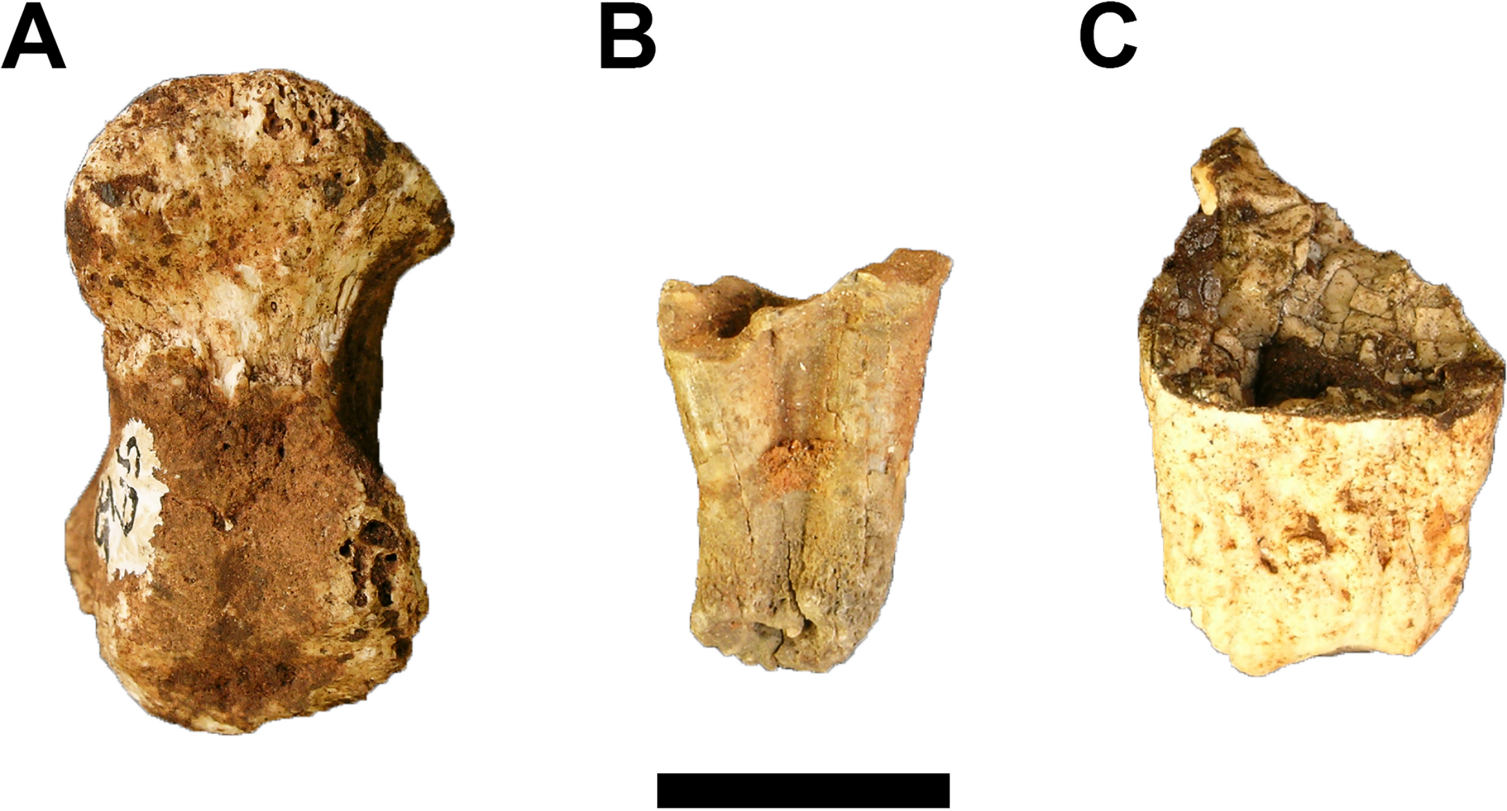
Fossil Primates from Gondolin GD A. (A) Cercopithecidae gen. et sp. indet. GA 55 left proximal ulna. (B) ?Primates gen. et sp. indet. GA 165 probable maxillary lingual molar root. (C) ?Primates gen. et sp. indet. GA 198 probable mandibular molar root. Scale bar equals one cm. Photo credits: Justin W. Adams.

The GA 55 left proximal ulna was removed from the Trench A decalcified sediments and exhibits substantial cortical exfoliation (Fig. 4A). Despite the cortical flaking, the element does preserve most of the proximal trochlear notch articular surface and full anterior and proximal extent of the olecranon process. The specimen is directly comparable in articular surface morphology and olecranon shape with larger modern *Papio* Statius Müller, 1,773 proximal ulnae but no linear metrics could be established.

?Primates gen. et. sp. indet.

Referred Specimen. GA 165, probable maxillary lingual molar root; GA 198, probable mandibular molar root.

Two isolated fully formed and closed tooth roots without crowns are tentatively classified here as indeterminate primate remains. The first, GA 165, is a probable maxillary lingual molar root with part of the central pulp chamber but lacking any enamel near the enamel-dentine junction (Fig. 4B). The second specimen, GA 198, is a probable lower molar root that is taurodont and lacks any enamel (Fig. 4C). While neither specimen is diagnostic, they both conform to the molar root morphology expressed by primates to the exclusion of the molar roots in similarly sized mammal orders (e.g. Carnivora, Cetartiodactyla).

Order CARNIVORA Bowdich, 1821
Family FELIDAE Fischer von Waldheim, 1817
Felidae gen. et sp. indet.

Referred Specimens. GA 318, right partial maxillary fourth premolar; GA 319, right partial maxillary third premolar.

**Figure 5:**
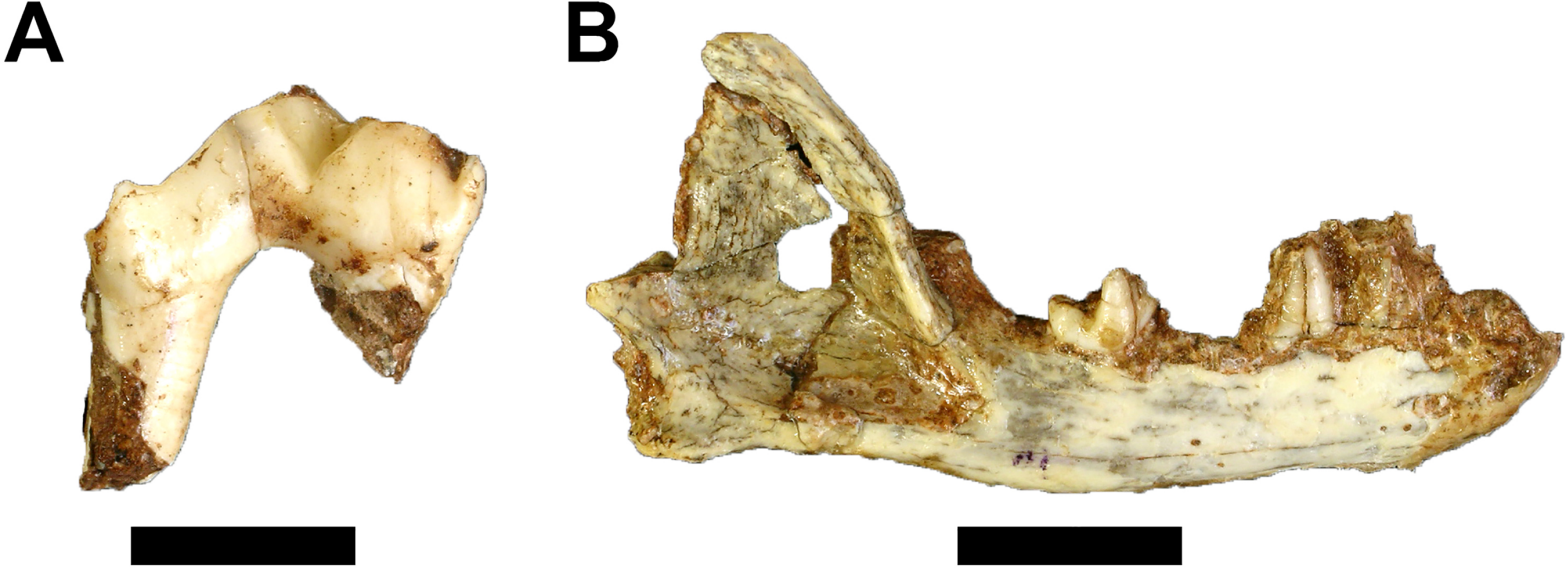
Fossil Carnivora from Gondolin GD A. (A) Felidae gen. et sp. indet. GA 319, right partial maxillary third premolar; (B) Herpestidae gen. et sp. indet. GA 1484, right mandible with partial second and third premolars and first molar. Scale bars equal one cm. Photo credits: Justin W. Adams.

The only two partial felid maxillary premolars present in the GD A sample may be associated given their size and preserved morphology; however, as they were processed during early phases of sampling and processing of the Trench A materials we lack data on the blocks they were derived from. Both specimens are too incomplete to establish standard dental metrics but are visibly derived from a felid that most closely overlaps in tooth size with extant caracal (*Caracal caracal* Schreber, 1776) and is distinctly smaller than extant and fossil leopard (*Panthera pardus* Linnaeus, 1758) and larger than extant serval (*Leptailurus serval* Schreber, 1776) comparative specimens (Fig. 5A).

Family HERPESTIDAE Bonaparte, 1845
Herpestidae gen. et sp. indet.

Referred Specimens. GA 1482, right mandibular fourth premolar; GA 1483, left partial mandible with partial fourth premolar and first molar; GA 1484, right mandible with partial second and third premolars and first molar.

Three indeterminate herpestid craniodental specimens were recovered from a single, small red calcified sediment block (Block 03-1) and are therefore considered representing a single individual (Fig. 5B). Although there is mechanical preparation damage to the tooth crowns, as a group, the GD A herpestid mandibular remains compare favourably to the SK 310 *Crossarchus transvaalensis* Broom, 1937 right mandible. The GA 1482 p4 (MD: 5.87 mm, BL: 3.5 mm) is comparable in size to the SK 310 p4 (MD: 5.88 mm, BL: 3.04 mm). The GA 1482 p4 crown differs from SK 310 in the more rounded cingulum on the distal and lingual border relative to the sharper cingulum morphology present on the SK 310 specimen. In this respect, GA 1482 more closely reflects the cingulum development present in specimens of *Herpestes* (e.g. *Herpestes ichneumon* Linnaeus, 1758); although representing a smaller taxon than *Herpestes ichneumon*. Given the preservation of the recovered GD A herpestid materials, the specimens can only be confidently assigned to the Family.

Family HYAENIDAE Gray, 1821
Hyaenidae gen. et sp. indet.

Referred Specimen. GA 2015, terminal phalanx.

A single complete terminal phalanx is comparable in both size and morphology to third phalanges of extant hyaenid comparative specimens (*Crocuta* Kaup, 1828 and *Hyaena* Brisson, 1762), and is morphologically dissimilar to those of specialized ungal base, crest, and process morphology of modern or fossil felids or canids.

Carnivora gen. et sp. indet.

Referred Specimens. GA 1816, indeterminate position thoracic vertebra left transverse process and prezygapophysis; GA 2121, indeterminate side and position partial premolar crown; GA 2129, indeterminate side and position canine crown.

Three specimens are diagnosable as carnivores but could not be allocated confidently to even the level of Family. The GA 1816 partial vertebra that is analogous in size and morphology to thoracic vertebrae of a black-backed jackal (*Canis mesomelas* Schreber, 1775). The GA 2121 tooth fragment that exhibits an overall crown shape and enamel thickness consistent with a felid premolar. Finally, the GA 2129 canine crown is minimally from a medium-sized carnivore (like *Canis mesomelas*), but lacks distinguishing morphology (in part because of small rodent gnawing that obliterated part of the specimen).

Order CETARTIODACTYLA Montgelard et al., 1997
Family BOVIDAE Gray, 1821
Tribe ALCELAPHINI de Rochebrune, 1883
Alcelaphini gen. et sp. indet.

Referred Specimens. See Table S1.

A total of 81 craniodental specimens were identifiable as alcelaphins, but both specimen preservation and the dental morphologic and metric overlap across species and genera within the tribe prevented more specific identification. Two broad groupings within the GD A alcelaphin sample can be distinguished. The first group (NISP: 75) were remains derived from smaller to medium sized (e.g. class II–III) alcelaphins, while the second group of the remaining six specimens were derived from larger, class III alcelaphin taxa.

**Figure 6:**
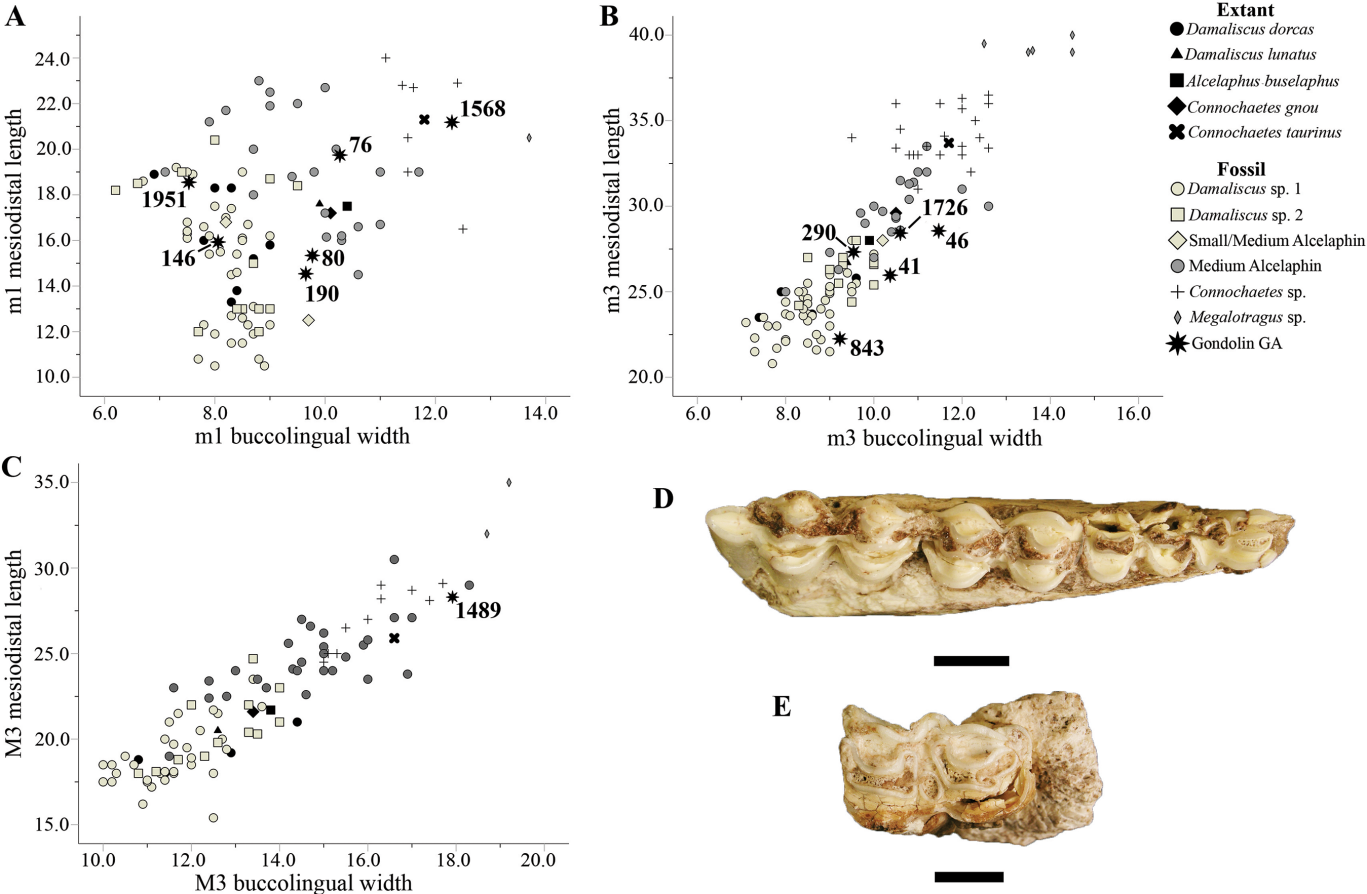
Fossil Alcelaphini from Gondolin GD A. (A) Bivariate plot of GD A, fossil and extant alcelaphin specimen mandibular first molar mesiodistal length (in millimetre) on buccolingual width (in millimetre). (B) Bivariate plot of GD A, fossil and extant alcelaphin specimen mandibular third molar mesiodistal length (in millimetre) on buccolingual width (in millimetre). (C) Bivariate plot of GD A, fossil and extant alcelaphin specimen maxillary third molar mesiodistal length (in millimetre) on buccolingual width (in millimetre). (D) Alcelaphini gen. et sp. indet. GA 41 right mandible preserving the fourth premolar to the third molar. (E) Alcelaphini gen. et sp. indet. GA 1489 left maxillary third molar. Scale bars equal one cm. All extant alcelaphin species values are mean tooth measurements, and along with fossil comparative measurements, are derived from Vrba (1976) and presented in Table S3. Photo credits: Justin W. Adams.

Of the 75 class II–III alcelaphins, eight specimens (GA 32, 38, 41, 146, 230, 843, 1951, and 2029) were derived from a smaller alcelaphin taxon similar in size to *Damaliscus* Sclater & Thomas, 1894 or *Parmaluris* Hopwood, 1934. A scatterplot of alcelaphin m1 linear metrics (MD length on BL width; Fig. 5A; Table S3) with the GA 146 and GA 1951 right lower molars (m1 or m2) demonstrate their position near fossil *Damaliscus* m1 specimens from the South African fossil sites. Similarly, a scatterplot of alcelaphin m3 dental dimensions with the GA 41 and GA 843 m3 specimens (Fig. 6B; Table S3) show their position within the upper bounds of the distribution of *Damaliscus* m3 specimens. One of the more complete specimens, the GA 41 right mandible preserves the p4 through m3 and exhibits the same strong MD pinching of the buccal cusps shared with the G 4939 *Damaliscus* sp. (*niro*?) from the GD 2 deposits (Adams & Conroy, 2005; Adams, 2006) and the *Damaliscus* sp. 2 (*niro*?) specimens from Swartkrans (Vrba, 1976) (Fig. 6D). All of the (minimally) three individuals in this smaller alcelaphin group were fully mature adult animals based on the occlusal wear stages noted on the p4 and m3 specimens.

The remaining 67 class II–III alcelaphin craniodental materials were similar in size to extant *Alcelaphus buselaphus* Pallas, 1766 comparative specimens, although not necessarily in morphology or suggestive of generic attribution. A scatterplot of the MD length on BL width of three of the m1 or m2 specimens from this grouping (GA 76, 80, and 190) show their position relative to the small and medium alcelaphin specimens from the South African fossil assemblages (with both the GA 80 and 190 specimens are plotted near the area of overlap with species of *Damaliscus*) (Fig. 6A; Table S3). This same broad pattern can be seen in the scatterplot of GD A m3 specimen dental dimensions (GA 46, 290, and 1726) (Fig. 6B; Table S3). Five of the minimally seven indeterminate medium sized alcelaphins were fully adult individuals, based on the occlusal wear stage of the m3 specimens; while minimally two immature individuals (under 3 years of age) are indicated by the recovery of the deciduous p4 remains based on dental eruptions schedules for *Connochaetes taurinus* Burchell, 1824 (Van Zyl, 1972).

Finally, the six larger class III alcelaphin specimens are broadly similar in size to craniodental remains of extant *Connochaetes* Lichtenstein, 1814. Only two of these specimens, the GA 1568 left m1 or m2 and the GA 1489 M3 (Fig. 6E) were complete enough to metrically compare to other alcelaphin molar specimens and both fall within the range of modern and fossil *Connochaetes* and/or the largest of the indeterminate medium alcelaphins (Figs. 5A and 6B; Table S3). Of the minimally two large bodied alcelaphin individuals represented in the deposits, one is fully mature (based on occlusal wear to the GA 1489 M3 specimen), while the other was an individual just prior to full maturity (under 3 years of age based on the unworn GA 337 P4) if a *Connochaetes taurinus* eruption timing schedule is applied (Van Zyl, 1972).

Tribe ANTILOPINI Gray, 1821
Genus ANTIDORCAS Sundevall, 1847
Type species *Antidorcas marsupialis* Zimmerman, 1780
*Antidorcas* sp.

Referred Specimens. GA 36, right maxillary molar (M2?); GA 242, left maxillary M3; GA 248, left maxillary molar (M3?); GA 251, right mandibular molar (m2?); GA 253, right maxillary M3 (not antimeric to GA 242).

**Figure 7:**
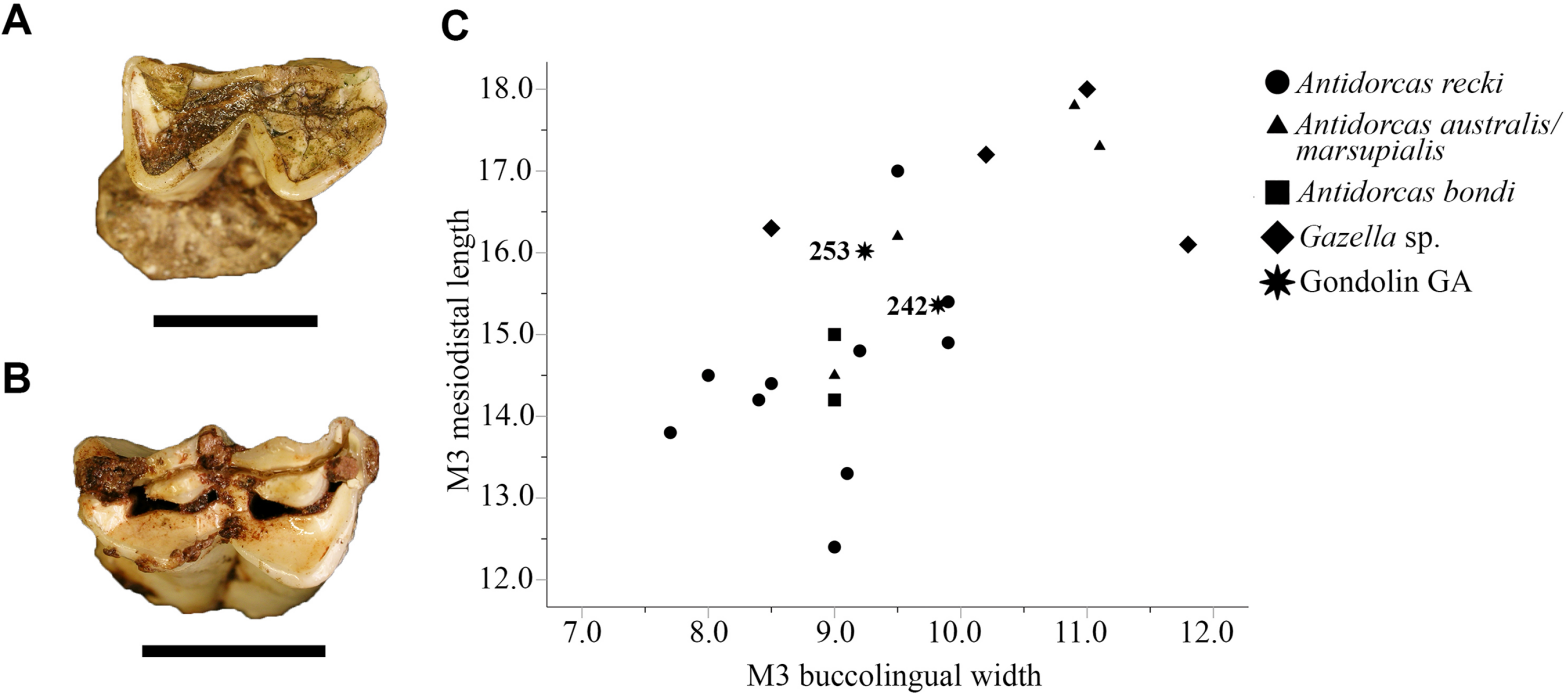
Fossil Antilopini from Gondolin GD A. (A) *Antidorcas* sp. GA 251 right mandibular molar. (B) *Antidorcas* sp. GA 253, right maxillary third molar. (C) Bivariate plot of GD A, fossil and extant antilopin specimen maxillary third molar mesiodistal length (in millimetre) on buccolingual width (in millimetre). Scale bars equal one cm. All comparative measurements from Vrba (1976) and Harris (1991) and reported in Table S4. Photo credits: Justin W. Adams.

A total of five isolated antilopin dental specimens from the GD A deposits can be attributed to the genus *Antidorcas*. One of these specimens, GA 36, is only a partial lingual enamel surface that preserves the distinctly V-shaped lingual cusp morphology consistent with the tribe and hypsodonty that excludes *Gazella*, but does not allow for specific diagnosis. The GA 251 lower molar (m2?) is identical in ectoloph and labial cusp morphology to the SKX 28393 *Antidorcas australis/marsupialis* Hendey & Hendey, 1968/Zimmerman, 1780 right m1 from Member 3 and falls within the size range of other *Antidorcas australis/marsupialis* m2 specimens from Swartkrans Members 1 and 2 (Fig. 7A; GA 251: MD: 16.27 mm, BL: 8.56 mm; *Antidorcas australis/marsupialis* MD $\bar x$: 16.07 mm, s.d. 1.96, *n* = 25, BL $\bar x$: 7.98 mm, s.d. 4.42, *n* = 22). The GA 242, GA 248 and GA 253 (Fig. 7B) specimens all exhibit similar ectoloph morphology and degree of hypsodonty with that of several of the Hanging Remnant and Lower Bank *Antidorcas australis/marsupialis* specimens (e.g. SKX 35384; Vrba, 1973, 1976). Linear metric dimensions of both the GDA *Antidorcas* sp. M3 specimens (Fig. 7C; Table S4) show their position within the zone of overlap between *Antidorcas australis/marsupialis* and *Antidorcas recki* Schwarz, 1932 specimens in raw measurements; however, the GD A specimens are minimally inconsistent with assignment to *Antidorcas recki* given the degree of hypsodonty and cusp morphology exhibited in these materials (Vrba, 1973).

Following the extant *Antidorcas marsupialis* dental wear categories of Rautenbach (1971) the GA 251 m2 is within Stage 5c occlusal wear (‘heavy wear’) indicating that the specimen is derived from a fully mature individual of approximately five to seven-and-a-half years of age. Both the GA 242 and GA 253 M3 specimens are only lightly occluded, indicating the presence of two individuals that were likely less than 3.5 years of age (Rautenbach, 1971).

Tribe HIPPOTRAGINI Retzius and Lovén, 1845
Genus HIPPOTRAGUS Sundevall, 1846
Type species *Hippotragus equinus* Geoffroy Saint-Hilaire, 1803
*Hippotragus equinus* Geoffroy Saint-Hilaire, 1803

Referred Specimens. GA 2161, left maxillary fourth premolar; GA 2173, right mandibular second premolar; GA 2174, mandibular left premolar paraconid; GA 2175, left mandibular p3 crown.

Four specimens processed from a single calcified sediment block ‘A’ (of unknown type) represent minimally one *Hippotragus equinus* individual in the GD A deposits. All of these specimens are morphologically identical to modern *Hippotragus equinus* comparative specimens and linear metrics (GA 2173 P2 MD: 14.22 mm, BL: 11.18 mm; GA 2161 P4 MD: 19.3 mm, BL: 17.35 mm; GA 2175 P3 MD: 18.96 mm, BL: 13.53 mm) are consistent with extant *Hippotragus equinus* dental lengths to the exclusion of values reported for *Hippotragus niger* Harris, 1838 (see *Hippotragus* metrics in Klein, 1974). The occlusal wear preserved on the recovered premolar specimens suggest that the individual was likely between 30 and 48 months of age given the unworn state of the GA 2161 P4.

Tribe OREOTRAGINI
Genus OREOTRAGUS Smith, 1834
Type species *Oreotragus oreotragus* Zimmerman, 1783
*Oreotragus* sp.

Referred Specimens. GA 209, left maxilla preserving P2-M2; GA 234, isolated left M1; GA 1960, isolated left P2; GA 2032, articulated lumbar vertebrae (L6, L7), partial pelvis and first caudal vertebra; GA 2033, left proximal metatarsal; GA 2034, partial distal metacarpal metaphysis and diaphysis; GA 2035, left mandible preserving p3-m1; GA 2036, isolated lumber (L5?) vertebra; GA 2038, left distal radius metaphysis and diaphysis; GA 2039, left proximal rib.

**Figure 8:**
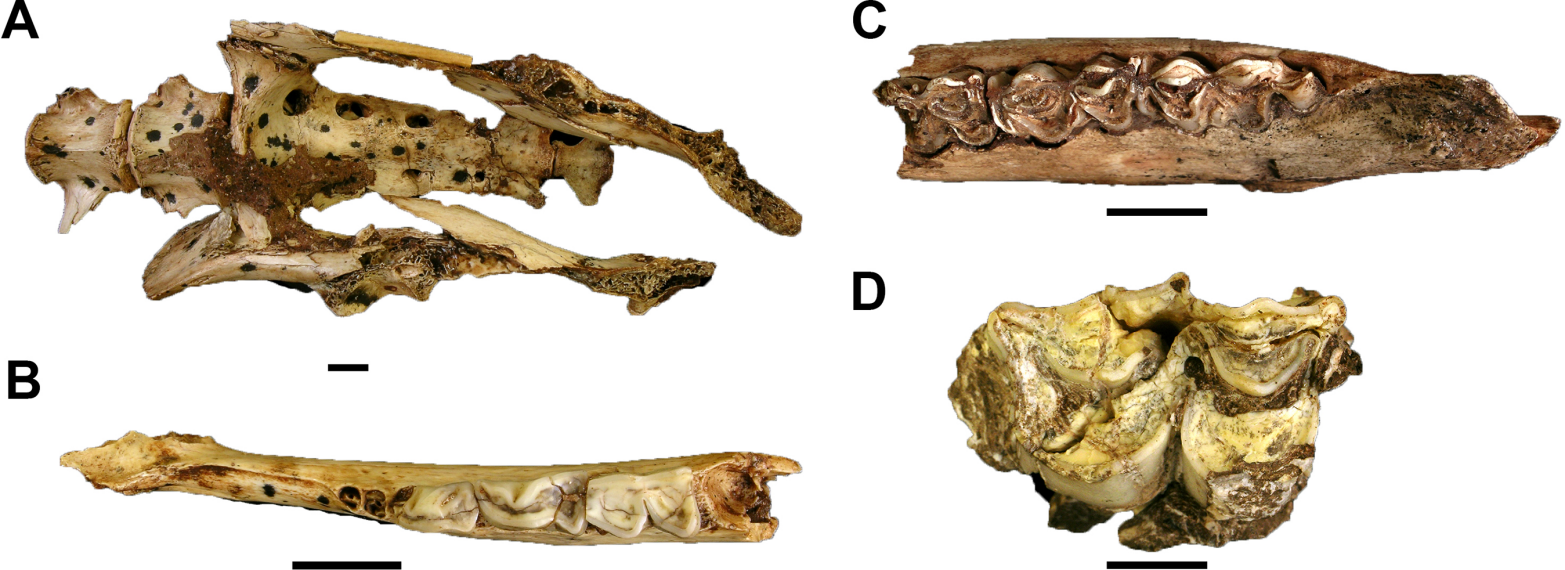
Fossil Oreotragini, Reduncini, and Tragelaphini from Gondolin GD A. (A) *Oreotragus* sp. GA 2032 articulated lumbar vertebrae, partial pelvis, and first caudal vertebra. (B) *Oreotragus* sp. GA 2035 left mandible preserving third premolar to third molar. (C) Reduncini gen. et sp. indet. GA 147 left partial mandible with molar row. (D) *Tragelaphus oryx* GA 1029, right maxillary third molar. Scale bars equal one cm. Photo credits: Justin W. Adams.

Four craniodental and six postcranial specimens, representing at least three individuals (two adult and one immature), from GD A are assignable as *Oreotragus*. The craniodental materials from both the processed and sifted portions of the GD A assemblage are identical metrically and morphologically to those recovered from the Gondolin GD 2 deposits described by Adams & Conroy (2005) and discussed in comparison to other South African oreotragins in Adams (2012b). The postcranial materials (GA 2032 (Fig. 8A), 2033–2034, 2036, 2038–2039) attributed to the taxon based on both morphology and the within-block articulation and association with the GA 2035 *Oreotragus* mandible (Fig. 8B) from a single, small red ‘Finer Clastic Unit’ block (Block 30).

Tribe REDUNCINI Lydekker and Blaine, 1914
Reduncini gen. et sp. indet.

Referred Specimens. GA 8, left maxillary second premolar; GA 20, right partial mandible with molar row; GA 21, left partial mandible with molar row; GA 45, right mandibular second premolar; GA 147, left partial mandible with molar row; GA 170, right maxillary third premolar; GA 175, left maxillary first molar; GA 236, right maxillary third premolar; GA 237, right maxillary third premolar; GA 252, right maxillary third molar; GA 311, right maxillary fourth premolar; GA 311, right maxillary fourth premolar; GA 312, left maxillary fourth premolar; GA 313, right maxillary fourth premolar; GA 868, right mandibular indeterminate position molar; GA 1028, left maxillary deciduous third premolar; GA 2096, left mandibular indeterminate position molar.

Only 16 craniodental specimens from the GD A assemblage are reduncins, with all of these specimens readily accommodated within the genus *Redunca*. Although there is no evidence for the presence of more than one species in the sample, the majority of the recovered *Redunca* remains are isolated teeth which can yield little indication as to a more specific identification for the individual specimens (see Discussion in Adams & Conroy, 2005; Adams, 2006, 2012b). Two of the mandibular remains are sufficiently complete enough to warrant additional description. One of these specimens, GA 21, is a left mandible that preserves an entire molar row. The mandibular depth index value (mandibular depth/molar row length) for the specimen is 0.62, which is slightly below the values for extant *Redunca arundinum* Boddaert, 1785 (0.65) and *Redunca* sp. from Gondolin GD 2 (0.65), but is identical to the value derived from extant *R. fulvorufula* Afzelius, 1815 (Adams, 2006, 2012b). The other mandible, GA 147 (Fig. 8C), has a corpus with a strongly developed mandibular tuberosity that is comparable in size to that seen in both GD 2 *Redunca* sp. and *R. fulvorufula*.

One of the minimally four *Redunca* individuals is from an immature individual of around 1 year of age, based on the light occlusal wear to the GA 1028 deciduous P3 (Spinage, 1967). The other three individuals within the group are from fully mature individuals with permanent premolars and molars in place.

Tribe TRAGELAPHINI Blyth, 1863
Genus TRAGELAPHUS de Blainville, 1816
Type species *Tragelaphus scriptus* Pallas, 1766
*Tragelaphus oryx* Pallas, 1766

Referred Specimens. GA 9, right mandibular second premolar; GA 39, right mandibular indeterminate position molar; GA 1029, right maxillary third molar.

Three dental specimens represent eland from the deposits (treated here as *T. oryx* following Kingdon & Hoffmann, 2013a). The GA 9 right p2 (MD: 13.77 mm, BL: 9.45 mm) exhibits no wear to the occlusal surface, indicating that that the tooth was either in crypt or just erupted when deposited. Following extant eland dental eruptions schedules this would indicate an individual of approximately 36 months of age or younger (Kerr & Roth, 1970). The GA 1029 right M3 (MD: 37.48, BL: 22.67; Fig. 8D) is in an advanced occlusal wear stage, but given the typical eruption age (28 months; Kerr & Roth, 1970) the specimen could be derived from a ∼3-year-old (or older) individual (and therefore not necessarily indicating a second individual present in the deposits. Finally, the GA 39 right indeterminate lower molar, while too damaged for direct metric comparison is visibly similar in size and preserved morphology to mandibular molars of *T. oryx*, and is distinctly larger than comparative extant male *T. strepsiceros* lower molar specimens at a comparable occlusal wear stage.

*Tragelaphus strepsiceros* Pallas, 1766

Referred Specimen. GA 294, left mandible with deciduous fourth premolar and first molar.

Only one specimen, the GA 294 left mandible fragment with the lingual enamel surfaces of the deciduous p4 and m1, could be confidently assigned to *T. strepsiceros*. Although the crowns are too incomplete for standard dental metrics the deciduous p4 compares favourably to modern *T. strepsiceros* juvenile specimens as well as to the G 15572 deciduous p4 from the GD 2 assemblage (Adams & Conroy, 2005; Adams, 2006). The p4 lacks occlusal wear, indicating that the individual was likely not much older than 8 months of age and certainly well under 24 months of age (when the deciduous p4 is shed in this species) (Van Zyl, 1972).

Tragelaphini gen. et sp. indet.

Referred Specimens. GA 13, right mandibular indeterminate position molar; GA 238, left maxillary indeterminate position molar; GA 245, left mandible with second and third molars; GA 247, right probable mandible with partial molars; GA 314, indeterminate side and position maxillary molar; GA 348, left maxillary indeterminate position molar; GA 846, right mandibular third molar; GA 1369, indeterminate side and position mandibular molar; GA 1488, right mandibular third molar; GA 2102, indeterminate side and position mandibular molar.

A collection of ten isolated tragelaphin craniodental specimens represent minimally three further individuals in the GD A sample, but are too incomplete or indeterminate below the tribe level as isolated teeth. While some of these specimens appear similar in overall size to *T. angasii* Gray in Angas, 1848, they equally overlap with some extant comparative *T. imberbis* Blyth, 1869/smaller female *T. strepsiceros* specimens. In particular, three of these specimens (GA 245, 348, and 1488) are more likely from a tragelaphin larger than the average male *T. angasii*. Based on occlusal wear, all of the minimally three individuals in this category were fully mature adults.

Bovidae gen. et sp. indet.

Referred Specimens. See Table S2.

In addition to the bovid remains that could be identified to the tribe level or below, the GD A sample includes 43 bovid craniodental specimens (Tables 1 and 2) and 1,259 indeterminate bovid enamel fragments (Table 1; Table S2).

**Table 2:** Indeterminate bovid craniodental remains from the GD A assemblage.

Element	Bovid I	Bovid II	Bovid III	Indet. bovid
Temporal	1 (R)	5 (3 L, 1 R, 1 I)	2 (I)	–
Maxilla	–	–	–	1 (R)
Upper premolar	–	–	1 (I)	1 (I)
Upper premolar/molar	–	–	–	1 (I)
Upper molar	–	3 (I)	–	4 (I)
Indeterminate molar	–	–	–	2 (I)
Isolated infundibula	–	2 (I)	–	–
Mandible	–	4 (1 L, 2 R, 1 I)	–	2 (1 L, 1 R)
i1	–	2 (1 L, 1 R)	–	–
i2	–	2 (1 L, 1 R)	1 (R)	–
p2	–	–	–	1 (I)
Lower premolar	–	–	1 (R)	2 (1 L, 1 R)
m3	–	–	1 (L)	4 (I)

**Note:**

L, left; R, right; I, indeterminate side.

A listing of the identifiable bovid postcranial remains from the GD A deposits is provided in Table 3. The table includes the bovid Class I postcrania attributed to *Oreotragus* above, but does not include one distal metapodial condyle (GA 905) that was derived from either a Class I or Class II bovid.

**Table 3:** Bovid postcranial remains from the GD A deposits (NISP/MNE/MNI/MAU/%MAU).

Element	Bovid I	Bovid II	Bovid III
Astragalus	–	4/3/2/1.5/75	–
Calcaneus	1/1/1/0.5/50	3/2/2/1/50	–
2/3 Carpal	–	1/1/1/0.5/25	–
Radial carpal	–	1/1/1/0.5/25	–
Distal femur	1/1/1/0.5/50	1/1/1/0.5/25	–
Distal humerus	–	3/2/2/1/50	–
Humerus MSHF	–	1/1/1/0.5/25	–
Distal metacarpal	2/2/1/0.5/50	2/2/1/0.5/25	–
Proximal metacarpal	1/1/1/0.5/50	2/2/2/1/50	2/2/1/1/100
Distal metapodial	–	6/4/1/1/50	1/1/1/1/100
Metapodial MSHF	–	4/2/1/1/50	–
Distal metatarsal	–	1/1/1/1/50	–
Metatarsal MSHF	–	3/2/1/1/50	–
Pelvis	4/2/2/1/100	3/2/2/1/50	–
Phalanx 1	3/2/1/0.25/25	2/2/1/0.25/12.5	3/3/1/0.75/75
Phalanx 2	1/1/1/0.125/12.5	6/6/1/0.75/37.5	–
Phalanx 3	–	1/1/1/0.125/6.3	–
Distal radius	1/1/1/0.5/50	1/1/1/0.5/25	1/1/1/0.5/50
Radius MSHF	–	1/1/1/0.5/25	–
Proximal Rib	1/1/1/0.04/4.0	–	–
Sacrum	1/1/1/1/100	–	–
Scapula	–	2/2/1/1/50	–
Distal sesamoid	–	3/3/1/0.188/9.4	–
Proximal sesamoid	–	1/1/1/0.125/6.3	–
1st Tarsal	–	–	1/1/1/0.5/50
2/3 Tarsal	–	1/1/1/0.5/25	–
Tibia MSHF	–	1/1/1/0.5/25	–
Proximal tibia	–	1/1/1/0.5/25	–
Proximal Ulna	–	1/1/1/0.5/25	–
Atlas	–	2/2/2/2/100	–
Axis	–	1/1/1/1/50	–
Cervical vertebrae	–	1/1/1/0.143/7.2	–
Thoracic vertebrae	–	2/1/1/0.071/3.6	–
Lumbar vertebrae	6/4/1/0.667/80	5/3/1/0.5/25	–
Caudal vertebrae	1/1/1/0.08/8.0	–	–

Family GIRAFFIDAE Gray, 1821
GENUS GIRAFFA Brisson, 1762
Type species *Giraffa camelopardalis* Linneaus, 1758
*Giraffa* sp.

Referred Specimen. GA 308, maxillary deciduous third premolar.

**Figure 9:**
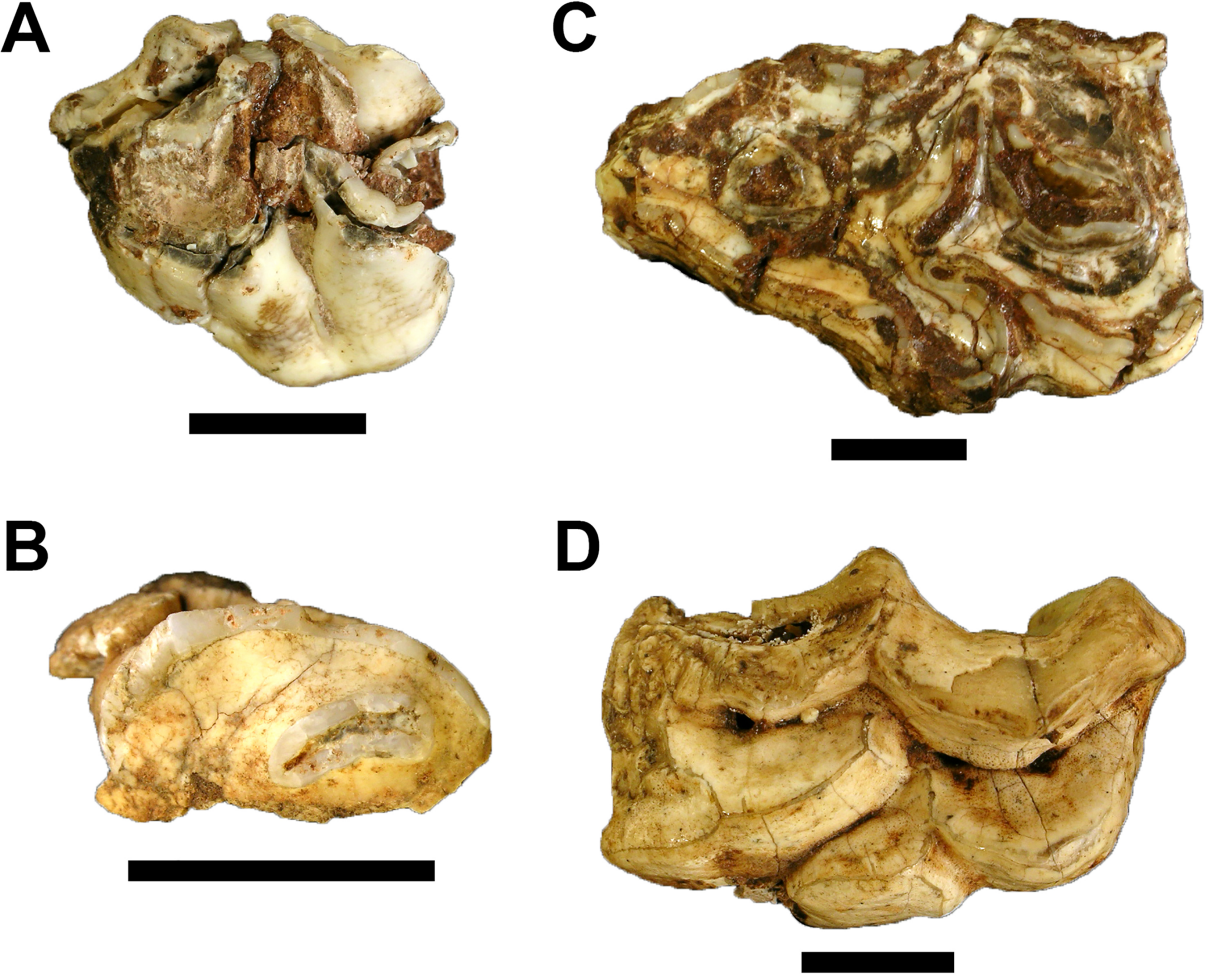
Fossil Giraffidae, Suidae, and Equidae from Gondolin GDA. (A) *Giraffa* sp. GA 308, maxillary deciduous third premolar. (B) Suidae gen. et sp. indet. GA 164, partial indeterminate side and position crown fragment. (C) *Equus capensis* GA 93, left mandibular deciduous second premolar. (D) *Equus capensis* GA 2026, right maxillary deciduous indeterminate position premolar. Scale bars equal one cm. Photo credits: Justin W. Adams.

This single specimen is the only giraffid recovered both from the GD A deposits and the Gondolin locality overall; and one of few giraffid specimens from the penecontemporaneous South Africa karstic deposits (Fig. 9A; Brain, 1981; Reed, 1996; Watson, 1993a). The specimen is damaged on its buccal surface and exhibits some matrix-infiltration distorting the BL diameter of the crown. The specimen is morphologically analogous to deciduous P3s of extant *Giraffa* and exhibits characteristic rugosities to the external enamel surface. Although confident dental metrics cannot be gathered, the preserved BL width (enlarged by matrix) of 20.30 mm and minimum MD length of 22.24 mm (estimated maximum MD <26 mm) is consistent with values reported for *Giraffa* and substantially smaller (particularly in MD length) than described dP3s of *Sivatherium* (e.g. KNM 2627: MD: 40.7 mm, BL: 33.1 mm; Harris, 1991; giraffid mean values in Robinson, 2011). The deciduous P3 erupts at 9 days of age and is shed after 4 years; the light occlusal wear to the anterior set of cusps suggests that the specimen is likely from an individual less than a year of age (Singer & Bone, 1960).

Family SUIDAE Gray 1821
Suidae gen. et sp. indet.

Referred Specimens. GA 164, partial indeterminate side and position crown fragment; GA 2123, partial probable third molar talon/talonid fragment; GA 2141, partial probable third molar talon/talonid fragment; GA 2156, partial indeterminate side and position crown fragment.

In contrast to the robust suid assemblage present in the GD 2 in situ assemblage, only four indeterminate suid enamel fragments have been recovered from the GD A deposits (Adams & Conroy, 2005). The GA 2156 enamel fragment is the least diagnostic of the collection, consisting of a small portion of enamel with a distinctive sigmoidal appearance that is most consistent with a hypsodont suid tooth crown but is otherwise indeterminate. The GA 164 specimen preserves a small part of the occlusal surface (with an ‘enamel island’) and part of the thick external crown enamel with a thick layer of cementum (Fig. 9B). The specimen is similar in both enamel and cementum thickness to metridiocharin second molar specimens from the GD 2 assemblage (Adams & Conroy, 2005). The two remaining specimens (GA 2123 and GA 2141) are probable third molar terminal pillars with thick enamel and a distinctly sigmoidal curvature as they approach the occlusal surface (i.e., unworn on the GA 2123 specimen and superficially worn on the GA 2141 specimen). Both of these specimens are identical in terms of size, enamel thickness and curvature to the ‘late stage’ (e.g., Stage III *sensu* Harris & White, 1979) *Metridiochoerus andrewsi* Hopwood, 1926 specimens that are also recovered in the GD 2 assemblage; however, at most these specimens can be considered simply more consistent with a metridiocharin suid than with any other extinct or extant suid lineage like warthog (*Phacochoerus* sp. Cuvier, 1817) or bushpig (*Potamochoerus porcus* Linneaus, 1758).

Order PERISSODACTYLA Owen, 1848
Family EQUIDAE Gray, 1821
Genus EQUUS Linnaeus, 1758
Type species *Equus caballus* Linnaeus, 1758
*Equus capensis* Broom, 1909

Referred Specimens. GA 93, left mandibular deciduous second premolar; GA 307, partial left indeterminate position probable premolar; GA 2169, right indeterminate position premolar or molar; GA 2026, right maxillary deciduous indeterminate position premolar.

Of the entire GD A assemblage equid sample, only four specimens derived from at least three different individuals can be confidently attributed to the extinct giant cape horse, *E. capensis*. The GA 93 specimen is a deciduous left P2 that is heavily worn and likely close to being shed given the heavily resorbed state of the roots (Fig. 9C). Although damaged along the enamel surface, the measurements (MD: 40.17 mm, protocone length: 7.63 mm) is consistent with *E. capensis* deciduous P2 specimens from Kromdraai A (KA 1897: MD: 40.2 mm, PL: 7.4 mm) and the Sterkfontein Type Site (STS 3002A: MD: 41.5 mm, PL: 8.8 mm). The GA 307 left (probable) premolar preserved only part of the distal aspect of the occlusal surface. Overall, the specimen is visibly massive relative to extant equid dental specimens and records a protocone length (11.98 mm) consistent with the mean protocone lengths for *E. capensis* P3 and P4 specimens from Kromdraai, Minaar’s, Coopers, Swartkrans, and Sterkfontein (P3: $\bar x$: 11.31 mm, *n* = 17; P4: $\bar x$: 12.08 mm, *n* = 13; Churcher, 1970). The GA 2026 right deciduous upper premolar (P3 or P4; Fig. 9D) lacks occlusal wear, and is consistent in both morphology and dimensions (MD: 36.23 mm, BL: 23.74 mm, protocone length: 11.05 mm) with *E. capensis* deciduous P3 and P4 specimens from the other South African assemblages (P3: MD $\bar x$: 33.15 mm, *n* = 6; BL $\bar x$: 24.58 mm, *n* = 5; PL $\bar x$: 10.66 mm, *n* = 5; P4: MD $\bar x$: 32.5 mm, *n* = 5; BL $\bar x$: 22.55 mm, *n* = 2; PL: 12.3 mm, *n* = 1; Churcher, 1970). Finally, the GA 2169 specimen is a right upper premolar or molar, likely a P4, M1 or M2 with light-moderate occlusal wear (Stage 1 *sensu* Eisenmann et al., 1988). The specimen (MD: 26.63 mm, BL: 28.10 mm, protocone length: 11.37 mm, height at parastyle: 56.03 mm) falls within the size range for *E. capensis* M1 and M2 specimens from the other South African fossil assemblages (M1: MD $\bar x$: 30.39 mm, s.d. 3.22, *n* = 12; BL $\bar x$: 28.84 mm, s.d. 1.33, *n* = 12; PL $\bar x$: 11.48 mm, s.d. 1.83, *n* = 8).

The advanced occlusal wear on the GA 93 deciduous P2 is consistent with being derived from an individual just under 3 years of age, as this is when the tooth is typically shed (Fig. 9C). The lack of occlusal wear on the GA 2026 deciduous upper premolar, however, indicates that the specimen must be derived from an individual only a few months of age, as the upper premolars are typically in wear within the first few weeks postpartum (Fig. 9D; Klingel, 1965).

*Equus* sp.

Referred Specimens. GA 82, indeterminate side or position distal third metapodial; GA 83, indeterminate side or position distal third metapodial; GA 92, right mandibular second premolar; GA 156, left partial mandible with parts of two premolars and/or molars; GA 303, right mandibular first incisor; GA 309, indeterminate side maxillary incisor root; GA 315, left external cuneiform; GA 338, indeterminate side or position proximal second or fourth metapodial; GA 1780, right mandibular first or second incisor; GA 1858, left mandibular premolar or molar crown; GA 2126, partial indeterminate side maxillary premolar or molar crown.

In addition to the equid specimens attributable to *E. capensis* there are a further seven craniodental and four postcranial specimens that could not be confidently assigned below the generic level. Of the craniodental specimens, only two preserve sufficient morphology to discuss here. The GA 156 specimen is a partial left mandible preserving part of two lower molars (likely the m1/m2 rather than the m2/m3). The teeth themselves are heavily worn and damaged, and although the specimens lack ectosylids, the ectoflexids are deep and nearly reach the isthmus, and the linguaflexid is both shallow and not as pointed as in some *Equus* species. The GA 96 p2 was in occlusion, indicating an individual of minimally 3 years of age.

The postcranial sample is poorly preserved and/or damaged which limited metric comparison. Morphologically, the specimens are inconsistent with hipparionin postcrania from Swartkrans Members 1 and 2 and are visibly larger in overall preserved size (Watson, 1993a). The specimens are (minimally) as large as postcranial materials derived from larger modern *E. burchelli* (*sensu* Kingdon & Hoffmann, 2013b) individuals and are morphologically consistent with extant and fossil *Equus* comparative specimens.

Order HYRACOIDEA Huxley, 1869
Family PROCAVIIDAE Thomas, 1892
Genus PROCAVIA Storr, 1780
Type species *Procavia capensis* Storr, 1780
*Procavia* sp.

Referred Specimens. GA 1342, right female maxillary first incisor; GA 1943, right mandibular deciduous or permanent second premolar; GA 1990, right maxillary third or fourth premolar; GA 2160, left mandibular second incisor.

There is only a very small sample of isolated hyrax teeth from the GD A assemblage. Although none of the specimens can be specifically attributed due to their isolation or preservation, they are visibly more consistent with a smaller species of hyrax (e.g. *Procavia antiqua* Broom, 1934 or *Procavia capensis*) than the larger extinct hyrax, *Procavia transvaalensis* Shaw, 1937. The GA 1342 specimen clearly preserves the labially rounded crown surface that is consistent with having been derived from a female hyrax (Churcher, 1956).

Order RODENTIA Bowdich, 1821
Family HYSTRICIDAE Fischer de Waldheim, 1817
Genus HYSTRIX Linnaeus, 1758
Type species *Hystrix cristata* Linnaeus, 1758
*Hystrix africaeaustralis* Peters, 1852

Referred Specimen. GA 6, left indeterminate position maxillary premolar or molar.

This single specimen, representing a complete left P4 or (probable) M1 is the only representative of the extant African porcupine. The crown is only lightly occluded, lacks roots, and is comparable in size to modern *Hystrix africaeaustralis* M1 specimens.

*Hystrix makapanensis* Greenwood, 1958

Referred Specimens. GA 7, left mandibular fourth premolar; GA 11, right maxillary first molar; GA 50, left maxillary probable third molar; GA 839, left mandible with fourth premolar.

The GD A assemblage *Hystrix makapanensis* specimens have been previously fully described and compared in Adams (2012a).

Hystricidae gen. et sp. indet.

Referred Specimens. GA 12, indeterminate side and position premolar or molar; GA 77, probable right maxillary incisor and alveolus; GA 320, indeterminate side and position incisor; GA 1915, indeterminate side and position incisor.

A small collection of incomplete dental specimens from the GD A deposits were too incomplete to assign to genus or species. The GA 12 specimen may be derived from a *Hystrix makapanensis* individual as the specimen is large (MD: 9.15 mm, BL: 7.95 mm) in comparison to the mean values for premolars and molars of extant *Hystrix africaeaustralis* (but falls within the upper boundary of some extant individuals; see comparative metrics in Adams, 2012a).

Order LAGOMORPHA Brandt, 1855
Family LEPORIDAE Fischer von Waldheim, 1817
Leporidae gen. et sp. indet.

Referred Specimens. GA 69, left proximal ulna; GA 2189, left mandible with third premolar.

Only two specimens from minimally one individual could be attributed to the Family. Both specimens are similar in size and morphology to *Pronolagus rupestris* Smith, 1834 comparative specimens and other leporid specimens from the Gondolin, Haasgat, and Drimolen Main Quarry deposits (Adams, 2006; Adams, 2012b; Adams et al., 2016). Unfortunately there is light matrix adhering to the occlusal surface of the GA 2189 p3 which prohibits assessment of reentrant morphology and facilitating clear diagnosis to the genus *Pronolagus.*

Mammalia gen. et sp. indet.

Referred Specimens. See Table S2.

The remainder of the GD A sample could not be taxonomically attributed to any particular macromammalian Order (Table 1; Table S2). This includes 650 indeterminate enamel fragments (nine possible Family Bovidae, seven possible Family Bovidae or Family Equidae), 28 indeterminate cranial fragments (including 15 mandibular or maxillary alveolar fragments, 12 vault fragments and one possible premaxilla), three indeterminate petrosals (two of which may be from a class II bovid, and one that is similar in size to a class III bovid or equid), one indeterminate mandibular fragment, and one indeterminate maxillary fragment.

There were also 33 indeterminate mammalian postcranial specimens, including: one carpal or tarsal fragment (GA 1742), one unfused proximal humeral head (GA 2178), one small mammal distal metapodial (GA 66), one small mammal proximal metatarsal (GA 65), an indeterminate diaphysis from a large bodied mammal (e.g. similar to class III/IV or larger) (GA 88), one pelvic fragment (GA 144), one distal rib (GA 167), seven rib shaft fragments (GA 126, 192, 755, 1805, 1810, 1874, and 1891), four proximal ribs (GA 720, 1366, 1694, and 1740), one scapular glenoid fossa from a large bodied mammal (e.g., similar to a class III/IV bovid or larger mammal) (GA 1429), one small mammal left proximal tibia (GA 1807; possible *Procavia* sp.), eight vertebral fragments (GA 744, 757, 1391, 1496, 1813, 1820, and 1866, indeterminate; GA 145, small mammal), two caudal vertebrae (GA 57 and 1876; possibly a class II bovid), two lumbar vertebrae (GA 166, possibly a class II bovid; GA 1961), and one thoracic vertebrae (GA 1862, possibly a class II bovid).

The remainder of the indeterminate macromammal specimens are not identifiable to element, largely representing indeterminate diaphysis fragments (Table S2).

Class AVES Linneaus 1758
Aves gen. et sp. indet.

Referred Specimens. GA 158, right proximal femur; GA 326, indeterminate side distal humerus; GA 1360, left proximal femur; GA 1497, right distal femur.

As noted in the ‘Materials and Methods,’ no attempt was made to diagnose the four avian specimens below the level of the Class. These four specimens are derived from minimally three individuals based on the occurrence of equivalent or overlapping elements and overall size differences, with one individual (GA 158) comparable in size to a modern medium sized duck (Family Anatidae Leach, 1820) or barn owl (Family Tytonidae Ridgway, 1914), another (GA 1497) that is somewhat larger (comparable in size to a modern swan, Family Anatidae) and the third (GA 326, GA 1360) being somewhat smaller in size than the other two individuals.

### Additional GD A faunal assemblage characteristics

The McIntosh diversity index (McIntosh, 1967) for the total GD A faunal assemblage is 0.90 (based on MNI; not including bovid individuals classified only to Tribe). The carnivore-ungulate ratio (*sensu* Klein & Cruz-Uribe, 1984) for the GD A faunal assemblage is 9.1%.

As noted above, the GD A deposits are an artificially heterogeneous mixture of at least two different types of calcified sediments (and presumably associated decalcified sediments). As such, the GD A fossil assemblage described has been shaped by at least three different taphonomic processes: those that operated on fossil materials during the original deposition of the two calcified sediment types, and the post-depositional action of the miners who removed the calcified sediments from their original context, mixed, and redeposited the materials in the surface dumps. While the mixed origin of the GD A sample and loss of in situ context could be seen as precluding any taphonomic analysis, such primary data does allow for evaluation of basic sample characteristics like patterns of biotic and abiotic modification, element representation/abundance in light of the previously described (and strongly contrasting) taphonomic histories of the GD 1 and GD 2 assemblages. Although I will offer some basic taphonomic data on the GD A assemblage that treat the sample as a unit, the interpretation of such results can only proceed with the heterogeneous nature of the fossil assemblage into account (see Discussion in Adams & Rovinsky, in press). Equally, some assessment of the patterns of element representation between fossils derived from the two matrix types, or between fossils from the calcified and naturally decalcified sediments, could not be undertaken because of the low sample sizes partitioned across these intrasample divisions prohibited meaningful comparisons across subdivisions of the total GD A assemblage.

*Peri- and post-mortem element modification and weathering*. A total of 102 specimens (0.67% of the total GD A sample) exhibited signs of peri- or post-mortem biotic modification by carnivores, rodents, borer beetles, or tree roots. None of the recovered remains showed indications of pathology. Only one specimen showed signs of being possibly modified by hominins.

Carnivore-induced modification was noted on a total of 76 specimens, although 30 of these were classified as only having been probably modified by carnivores. The total breakdown of carnivore modification types is as follows: one specimen with acid-pitting consistent with having been digested, 22 specimens exhibit small-diameter shallow pitting, 28 specimens preserve tooth marks, 12 specimens show signs of gnawing, 11 specimens have combined shallow pitting, tooth marks and/or gnaw marks, and two specimens show deep puncturing. All carnivore modifications were located on unidentifiable diaphysis fragments, except for a single bovid class II left ilium (GA 336) that was punctured, and two of the gnawed specimens (GA 793, class II bovid metapodial diaphysis; GA 782, class I bovid distal first phalanx). Nearly all of the carnivore modified specimens (*n* = 61) were derived from the sifted, naturally decalcified sediments or processed breccias (*n* = 7) without a known matrix type. The remaining eight carnivore modified specimens were derived from the grey (*n* = 5; all indeterminate diaphysis fragments with tooth scores or gouges) and red (*n* = 3; two indeterminate diaphyses with tooth scores and the GA 2007 left bovid class II astragalus with a small-diameter puncture) matrix blocks.

Small rodent gnawing is apparent on two specimens, one an unidentifiable diaphysis fragment (GA 750) and the other an isolated carnivore canine (GA 2129). Both were derived from the sifted, naturally decalcified specimens. Distinct porcupine gnawing was noted on 20 specimens, 16 of which are unidentifiable diaphysis fragments. The four identifiable remains with porcupine gnawing include a right indeterminate alcelaphin mandible (GA 2003), a left *Redunca* mandibular ramus (GA 147), a class I bovid acetabulum (GA 1930), and a class II bovid left glenoid process (GA 85). Two of the unidentifiable diaphyses with porcupine gnawing also have heavily eroded cortices. Half of the porcupine modified specimens (*n* = 10) were derived from sifted, naturally decalcified sediments and five from processed breccias of unknown matrix type. The remaining five specimens were derived from the grey (*n* = 1; GA 2019 indeterminate diaphysis fragment) and red (*n* = 4; including G 147, G 1930, G 2003 (see above), and G 2009 indeterminate diaphysis fragment) matrix blocks. Borer beetle perforations were found on four specimens, all of which are indeterminate diaphysis fragments (GA 101, 159, 728, 1827). Two of these specimens (GA 101, 728) are from the sifted, naturally decalcified sediments (GA 101 and 728), while the other two are from the grey (GA 159) and red (GA 1827) matrix blocks.

**Figure 10:**
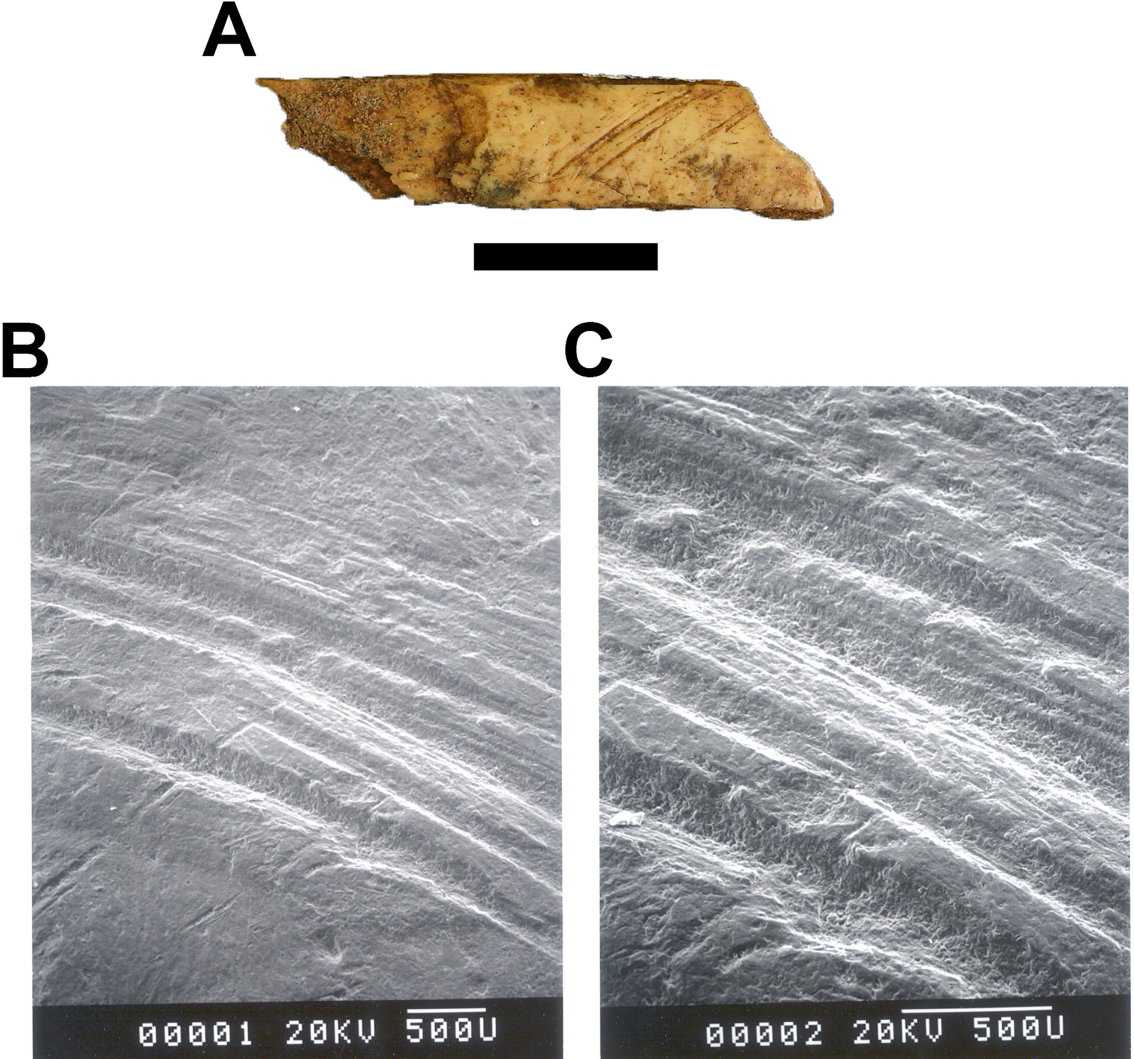
The GA 763 indeterminate mammal diaphysis fragment. (A) External cortical surface of the specimen, scale bar equals one cm. (B) 25× magnification SEM image, scale bar equals 0.5 mm. (C) 50× magnification SEM image, scale bar equals 0.5 mm. Photo credits: Justin W. Adams.

A single indeterminate mammal diaphysis fragment (GA 763, three to four cm overall size category) recovered from the GD A decalcified sediments preserves what was initially identified as representing hominin-mediated stone tool marks on the external cortical surface (Fig. 10A). A series of six large, parallel marks are visible on the specimen, concentrated near the middle of the preserved external cortex. There are a number of smaller marks that run in the same direction to (and are interspersed between) the larger marks. Under SEM magnification (Figs. 10B and 10C), the marks are relatively shallow and wide, and do not have delineated fine striae on the walls of the marks. Also visible in the SEM images are marks that cut across the cortical surface and the primary, parallel marks. The microscopic morphology of the specimen, combined with the small size of the specimen and the lack of any anatomical context, prevent confirming the specimen as having been modified by hominins. A more parsimonious interpretation is that the marks were produced by abrasive contact with another object at some point during the pre- or (extensive) postdepositional phases of the decalcified GD A sediments (Andrews, 1995).

Weathering stage data (*sensu* Behrensmeyer, 1978) were gathered from 2,448 specimens from the total GD A assemblage and are summarized in Table 4. A series of Kolmogorov–Smirnov tests (α = 0.05) found no statistically significant differences between the weathering stages of the sifted fossil materials and those from the processed blocks (*D* = 0.4, *p* = 0.697), between the identifiable postcranial remains and the indeterminate diaphysis fragments (*D* = 0.4, *p* = 0.697), or between the fossils processed from the red and grey calcified sediments (*D* = 0.25, *p* = 0.997).

**Table 4:** Weathering stage data from the GD A deposits by total and subcategory.

Stage	Total	Sifted	Processed	‘Red’ breccias	‘Grey’ breccias	Indeterminate fragments	Postcrania
0	1,908	203	1,705	978	465	1,810	98
1	461	28	433	222	163	417	44
2	63	12	51	15	15	61	2
3	15	4	11	2	3	13	2
4	1	1	–	–	–	1	–
5	–	–	–	–	–	–	–
Total	2,448	248	2,200	1,217	646	2,302	146

*Fragmentation, shaft attributes and pre- and postdepositional destruction*. There is insufficient data from the GD A assemblage to apply several measures of fragmentation or postdepositional destruction analyses (see Discussion in Adams & Rovinsky, in press). As such, taphonomic analysis of these features of the assemblage is necessarily limited to simple size category data, long bone fracture features (*sensu* Villa & Mahieu, 1991), and the calculation of the NISP:MNE (after Richardson, 1980) and bovid carpal and tarsal Completeness Indices (after Marean, 1991).

Size category data were taken from all 15,250 specimens from the GD A assemblage, and the distribution of specimens across size categories and subsets of the total assemblage are summarized in Table 5. A series of Kolmogorov–Smirnov tests (α = 0.05) found no statistically significant differences in the distribution of size categories between the assemblage sifted from decalcified sediments and that processed from calcified sediment blocks (*D* = 0.214, *p* = 0.862), or between the fossils recovered from the red and the grey calcified sediment blocks (*D* = 0.429, *p* = 0.111). There were also no statistical differences detected between the identifiable and unidentifiable specimens (*D* = 0.286, *p* = 0.541), the bovid enamel fragments and the indeterminate enamel fragments (*D* = 0.143, *p* = 0.997), or the identifiable craniodental and postcranial specimens (*D* = 0.214, *p* = 0.862).

**Table 5:** Size category data from the GD A deposits.

Size category	GD A total	Total sifted	Total processed	‘Grey’ breccias	‘Red’ breccias	Bovid enamel fragments	Indeterminate enamel fragments	Indeterminate bone fragments	Identifiable craniodental	Identifiable postcranial
0–1 cm	5,455	1,309	4,146	1,455	1,899	129	442	4,869	6	9
1–2 cm	7,553	5,444	2,109	916	737	873	180	6,392	73	35
2–3 cm	1,653	1,159	494	172	137	208	22	1,307	86	30
3–4 cm	374	245	128	33	36	42	5	263	42	21
4–5 cm	121	59	62	14	16	6	1	75	20	19
5–6 cm	42	16	26	–	3	1	–	23	8	10
6–7 cm	24	6	18	–	4	–	–	16	5	3
7–8 cm	14	2	12	–	3	–	–	6	3	5
8–9 cm	8	4	4	1	1	–	–	4	1	3
9–10 cm	1	–	1	–	–	–	–	–	1	–
10–11 cm	3	–	3	–	2	–	–	2	–	1
∼										
12–13 cm	1	–	1	–	1	–	–	–	–	1
16–17 cm	1	–	1	–	1	–	–	–	–	1
20–21 cm	1	1	–	–	–	–	–	1	–	–
Total	15,250	8,245	7,005	2,591	2,840	1,259	650	12,958	245	138

Fracture feature data were collected from a total of 29 specimens from the GD A assemblage and are summarized in Table 6. The three fracture features were derived from the one class II bovid femur, eight unidentifiable diaphyses, three class II bovid humeri, eight metacarpals (three class I, four class II, and one class III bovid), three metapodials (one class II bovid, and two equids), three metatarsals (all class II bovids), two radii (one class I and one class II bovid) and one class II bovid tibia. Because of missing data from some of the subdivisions of the total assemblage, only a limited set of intraassemblage χ^2^ tests (α = 0.01) could be conducted. No significant differences were found in the distribution of fracture outlines between the sifted and the processed subsets (χ^2^ = 0.207, *p* = 0.904) or the red and the grey calcified sediment subsets (χ^2^ = 1.238, *p* = 0.539). Similarly, there was no significant difference in the distribution of preserved bone circumferences between the processed and sifted subsets (χ^2^ = 5.77, *p* = 0.056). A further series of interassemblage χ^2^ tests (α = 0.01) of the total GD A fracture feature values against the Sarrians, Fontbrégoua, and Bezoce assemblages described by Villa & Mahieu (1991) found significant differences with all three assemblages in fracture angle and circumference, but no significant difference in fracture outlines with any of the three outgroup samples.

**Table 6:** Fracture feature data from the GD A assemblage.

GD A Total Assemblage				Sifted Total			
Category	Angle	Outline	Circumference	Category	Angle	Outline	Circumference
1	16	12	12	1	10	5	9
2	11	12	4	2	4	7	1
3	2	5	13	3	–	2	4

Processed total				‘Red’ breccias			
1	10	9	5	1	4	3	2
2	9	9	4	2	–	1	–
3	2	3	12	3	–	1	2

‘Grey’ breccias							
1	1	1	1				
2	3	2	1				
3	–	1	2				

**Note:**

Category Legend: Angle: 1, oblique; 2, right; 3, oblique and right; Outline: 1, transverse; 2, curved/V-hape; 3, intermediate; Circumference: 1, <50%; 2, >50%, 3, 100%.

Two indices to assess fragmentation were calculated from the GD A fossil assemblage. The Completeness Index (CI) was calculated for eight different elements: class I bovid calcanei (CI = 33); class II bovid astragali (CI = 58.25), calcanei (CI = 50), 2nd/3rd carpals (CI = 90), radial carpals (CI = 90), and 2nd/3rd tarsals (CI = 75); class III bovid 1st tarsals (CI = 100); and equid external cuneiforms (CI = 75). The NISP:MNE index was calculated for the 11 groups and elements originally identified by Richardson (1980). The index results for the GD A assemblage were: Long bones = 119.35; Small bones = 110.34; Cranial = 200; Mandible = 146.15; Scapula = 100; Cervical vertebrae = 100; Thoracic vertebrae = 200; Lumbar vertebrae = 157.14; Ribs = 100; Pelvis = 175.0; Sacrum = 100. Small sample sizes for these elements prohibited evaluating these fragmentation measures across the different matrix types within the GD A samples.

*Postdepositional consumption, utility and transport*. Visual inspection of the percentage minimal animal unit (%MAU) values from the GD A total bovid assemblage (Table 3) does superficially resemble the inverse carnivore consumption sequences described by Blumenschine (1986). There is, however, no significant correlation between the rank-ordered elements in the consumption sequence and the summed bovid %MAU values (*r_S_* = −0.202, *p* = 1.00), or with the %MAU values from the three bovid size classes with substantial skeletal representation in the assemblage (class I: *r_S_* = −0.501, *p* = 0.117; class II: *r_S_* = −0.009, *p* = 0.982; class III: *r_S_* = 0.763, *p* = 0.133). Any resemblance appears to be due solely to the high %MAU values of the recovered bovid mandibles.

The GD A total bovid assemblage %MAU values were regressed onto percentage modified general utility index (%MGUI) and unstandardized food utility index (FUI) values reported by Binford (1978) and Metcalfe & Jones (1988). The regression of the GD A total bovid %MAU on %MGUI values revealed no significant relationship between the two variables (*r_S_* = −0.292, *p* = 0.177), and visual inspection of the data scatterplot does not resemble any of the patterns previously identified by Binford (1978) based on modern human meat foraging strategies. There is also no significant relationship between the GD A total bovid %MAU and the Metcalfe and Jones FUI values (*r_S_* = −0.200, *p* = 0.359). Visual inspection of the scatterplot of these two variables reveals that the datum points are scattered both above and below an isometric line (used to represent the trend in data expected in an unbiased utilization strategy).

Similar to the consumption sequence results above, total bovid %MAU values followed no visibly discernable trend relative to the Hill (1979) disarticulation sequence. There is no significant correlation between the rank-ordered disarticulation sequence and the summed bovid %MAU from the GD A assemblage (*r_S_* = −0.110, *p* = 0.608), or to the %MAU values reported for the class I (*r_S_* = 0.148, *p* = 0.492) or class II (*r_S_* = −0.452, *p* = 0.068) bovids. Only five class III bovid elements from the consumption sequence have been recovered, which has likely influenced the significant negative correlation found between %MAU values and the sequence (*r_S_* = −0.975, *p* = 0.005).

There is also no significant correlation between the total bovid %MAU values from the GD A assemblage and the saturated weight index values for *Redunca* skeletal remains (*r_S_* = 0.148, *p* = 0.489). Visual inspection of a scatterplot of these two variables reveals (minimally) two outlying points (representing values for bovid phalanges and scapulae); however, Cook’s Distance values for the each of the datum points in the correlation revealed that neither the phalanges (D: 0.212) nor scapulae (D: 0.006) were unduly influencing the regression.

*Density mediated attrition*. A regression of log10 transformed %MAU values from the total GD A identifiable postcranial remains on bovid element-wise bone mineral density values found no significant correlation between the two variables (*r_S_* = 0.174, *p* = 0.358). A plot of standardized residual values on predicted values of the dependent variable indicates that the variation was homogeneously distributed.

## Discussion

As an artificially aggregated ex situ deposit, the GD A sample understandably cannot provide readily interpretable assemblage faunal or taphonomic data relative to either of the in situ Gondolin assemblages (GD 1 or GD 2) or other penecontemporaneous in situ South African karstic samples (see also Discussion in Adams & Rovinsky, in press). That said, the complex development of the GD A assemblage is not entirely dissimilar to that of some South African karst-derived samples (like those from Sterkfontein, Swartkrans, Kromdraai, and Bolt’s Farm), that were historically developed by selectively sorting through ex situ miner’s dumps (see Discussion in Brain, 1981; De Ruiter, 2001; Adams, 2006). There are several factors that make it unclear whether historical associations drawn between ex situ calcified sediments and the original cave stratigraphy ultimately reduced or potentially exaggerated spatial or time-averaging of such South African fossil assemblages. First, our understanding of speleogenesis, deposit formation and cave system taphonomy has radically advanced in the last few decades, highlighting the complexity of formed assemblages and the site-specific challenges of treating deposits defined by gross matrix appearance as homogenous units for analysis (see Discussion (among others) in Herries, 2003; Herries & Shaw, 2011; Pickering et al., 2011; Herries et al., 2013, 2014). Second, the grouping of fossils from ex situ sampling and in situ excavations into faunal assemblages, particularly across decades of varying collection protocols at some cave sites, may yield artificial analysis results—from creating associations between specimens/species, to overly homogenous taphonomic interpretations for temporally or spatially extensive depositional units, to ‘mosaic’ heterogeneous palaeoecosystems constructed from such aggregated faunal data (Brain, 1981; Reed, 1996; De Ruiter, 2001; Adams, 2006; Adams & Rovinsky, in press). And third, preparation methods have largely eliminated the geological matrix that formed the basis for the original association of ex situ materials to in situ regions and depositional units; this eliminates the potential for reevaluation in light of such revised karstic histories for the ex situ specimens and forces us to rely on field notes and catalogue records that have varied in detail and extent. Hence why some Quaternary Bloubank Valley fossils from these early phases (including several faunal type specimens) have ambiguous provenance (see Discussion in Ewer, 1955a, 1955b; Cooke, 1991; see Adams et al., 2015; Table S1 for specific examples). These outstanding issues in South African palaeontology may prompt the exploration of new methods like direct sampling for rare earth elements to establish provenance between fossil specimens and localities/deposits, as well as between fossil specimens across deposits to evaluate time and spatial averaging within deposits (Trueman et al., 2005; see also Discussion in Grün et al., 2010).

While potentially sharing similar sources of bias or interpretive caveats with other South Africa Plio-Pleistocene assemblages, there are nonetheless two fundamental differences between the GD A and other penecontemporaneous deposit samples. First is that the GD A sample explicitly contains fossils from at least two distinctly different phases of sedimentation, and decalcified fossils that may be derived from one, both, or an additional deposit(s) from the Gondolin site. The second is that the extensive mining-mediated destruction of the Gondolin karstic system prohibits direct correlation (at present) of the red and grey calcified sediment type in Dump A to any particular depositional phase represented by the modern in situ fossil-bearing remnants. In fact there is no particular basis for assuming that fossils derived from either the GD A red or grey calcified sediments have a shared taphonomic history or are chronological or spatially associated, even if they shared one or more sedimentation events within the karstic system or overall ‘matrix type.’

With these interpretive issues in mind, the total GD A faunal assemblage derived from both calcified sediment types and sifted decalcified sediments includes specimens of Order Primates and three mammalian Families (Families Felidae, Herpestidae, and Giraffidae); no representatives of these groups have been described from either the well-sampled GD 2 or GD 1 in situ deposits (Adams & Conroy, 2005; Adams et al., 2007b). None of these novel GD A faunas are sufficiently biochronologically sensitive to allow for interpretation relative to the preexisting Gondolin depositional sequence, or suggestive that any deposits at the site are substantially older or younger than the GD 1 or GD 2 in situ deposits (Herries et al., 2006; Adams et al., 2007b). Although no novel bovid tribes or species were recovered, the proportions of bovids within GD A (with few reduncins and oreotragins and a high number of alcelaphin, antilopin, and tragelaphins) stand in stark contrast to the composition of the GD 2 assemblage. This results in a different pattern of bovid size class representation, with a greater representation of larger, bovid class III elements in the GD A sample that contrasts the dominance of smaller class I and II elements in the GD 2 sample (Adams, 2010). The McIntosh evenness statistic value for GD A (0.90) is very high relative to the other Gondolin (GD 1: 0.79, GD 2: 0.60) and comparative fossil and modern karstic assemblages (Adams & Rovinsky, in press; Table 1), but the high taxonomic diversity reflected in the statistic value reflects mostly single individuals in each group. The GD A sample carnivore-ungulate ratio (9.1%) is nearly double that of the larger GD 2 assemblage (4.85%; Adams, 2010), although it is still low relative to the other comparative South African assemblages (Adams & Rovinsky, in press; Table 2). Simultaneously, while the greater taxonomic diversity of the GD A sample relative to other Gondolin deposits like GD 2 is based on a substantially smaller sample size (NISP: 15,246 vs. 95,549 individual specimens from GD 2; Adams, 2010), the proportion of identifiable cranial and postcranial elements from both assemblages is remarkably similar (15.03% for GD A, 17.24% for GD 2; Adams, 2010).

Although basic taphonomic data has been presented as part of the primary description and analysis of the assemblage, just like the taxonomic data little of this can be placed into a readily interpreted context. The internment of specimens with carnivore, porcupine and small rodent damaged remains supports the involvement of at least the former two agents in primary accumulation of at least those modified GD A specimens in the original deposits. The extent and scope of allocthonous component cannot be established, although carnivore- and porcupine-mediated accumulation is consistent with the taphonomic histories of the GD 2 (Adams, 2010) and GD 1 (Adams et al., 2007b) deposits. That said, it is important to reinforce that GD 2 (as a felid-mediated, direct accumulation with rapid sedimentation) and GD 1 (as a hydrologically mediated, likely heavily time averaged and postdepositionally shaped assemblage) may share this basic inclusion of biotically modified elements but have fundamentally different taphonomic histories; such that a finding of a carnivore or porcupine-mediated allocthonous component within GD A is neither surprising (for the site) nor particularly diagnostic (between known deposits). What is worth underscoring is that there is no evidence within the GD A sample for the involvement of any *unique* biotic accumulators relative to the data from the GD 1 or GD 2 assemblages, such as hominins or hyaenids; despite both groups being invoked as accumulators at other South African karstic deposits (Brain, 1981; Adams, 2010). Even though initially noted similarities between the survivorship of total bovid elements in GD A sample and the inverse carnivore consumption sequence (Blumenschine, 1988) could be used to support more substantial carnivore involvement in forming one or more of the original assemblages aggregated within the Dump, this can equally be seen as an artefact of the high numbers of recovered mandibles. When the mandibular %MAU value is removed from the sequence, the %MAU values for the other bovid elements are essentially equivalent and show no trend relative to the consumption sequence.

Other element survivorship assessments (such as utility-indices, density-mediated attrition or hydrological sorting) are equally undiagnostic and do not provide clear evidence for strong biotic or abiotic influences on the composition of the sample. Weathering (i.e. environmental exposure via visual assessment of cortical exfoliation) is a complex feature to interpret for specimens originally interred within karstic deposits; particularly for a sample derived from multiple karstic deposits of unknown history, including at least one phase of natural decalcification underlying the sifted subsample (see Discussion in Adams & Rovinsky, in press). Comparisons of the simple overall size category data indicate no significant differences between any of the major sample subdivisions in the overall length of the fossil specimens recovered. Simultaneously, there is no strong trend in measures of fragmentation (completeness index, NISP:MNE ratio, fracture features) as to the timing of the primary (or secondary) comminution of the assemblage. In only one fracture feature (fracture outline) were there no significant differences with the outgroup comparative assemblages, but this ultimately reflects shared patterns with assemblages broken both while fresh and while dry (Villa & Mahieu, 1991). This lack of a clear pattern follows logically from the long and varied post-depositional history of the deposits (including initial excavation, reduction, and processing) as well as its heterogeneous composition. Similarly, the CI values for bovid carpals and tarsals from GD A were similar to those from the GD 2 assemblage (Adams, 2010), and fell between values reported by Marean (1991) or were equivalent to the values from the GvJm46 ‘post-depositionally modified’ assemblage. This supports the interpretation that there has likely been some degree of post-depositional fragmentation of remains in the assemblage, but how much can be attributed to events occurring prior to initial excavation and internment in the Dump is unknown.

Ultimately, because of the limitations repeatedly noted in this study, the faunal and taphonomic analyses of the GD A provides constrained data for comparisons to the two previously studied Gondolin assemblages and other South African palaeokarstic faunal samples. However, these limitations can be viewed as effectively highlighting the ultimate heuristic value of the GD A assemblage: namely that the only reason for *not* treating the sample as appropriate for comparative study comes from our a priori knowledge of the heterogeneous composition of the fossil specimens in the sample. As discussed above, the artificial aggregation of ex situ materials that contributed to the GD A assemblage presented here mirrors the collection methods used to form many of the historically developed South African palaeokarstic faunal assemblages. In the case of the GD A assemblage described here we simply know more of the specifics about how the ex situ sample was generated to establish reasonable caveats for interpreting the fauna or relying on the sample to form taphonomic or palaeoecologic conclusions about the site. In contrast to GD A, there is scant independent information to verify that early attempts to sort ex situ-derived fossils into units (e.g. ‘Members’) at other South African palaeokarstic sites using gross calcified sediment characteristics led to more accurate associations between blocks or fossil specimens; or have not resulted in substantial artificial time- or spatial-averaging of fossil remains that distort downstream analysis of taphonomy, palaeoecology, or evolutionary events within lineages. Equally, aggregating historically provenanced ex situ fossil specimens with those from subsequent controlled in situ excavations cannot negate this impact and may only provide false assurance that such sampling bias affects have been mitigated because ‘known provenance’ specimens are included in analysis. Furthermore, as our understanding of karstic depositional processes have expanded in the past decades so have our definitions of specific South African site depositional units, which raises questions as to whether ex situ specimens that were allocated based on past definitions of a ‘Member’ can be confidently transferred into these newly defined depositional units (particularly where the original matrix or contextual data can no longer be evaluated). In sum, the justifiable caveats placed on this description and study of the GD A assemblage reflect the level of caution and a far more critical approach to interpretation that is warranted—yet commonly absent—in faunal, taphonomic, and palaeoecologic analyses of the palaeokarst-derived South African faunal record derived solely or in part from ex situ sampling.

## Conclusion

Given all these limitations and caveats applying traditional faunal and taphonomic comparative methods to the GD A faunal assemblage, what reasonable conclusions can be drawn about this fossil sample ‘associated’ with the only two hominin specimens recovered from Gondolin? Fundamentally, the recovery of the hominins themselves and the additional primates, small carnivores and giraffe from Dump A described here represents a primary difference in species presence to the extensively sampled GD 2 and GD 1 faunal assemblages (Adams & Conroy, 2005; Adams et al., 2007b; Adams, 2010). As unique species occurrences for the site relative to both the described in situ deposits, these GD A fossil specimens prompt several alternative interpretations as to their origins within the Gondolin system that are not mutually exclusive.

One potential interpretation is that even through prior sampling of the GD 1 and GD 2 in situ deposits did not recover these primate, carnivore, and ungulate taxa, this reflects sampling (extent and/or strategy) of these two fossil-bearing deposits and not actual presence or absence within one or both of these depositional phases. I have described the sampling methodology and taphonomy of the GD 1 deposits in Adams et al. (2007b) as well as in my unpublished thesis (Adams, 2006). The majority of the sample was derived from naturally decalcified sediments, and the strong hydrological sorting that mediated fossil accumulation and progressive natural decalcification within the sampled GD 1 deposits could have impacted the preservation and recovery of certain taxa. Simultaneously, sampling of the GD 1 deposits ceased because of safety concerns and not because the fossil beds had become sterile. The acetic-acid processing of the GD 2 excavated calcified sediments occurred within a controlled lab setting and captured a substantial volume of delicate and rarely encountered remains. As I discussed fully elsewhere, this makes it unlikely that taxa (particularly hominins or primates) were missed or lost during sampling or processing (Adams, 2006, 2010). As is the case with GD 1, the densely fossiliferous GD 2 deposit matrix was not completely removed during initial excavations. While this may mean there is potential for novel discoveries within the remnant it is only a thin veneer relative to the 2–3 m^3^ that contributed to the processed GD 2 sample, and the exposed remnant surface consists almost entirely of freshly fractured fossil specimens (reflecting that parts of the remnant have in fact already been recorded in the described GD 2 assemblage). Ultimately, application of rare element sampling methods (Trueman et al., 2005) could provide a direct test of the hypothesis that the GD A hominins are derived from either one of these well-characterised in situ deposits, establishing their provenance and context within the depositional history of the site.

A second interpretation is that there are depositional phases within the Gondolin karstic system that contributed to the sampled Dump A fossil materials that have yet to be identified or sampled. The extent of the original fossil deposits within the Gondolin karstic system has not necessarily been fully mapped given that the central portion of the site is dominated by miner’s rubble (and therefore potentially obscuring collapsed roof/wall/floor deposits), and much of the western and northern margins of the mined area have destabilized and collapsed (Menter et al., 1999; Herries et al., 2006; Adams, 2006; Adams et al., 2007b). Resolving this question would require further spelunking coupled with substantial investment in moving overburden and dumpsite materials, potentially coupled with remote sensing technology (Herries et al., 2018).

Finally, the occurrence of the novel taxa in the GD A sample (relative to GD 1 and GD 2) within the ex situ deposits at Gondolin reflects their original deposition within parts of the known Gondolin in situ deposits (whether fossil-bearing or sterile) but not the remnants that could be palaeontologically assessed; or were derived from deposits that existed in the palaeokarstic system but left no remnants after mining. The substantial mining disturbance means that neither the original spatial extent nor fossil composition of the GD 1, GD 2, and GD 3 calcified sediments can be established. Equally, the substantial mining of the Gondolin karstic system could have completely eliminated any number of unique fossil-bearing deposits unrelated to the observable remnants of the modern cave system. No parts of this interpretation are testable, making this perhaps the least satisfying possibility for explaining the occurrence of hominins and unique taxa within the Dump A deposits, and providing less direct positive encouragement for further palaeoanthropologically focused exploration at Gondolin.

## Supplemental Information

10.7717/peerj.5393/supp-1 Supplemental Information 1 Table S1. Catalogue of the Gondolin GD A macromammalian craniodental specimens.

10.7717/peerj.5393/supp-2 Supplemental Information 2 Table S2. Catalogue of GDA specimens: identifiable postcranial specimens and indeterminate specimens.

10.7717/peerj.5393/supp-3 Supplemental Information 3 Table S3. Comparative alcelaphin dental measurements. Measurements for the GD A indeterminate alcelaphins and comparative modern and fossil specimens presented in Figs. 5A–5C. Comparative data from Vrba (1976).

10.7717/peerj.5393/supp-4 Supplemental Information 4 Table S4. Comparative antilopin dental measurements. Measurements for the GD A *Antidorcas* sp. and comparative modern and fossil specimens presented in Fig. 6C. Comparative data from Vrba (1976) and Harris (1991).

## Additional Information and Declarations

### Data Availability

The following information was supplied regarding data availability: All fossil specimens described here both in the article and Supplemental Information are deposited and accessible through the Evolutionary Studies Institute, University of the Witwatersrand, Republic of South Africa.
