# Supplementary material for: Fossil mammals from the Gondolin Dump A ex situ hominin deposits, South Africa
Source: PeerJ. 2018 Aug 6;6:e5393. doi: 10.7717/peerj.5393 (PMC6084286; doi:10.7717/peerj.5393)
Supplement: Supplemental Information 1 [file peerj-06-5393-s001.pdf]

Supplemental Table S1. Catalogue of the Gondolin GD A macromammalian craniodental specimens

Taxon	Specimen	Side	Description
Primates			
Hominini			
Paranthropus			
robustus	GA 2	L	Isolated complete m2 with crown and roots
Hominini indet.	GA 1	L	Isolated m1 or m2 fragment
Primate indet.	GA 165		Indeterminate maxillary molar lingual tooth root
	GA 198		Indeterminate molar tooth root possibly from a mandibular molar
Cetartiodactyla			
Bovidae			
Alcelaphini			
Medium sized			
(Class II-III)	GA 31		Isolated complete upper molar central cavity
	GA 32	R	Lingual enamel surface of a m3 with light occlusal wear
	GA 34		Partial upper molar crown with no occlusal surface or roots preserved
	GA 35	L	Mandible portion with a near complete lower molar (m2?)
	GA 38	R	Complete p4 with heavy occlusal wear
	GA 41	R	Mandible fragment with a partial p4 and complete m1-m3 with moderate occlusal wear
	GA 42	R	Complete indeterminate lower premolar with moderate occlusal wear
	GA 43	L	Near complete M3 with damage to the lingual enamel surfaces and light/moderate occlusal wear
	GA 44	R	Complete unworn m3 with light enamel damage
	GA 46	L	Complete m3 with moderate occlusal wear
	GA 75	L	Complete indeterminate lower molar crown (m1 or m2) with moderate occlusal wear
	GA 76	R	Mostly complete lower molar (m1 or m2) with moderate occlusal wear
	GA 78		Isolated lower molar crown (m1 or m2) with moderate occlusal wear
	GA 79		Isolated lower molar crown (m1 or m2) with damage to the occlusal surface

Taxon	Specimen	Side	Description
Medium sized cont.	GA 80	R	Isolated lower molar crown (m1?) with moderate/heavy occlusal wear
	GA 81	L	Near complete lower molar crown (m1 or m2) with moderate occlusal wear
	GA 83	L	Near complete m3 with damage to the mesial aspect and moderate occlusal wear
	GA 95	R	Complete M2 crown with moderate occlusal wear
	GA 146	R	Near complete lower molar crown (m1 or m2) with light/moderate occlusal wear
	GA 149	R	Near complete m3 with moderate occlusal wear
	GA 151		Portion of an indeterminate upper molar
	GA 190	R	Near complete lower molar (m1?) with heavy occlusal wear
	GA 191		Isolated indeterminate lower molar central cavity
	GA 208		Near complete occlusal surface for an indeterminate lower molar (m1 or m2)
	GA 227	R	Mandible portion with an indeterminate lower molar (m1?) with moderate occlusal wear
	GA 229	R	Complete P3 crown with moderate occlusal wear
	GA 230		Near complete lower molar (m1?) with light occlusal wear
	GA 233		Partial indeterminate lower molar occlusal surface and lacking most of the crown
	GA 239	R	Near complete upper molar crown with moderate occlusal wear
	GA 241	L	Near complete upper molar crown with light/moderate occlusal wear
	GA 244	L	Near complete M3 with moderate occlusal wear
	GA 254	L	Near complete upper molar crown with moderate occlusal wear
	GA 255	R	Partial indeterminate upper molar with light/moderate occlusal wear
	GA 260		Central cavity from an indeterminate upper molar (m2?) with moderate occlusal wear
	GA 261	R	Near complete indeterminate lower molar (m1 or m2) with moderate occlusal wear
	GA 262		Partial indeterminate lower molar occlusal surface
	GA 263	R	Near complete P4 crown with light occlusal wear
	GA 264	R	Near complete upper molar with moderate occlusal wear
	GA 266	R	Near complete upper premolar (P3?) crown with moderate occlusal wear
	GA 290	R	Complete m3 crown with moderate occlusal wear
	GA 293	L	Near complete lower molar (m1 or m2) with moderate occlusal wear (assoc. with GA 290)
	GA 841		Portion of an indeterminate upper molar central cavity
	GA 842	R	Partial upper premolar crown and root with moderate occlusal wear

Taxon	Specimen	Side	Description
Medium sized cont.	GA 843	R	Complete m3 crown with moderate/heavy occlusal wear
	GA 844	L	Partial upper molar crown lacking the occlusal surface
	GA 856	R	Near complete M3 crown with moderate occlusal wear
	GA 858	R	Near complete indeterminate upper molar (m1 or m2) with light/moderate occlusal wear
	GA 859		Small portion of an indeterminate upper molar lingual enamel surface
	GA 880		Partial indeterminate lower molar crown
	GA 1371	L	Complete m3 crown with heavy occlusal wear
	GA 1380	L	Partial crown from an indeterminate lower molar
	GA 1382	L	Partial crown from an indeterminate upper premolar lacking the occlusal surface
	GA 1383	R	Poorly preserved upper molar (M3?) without the occlusal surface
	GA 1408		Partial upper molar central cavity
	GA 1411		Partial lower molar central cavity
	GA 1412		Partial upper molar central cavity
	GA 1413		Complete upper molar central cavity
	GA 1415		Complete lower molar central cavity
	GA 1416		Isolated indeterminate central cavity
	GA 1417		Complete upper molar central cavity
	GA 1418		Complete lower molar central cavity
	GA 1419		Complete upper molar central cavity
	GA 1420		Complete lower molar central cavity
	GA 1421		Complete lower molar central cavity
	GA 1454	R	Partial m3 crown with moderate occlusal wear
	GA 1499		Small portion of the unworn crown of an indeterminate lower molar
	GA 1726	R	Complete m3 crown with moderate/heavy occlusal wear
	GA 1951	R	Near complete lower molar crown (m1 or m2) with light occlusal wear
	GA 1964	L	Complete p4 with light occlusal wear
	GA 1976		Partial indeterminate lower molar (m1 or m2) with moderate occlusal wear
	GA 2003	R	Mandible portion with roots of the p2, p3 deciduous p4 and a partial m1 crown
	GA 2004	L	Complete deciduous p4 crown with near complete roots and moderate occlusal wear
	GA 2029	R	Partial mandible with a partial deciduous p4 with moderate occlusal wear

Taxon	Specimen	Side	Description
Medium sized cont.	GA 2100	R	Partial P3 with moderate occlusal wear
	GA 2165	R	Partial mandibular ramus with a near complete deciduous p4 with moderate occlusal wear
Large sized (Class III)	GA 91	L	Near complete p4 crown and roots
	GA 243	L	Complete P4 crown with moderate/heavy occlusal wear and part of the lingual root
	GA 337	L	Near complete unworn P4 crown with light damage to the crown
	GA 1489	L	Complete M3 crown and roots with moderate occlusal wear
	GA 1568	L	Complete lower molar (m1 or m2) with moderate occlusal wear
	GA 1942	R	Complete upper molar (M3?) crown with damage to the occlusal surface
Antilopini *Antidorcas* sp.	GA 36	R	Partial crown of an upper molar (M2?) with light occlusal wear
	GA 242	L	Complete M3 crown with a lightly worn and damaged occlusal surface
	GA 248	L	Near complete upper molar (M3?) with light occlusal wear
	GA 251	R	Complete m2 crown with moderate occlusal wear
	GA 253	R	Complete M3 crown with light occlusal wear
Hippotragini *Hippotragus equinus*	GA 2161	L	Near complete unworn P4 crown
	GA 2173	R	Complete p2 with light/moderate occlusal wear
	GA 2174	L	Partial paraconid from a lower premolar (p3?) associated with GA 2161, 2173 and 2175
	GA 2175	L	Complete p3 crown with moderate occlusal wear
Oreotragini *Oreotragus* sp.	GA 209	L	Maxilla portion with P2, P3 (possible deciduous), deciduous P4, M1 and M2 (in eruption)
	GA 234	L	Complete M1 crown with extremely heavy occlusal wear
	GA 1960	L	Partial P2 crown with moderate occlusal wear
	GA 2035	L	Mandible portion complete p3-m1 and the alveolus for the m2

Taxon	Specimen	Side	Description
Reduncini			
Redunca sp.	GA 8	L	Complete unworn P2 crown and roots
	GA 20	R	Partial mandible with a partial m1, complete m2 and partial m3 with moderate occlusal wear
	GA 21	L	Partial mandible with complete m1-m3 with superficial occlusal wear to the m3
	GA 45	R	Complete unworn p2 crown
	GA 147	L	Partial mandible with complete m1-m3 with superficial occlusal wear to the m3
	GA 170	R	Complete P3 crown in moderate occlusal wear
	GA 175	L	Nearly complete M1 with damage to the lingual enamel surfaces
	GA 236	R	Complete P3 crown with light/moderate occlusal wear
	GA 237	R	Complete P3 crown and roots with very superficial occlusal wear
	GA 252	R	Complete M3 crown with light occlusal wear
	GA 311	R	Complete P4 crown with partial roots and moderate occlusal wear
	GA 312	L	Complete P4 crown with heavy occlusal wear
	GA 313	R	Complete P4 crown with moderate occlusal wear
	GA 868	R	Isolated partial lower molar with light occlusal wear
	GA 1028	L	Near complete deciduous P3 crown with superficial occlusal wear
	GA 2096	L	Partial lower molar (m1?) with damage to the lingual enamel surfaces
Tragelaphini			
Tragelaphus oryx	GA 9	R	Isolated p2 crown without occlusal wear or roots
	GA 39	R	Damaged lower molar with near complete roots and moderate occlusal wear
	GA 1029	R	Isolated nearly complete M3 with moderate/heavy occlusal wear and partial roots
Tragelaphus strepsiceros	GA 294	L	Small portion of the mandibular ramus with the deciduous p4 and part of the m1 preserved
Tragelaphini indet.	GA 13	R	Distal portion of a heavily worn indeterminate lower molar with a partial root
	GA 238	L	Near complete upper molar crown with light/moderate occlusal wear
	GA 245	L	Mandible fragment with a partial m2 and m3 with moderate occlusal wear

Taxon	Specimen	Side	Description
Tragelaphini indet. continued	GA 247	R	Probable mandible fragment with small parts of two molars with moderate occlusal wear
	GA 314		Partial cusp and central cavity of an upper molar
	GA 348	L	Partial isolated upper molar
	GA 846	R	Near complete m3 with light occlusal wear and a small portion of the root adhering
	GA 1369		Isolated lower molar cusp with part of a central cavity and light occlusal wear
	GA 1488	R	Near complete m3 crown with part of the mesial root and moderate/heavy occlusal wear
	GA 2102		Small portion of a buccal cusp of an indeterminate lower molar
Giraffidae			
Giraffa sp.	GA 308	L	Isolated nearly complete deciduous P3 with light occlusal wear and damage to the distal aspect
Suidae			
Suidae indet.	GA 164		Small, heavily worn occlusal surface with an isolated thick enamel 'island' and thick cementum
	GA 2123		Isolated unworn terminal pillar with thick enamel
	GA 2141		Isolated portion of a occluded terminal pillar with thick enamel
	GA 2156		Isolated enamel fragment
Carnivora			
Felidae indet.	GA 318	R	Isolated buccal portion of P4 with the paraconid root (possibly associated with GA 319)
	GA 319	R	Isolated portion of a P3 possibly associated with the GA 318 specimen
Herpestidae indet.	GA 1482	R	Isolated near complete p4 with some damage to the cusps
	GA 1483	L	Mandible portion with parts of the p4 and m1 preserved
	GA 1484	R	Near complete mandible with portions of the p2, p3 and m1 preserved
Carnivore indet.	GA 2121		Indeterminate (possible felid) premolar fragment with occlusal wear
	GA 2129		Isolated canine crown from medium sized carnivore

Taxon	Specimen	Side	Description
Perissodactyla			
Equidae			
Equus capensis	GA 93	L	Nearly complete deciduous P2 with heavy occlusal wear and resorbed roots
	GA 307	L	Lingual aspect of an indeterminate upper premolar or molar in wear
	GA 2169	R	Isolated complete crown of an indeterminate upper premolar or molar in moderate wear
	GA 2026	R	Near complete unworn deciduous upper premolar crown
Equus sp.	GA 92	R	Portion of the buccal aspects of a p2 without roots
	GA 156	L	Mandible fragment with portions of two extremely occluded premolars/molars (m1/m2?)
	GA 303	R	Complete isolated i1 with moderate occlusal and interstitial wear
	GA 309		Isolated upper incisor root (possibly from a right I2)
	GA 1780	R	Isolated crown of an i1 or i2 without root and heavy interstitial wear on the mesial surface
	GA 1858	L	Isolated buccal aspect of the mesial occlusal surface from a lower premolar or molar
	GA 2126		Small portion of the central cavity of an indeterminate upper premolar or molar
Hyracoidea			
Procaviidae			
Procavia sp.	GA 1342	R	Complete upper i1 with morphology consistent with a female
	GA 1943	R	Complete unworn crown of a lower permanent p2 or deciduous p2 without roots
	GA 1990	R	Poorly preserved P3 or P4 with heavy occlusal wear
	GA 2160	L	Near complete crown and root of an i2
Rodentia			
Hystrix africaeaustralis	GA 6	L	Complete crown of an indeterminate upper molar with light occlusal wear
Hystrix makapanensis	GA 7	L	Isolated p4 with moderate occlusal wear (possibly from the same individual as GA 50)
	GA 11	R	Isolated complete M1 with a moderately worn occlusal surface
	GA 50	L	Complete M2 or M3 crown with moderate occlusal wear

Taxon	Specimen	Side	Description
Hystrix makapanensis cont.	GA 839	L	Portion of the mandibular ramus with a near complete p4
Hystricidae indet.	GA 12		Partial occlusal surface of an indeterminate premolar or molar (possibly *H. makapanensis*)
	GA 77		Portion of alveolus and incisor enamel of a probable right upper incisor
	GA 320		Isolated small portion of an indeterminate incisor
	GA 1915		Isolated small portion of an indeterminate incisor
Lagomorpha			
Leporidae indet.	GA 2189	L	Small mandibular ramus portion with a complete p4 and roots for the m1
