# Supplementary material for: Fossil mammals from the Gondolin Dump A ex situ hominin deposits, South Africa
Source: PeerJ. 2018 Aug 6;6:e5393. doi: 10.7717/peerj.5393 (PMC6084286; doi:10.7717/peerj.5393)
Supplement: Supplemental Information 2 [file peerj-06-5393-s002.pdf]

Supplemental Table S2. Catalogue of GDA Specimens: Identifiable Postcranial Specimens and Indeterminate Specimens

GA #	Class or Order	Family	Bovid Size Class	Element	Part or Portion	Side	Overall Size (cm)	Specimen Count
3	Mammalia	Indeterminate		Indeterminate	Fragment		2	
4	Mammalia	Indeterminate		Cranial	Mandible		3	
5	Cetartiodactyla	Bovidae		Cranial	Mandibular second premolar	Right	1	
10	Cetartiodactyla	Bovidae		Cranial	Enamel Fragment		0	
15	Mammalia	Indeterminate		Cranial	Enamel Fragment		0	
16	Mammalia	Indeterminate		Cranial	Fragment		2	
17	Mammalia	Indeterminate		Indeterminate	Fragment		2	
18	Mammalia	Indeterminate		Indeterminate	Fragment		3	
19	Mammalia	Indeterminate		Indeterminate	Fragment		2	
22	Cetartiodactyla	Bovidae	II	Humerus	Distal epiphysis	Right	5	
23	Cetartiodactyla	Bovidae	II	Calcaneus	Proximal epiphysis	Right	5	
26	Cetartiodactyla	Bovidae	I	Calcaneus	Proximal epiphysis	Right	1	
27	Mammalia	Indeterminate		Cranial	Enamel Fragment		2	
28	Cetartiodactyla	Bovidae	II	Metapodial	Diaphysis		2	
29	Mammalia	Indeterminate		Indeterminate	Fragment		5	
30	Mammalia	Indeterminate		Indeterminate	Fragment		7	
33	Cetartiodactyla	Bovidae	II	Cranial	Mandibular second incisor	Right	2	
37	Cetartiodactyla	Bovidae		Cranial	Enamel Fragment		1	
40	Cetartiodactyla	Bovidae		Cranial	Mandibular third molar		3	
47	Mammalia	Indeterminate		Cranial	Enamel Fragment		1	
48	Mammalia	Indeterminate		Indeterminate	Fragment		6	
51	Cetartiodactyla	Bovidae	II	Proximal Phalanx	Proximal epiphysis		1	
52	Cetartiodactyla	Bovidae	II	Metapodial	Distal metaphysis		3	
54	Mammalia	Indeterminate		Indeterminate	Fragment		5	
56	Cetartiodactyla	Bovidae	II	Metapodial	Diaphysis		8	
57	Mammalia	Indeterminate		Vertebra	Caudal		2	
58	Cetartiodactyla	Bovidae	III	Proximal Phalanx	Distal epiphysis		2	
60	Cetartiodactyla	Bovidae	III	Metapodial	Distal epiphysis		3	
61	Mammalia	Indeterminate		Indeterminate	Fragment		3	
62	Cetartiodactyla	Bovidae	II	Distal Phalanx	Complete		2	
63	Mammalia	Indeterminate		Indeterminate	Fragment		1	
64	Cetartiodactyla	Bovidae	II	Humerus	Distal epiphysis	Right	3	
65	Mammalia	Indeterminate		Metatarsal	Proximal epiphysis		1	
66	Mammalia	Indeterminate		Metapodial	Distal epiphysis		0	
67	Mammalia	Indeterminate		Indeterminate	Fragment		1	
68	Cetartiodactyla	Bovidae	II	Ulna	Proximal epiphysis	Left	3	
70	Cetartiodactyla	Bovidae	I	Intermediate Phalanx	Complete		1	
71	Cetartiodactyla	Bovidae	I	Proximal Phalanx	Proximal epiphysis		1	
72	Mammalia	Indeterminate		Indeterminate	Fragment		3	
73	Cetartiodactyla	Bovidae	III	Proximal Phalanx	Distal epiphysis		3	
74	Mammalia	Indeterminate		Indeterminate	Fragment		10	
84	Cetartiodactyla	Bovidae	II	Radius	Distal epiphysis	Right	4	
85	Cetartiodactyla	Bovidae	II	Scapula	Glenoid	Left	7	
87	Mammalia	Indeterminate		Indeterminate	Fragment		2	
88	Mammalia	Indeterminate		Diaphysis	Fragment		20	
89	Cetartiodactyla	Bovidae	II	Metatarsal	Diaphysis		3	
90	Cetartiodactyla	Bovidae	II	Radius	Diaphysis		3	
94	Mammalia	Indeterminate		Indeterminate	Fragment		3	
100	Mammalia	Indeterminate		Cranial	Enamel Fragment		1	
101	Mammalia	Indeterminate		Indeterminate	Fragment		1	
102	Mammalia	Indeterminate		Indeterminate	Fragment		2	
103	Mammalia	Indeterminate		Indeterminate	Fragment		1	
104	Mammalia	Indeterminate		Indeterminate	Fragment		8	
105	Mammalia	Indeterminate		Indeterminate	Fragment		2	
106	Mammalia	Indeterminate		Indeterminate	Fragment		6	
107	Mammalia	Indeterminate		Indeterminate	Fragment		4	
108	Mammalia	Indeterminate		Indeterminate	Fragment		2	
109	Mammalia	Indeterminate		Indeterminate	Fragment		3	
110	Mammalia	Indeterminate		Indeterminate	Fragment		1	
111	Mammalia	Indeterminate		Indeterminate	Fragment		1	
112	Cetartiodactyla	Bovidae	II	Cranial	Enamel Fragment		2	
113	Mammalia	Indeterminate		Indeterminate	Fragment		4	
114	Mammalia	Indeterminate		Indeterminate	Fragment		3	
115	Mammalia	Indeterminate		Indeterminate	Fragment		2	
116	Mammalia	Indeterminate		Indeterminate	Fragment		1	

Supplemental Table S2. Catalogue of GDA Specimens: Identifiable Postcranial Specimens and Indeterminate Specimens

GA #	Class or Order	Family	Bovid Size Class	Element	Part or Portion	Side	Overall Size (cm)	Specimen Count
117	Mammalia	Indeterminate		Indeterminate	Fragment		0	N=563
118	Cetartiodactyla	Bovidae	II	Astragalus	Distal epiphysis	Right	2	
119	Mammalia	Indeterminate		Cranial	Fragment		1	
121	Mammalia	Indeterminate		Cranial	Enamel Fragment		2	
122	Mammalia	Indeterminate		Indeterminate	Fragment		8	
123	Mammalia	Indeterminate		Indeterminate	Fragment		3	
124	Cetartiodactyla	Bovidae	II	Cranial	Temporal	Left	1	
125	Mammalia	Indeterminate		Indeterminate	Fragment		1	
126	Mammalia	Indeterminate		Rib	Diaphysis		3	
127	Mammalia	Indeterminate		Indeterminate	Fragment		2	
129	Cetartiodactyla	Bovidae	III	Metacarpal	Proximal epiphysis	Right	5	
130	Mammalia	Indeterminate		Indeterminate	Fragment		4	
131	Mammalia	Indeterminate		Indeterminate	Fragment		1	
132	Mammalia	Indeterminate		Indeterminate	Fragment		8	
133	Mammalia	Indeterminate		Indeterminate	Fragment		0	N=3
134	Mammalia	Indeterminate		Indeterminate	Fragment		7	
135	Mammalia	Indeterminate		Indeterminate	Fragment		6	
137	Cetartiodactyla	Bovidae		Cranial	Enamel Fragment		0	N=5
144	Mammalia	Indeterminate		Pelvis	Fragment			
145	Mammalia	Indeterminate		Vertebra	Fragment		0	
150	Cetartiodactyla	Bovidae		Cranial	Mandibular premolar	Right	1	
153	Mammalia	Indeterminate		Indeterminate	Fragment		1	
154	Mammalia	Indeterminate		Cranial	Enamel Fragment		1	
155	Mammalia	Indeterminate		Indeterminate	Fragment		0	N=61
157	Cetartiodactyla	Bovidae	III	Proximal Phalanx	Distal epiphysis		3	
159	Mammalia	Indeterminate		Indeterminate	Fragment		3	N=3
160	Mammalia	Indeterminate		Indeterminate	Fragment		2	
161	Mammalia	Indeterminate		Indeterminate	Fragment		3	
162	Cetartiodactyla	Bovidae	II	Femur	Distal epiphysis	Left	8	
163	Mammalia	Indeterminate		Indeterminate	Fragment		2	
166	Mammalia	Indeterminate		Vertebra	Lumbar		2	
167	Mammalia	Indeterminate		Rib	Distal epiphysis		1	
168	Cetartiodactyla	Bovidae	II	Cranial	Temporal		2	
171	Cetartiodactyla	Bovidae		Cranial	Enamel Fragment		2	
176	Mammalia	Indeterminate		Indeterminate	Fragment		2	
177	Mammalia	Indeterminate		Indeterminate	Fragment		1	
178	Mammalia	Indeterminate		Indeterminate	Fragment		0	N=553
179	Mammalia	Indeterminate		Indeterminate	Fragment		5	
181	Mammalia	Indeterminate		Indeterminate	Fragment		7	
182	Mammalia	Indeterminate		Indeterminate	Fragment		5	
183	Mammalia	Indeterminate		Indeterminate	Fragment		4	
184	Mammalia	Indeterminate		Indeterminate	Fragment		2	
185	Mammalia	Indeterminate		Indeterminate	Fragment		4	
186	Mammalia	Indeterminate		Cranial	Enamel Fragment		0	N=5
187	Mammalia	Indeterminate		Cranial	Enamel Fragment		1	
188	Cetartiodactyla	Bovidae		Cranial	Enamel Fragment		2	
189	Cetartiodactyla	Bovidae	I	Femur	Distal epiphysis	Left	2	
192	Mammalia	Indeterminate		Rib	Diaphysis		2	
194	Mammalia	Indeterminate		Indeterminate	Fragment		1	
196	Cetartiodactyla	Bovidae		Cranial	Maxillary molar		2	
197	Cetartiodactyla	Bovidae		Cranial	Enamel Fragment		1	
199	Cetartiodactyla	Bovidae		Cranial	Maxilla	Right	3	
201	Cetartiodactyla	Bovidae	II	Cranial	Mandibular second incisor	Left	1	
202	Cetartiodactyla	Bovidae	II	Metacarpal	Distal epiphysis		4	
204	Cetartiodactyla	Bovidae	II	Scapula	Glenoid	Right	7	
205	Cetartiodactyla	Bovidae	II	Intermediate Phalanx	Complete		2	
206	Mammalia	Indeterminate		Cranial	Fragment		2	
207	Mammalia	Indeterminate		Indeterminate	Fragment		0	N=7
211	Mammalia	Indeterminate		Cranial	Enamel Fragment		0	N=2
214	Mammalia	Indeterminate		Indeterminate	Fragment		6	
215	Mammalia	Indeterminate		Cranial	Enamel Fragment		0	N=4
217	Cetartiodactyla	Bovidae	II	Carpal	Radial	Right	2	
218	Mammalia	Indeterminate		Cranial	Enamel Fragment		1	
219	Mammalia	Indeterminate		Indeterminate	Fragment		1	
220	Cetartiodactyla	Bovidae	II	Cranial	Temporal	Left	2	

Supplemental Table S2. Catalogue of GDA Specimens: Identifiable Postcranial Specimens and Indeterminate Specimens

GA #	Class or Order	Family	Bovid Size Class	Element	Part or Portion	Side	Overall Size (cm)	Specimen Count
221	Cetartiodactyla	Bovidae	III	Cranial	Temporal		2	
222	Cetartiodactyla	Bovidae	II	Astragalus	Complete	Left	3	
223	Cetartiodactyla	Bovidae	II	Metapodial	Distal epiphysis		2	
224	Cetartiodactyla	Bovidae	II	Calcaneus	Proximal epiphysis	Right	5	
225	Mammalia	Indeterminate		Indeterminate	Fragment		5	
226	Mammalia	Indeterminate		Indeterminate	Fragment		3	
228	Mammalia	Indeterminate		Indeterminate	Fragment		1	
231	Mammalia	Indeterminate		Indeterminate	Fragment		3	
232	Mammalia	Indeterminate		Cranial	Enamel Fragment		0	N=8
235	Cetartiodactyla	Bovidae		Cranial	Maxillary molar		1	
240	Cetartiodactyla	Bovidae		Cranial	Maxillary premolar		1	
246	Cetartiodactyla	Bovidae	III	Cranial	Mandibular third molar	Left	2	
249	Mammalia	Indeterminate		Indeterminate	Fragment		0	N=9
250	Mammalia	Indeterminate		Cranial	Enamel Fragment		0	N=1
256	Cetartiodactyla	Bovidae	II	Vertebra	Axis		5	
257	Cetartiodactyla	Bovidae	II	Vertebra	Atlas		7	
258	Cetartiodactyla	Bovidae	II	Vertebra	Lumbar		4	
265	Cetartiodactyla	Bovidae		Cranial	Maxillary premolar/molar		2	
270	Cetartiodactyla	Bovidae		Cranial	Indeterminate molar		1	
274	Mammalia	Indeterminate		Indeterminate	Fragment		1	
275	Cetartiodactyla	Bovidae		Cranial	Enamel Fragment		0	N=3
279	Mammalia	Indeterminate		Indeterminate	Fragment		1	
280	Mammalia	Indeterminate		Indeterminate	Fragment		2	
281	Mammalia	Indeterminate		Indeterminate	Fragment		1	
282	Mammalia	Indeterminate		Indeterminate	Fragment		0	N=70
283	Cetartiodactyla	Bovidae		Cranial	Enamel Fragment		0	N=2
284	Mammalia	Indeterminate		Indeterminate	Fragment		0	N=27
286	Mammalia	Indeterminate		Indeterminate	Fragment		3	
287	Mammalia	Indeterminate		Indeterminate	Fragment		0	N=213
289	Mammalia	Indeterminate		Indeterminate	Fragment		2	
291	Mammalia	Indeterminate		Cranial	Fragment		2	
292	Cetartiodactyla	Bovidae		Cranial	Enamel Fragment		2	
302	Mammalia	Indeterminate		Cranial	Enamel Fragment		0	N=17
305	Cetartiodactyla	Bovidae		Cranial	Mandible	Left	4	
306	Mammalia	Indeterminate		Cranial	Fragment		2	
310	Mammalia	Indeterminate		Indeterminate	Fragment		1	
316	Mammalia	Indeterminate		Cranial	Enamel Fragment		0	N=2
317	Mammalia	Indeterminate		Indeterminate	Fragment		2	
321	Mammalia	Indeterminate		Cranial	Enamel Fragment		2	
322	Mammalia	Indeterminate		Indeterminate	Fragment		1	
323	Mammalia	Indeterminate		Indeterminate	Fragment		2	
324	Cetartiodactyla	Bovidae	III	Tarsal	First	Left	1	
327	Cetartiodactyla	Bovidae	II	Cranial	Temporal	Left	2	
328	Mammalia	Indeterminate		Indeterminate	Fragment		3	
329	Mammalia	Indeterminate		Indeterminate	Fragment		2	
330	Mammalia	Indeterminate		Indeterminate	Fragment		1	
331	Mammalia	Indeterminate		Indeterminate	Fragment		0	N=537
332	Mammalia	Indeterminate		Cranial	Enamel Fragment		1	
333	Cetartiodactyla	Bovidae	II	Metacarpal	Distal epiphysis		5	
334	Cetartiodactyla	Bovidae	II	Intermediate Phalanx	Complete		3	
335	Cetartiodactyla	Bovidae	II	Intermediate Phalanx	Complete		2	
336	Cetartiodactyla	Bovidae	II	Pelvis	Ilium	Left	8	
339	Mammalia	Indeterminate		Indeterminate	Fragment		4	
340	Mammalia	Indeterminate		Indeterminate	Fragment		2	
341	Mammalia	Indeterminate		Indeterminate	Fragment		1	
342	Mammalia	Indeterminate		Indeterminate	Fragment		0	
343	Cetartiodactyla	Bovidae		Cranial	Enamel Fragment		2	
344	Cetartiodactyla	Bovidae		Cranial	Enamel Fragment		1	
345	Mammalia	Indeterminate		Cranial	Enamel Fragment		0	
346	Mammalia	Indeterminate		Indeterminate	Fragment		6	
347	Mammalia	Indeterminate		Indeterminate	Fragment		6	
400	Mammalia	Indeterminate		Indeterminate	Fragment		2	
401	Mammalia	Indeterminate		Indeterminate	Fragment		2	
402	Mammalia	Indeterminate		Indeterminate	Fragment		2	
403	Mammalia	Indeterminate		Indeterminate	Fragment		1	

Supplemental Table S2. Catalogue of GDA Specimens: Identifiable Postcranial Specimens and Indeterminate Specimens

GA #	Class or Order	Family	Bovid Size Class	Element	Part or Portion	Side	Overall Size (cm)	Specimen Count
404	Mammalia	Indeterminate		Indeterminate	Fragment		1	
405	Mammalia	Indeterminate		Indeterminate	Fragment		3	
406	Mammalia	Indeterminate		Indeterminate	Fragment		3	
407	Mammalia	Indeterminate		Indeterminate	Fragment		3	
408	Mammalia	Indeterminate		Indeterminate	Fragment		3	
409	Mammalia	Indeterminate		Indeterminate	Fragment		1	
410	Mammalia	Indeterminate		Indeterminate	Fragment		1	
411	Mammalia	Indeterminate		Indeterminate	Fragment		1	
412	Mammalia	Indeterminate		Indeterminate	Fragment		1	
413	Mammalia	Indeterminate		Indeterminate	Fragment		1	
414	Mammalia	Indeterminate		Indeterminate	Fragment		1	
415	Mammalia	Indeterminate		Indeterminate	Fragment		1	
416	Mammalia	Indeterminate		Indeterminate	Fragment		1	
417	Mammalia	Indeterminate		Indeterminate	Fragment		1	
418	Mammalia	Indeterminate		Indeterminate	Fragment		2	
419	Mammalia	Indeterminate		Indeterminate	Fragment		2	
420	Mammalia	Indeterminate		Indeterminate	Fragment		2	
421	Mammalia	Indeterminate		Indeterminate	Fragment		2	
422	Mammalia	Indeterminate		Indeterminate	Fragment		1	
423	Mammalia	Indeterminate		Indeterminate	Fragment		1	
424	Mammalia	Indeterminate		Indeterminate	Fragment		1	
426	Mammalia	Indeterminate		Indeterminate	Fragment		3	
427	Mammalia	Indeterminate		Indeterminate	Fragment		2	
428	Mammalia	Indeterminate		Indeterminate	Fragment		2	
429	Mammalia	Indeterminate		Indeterminate	Fragment		2	
430	Mammalia	Indeterminate		Indeterminate	Fragment		2	
431	Mammalia	Indeterminate		Indeterminate	Fragment		2	
432	Mammalia	Indeterminate		Indeterminate	Fragment		2	
433	Mammalia	Indeterminate		Indeterminate	Fragment		2	
434	Mammalia	Indeterminate		Indeterminate	Fragment		7	
435	Mammalia	Indeterminate		Indeterminate	Fragment		2	
436	Mammalia	Indeterminate		Indeterminate	Fragment		2	
437	Mammalia	Indeterminate		Indeterminate	Fragment		2	
438	Mammalia	Indeterminate		Indeterminate	Fragment		2	
439	Mammalia	Indeterminate		Indeterminate	Fragment		1	
440	Mammalia	Indeterminate		Indeterminate	Fragment		1	
441	Mammalia	Indeterminate		Indeterminate	Fragment		1	
442	Mammalia	Indeterminate		Indeterminate	Fragment		1	
443	Mammalia	Indeterminate		Indeterminate	Fragment		1	
444	Mammalia	Indeterminate		Indeterminate	Fragment		1	
445	Mammalia	Indeterminate		Indeterminate	Fragment		1	
446	Mammalia	Indeterminate		Indeterminate	Fragment		1	
447	Mammalia	Indeterminate		Indeterminate	Fragment		0	
448	Mammalia	Indeterminate		Indeterminate	Fragment		1	
449	Mammalia	Indeterminate		Indeterminate	Fragment		1	
450	Mammalia	Indeterminate		Indeterminate	Fragment		1	
451	Mammalia	Indeterminate		Indeterminate	Fragment		1	
452	Mammalia	Indeterminate		Indeterminate	Fragment		1	
453	Mammalia	Indeterminate		Indeterminate	Fragment		1	
454	Mammalia	Indeterminate		Indeterminate	Fragment		1	
455	Mammalia	Indeterminate		Indeterminate	Fragment		1	
456	Mammalia	Indeterminate		Indeterminate	Fragment		1	
457	Mammalia	Indeterminate		Indeterminate	Fragment		1	
458	Mammalia	Indeterminate		Indeterminate	Fragment		1	
459	Mammalia	Indeterminate		Indeterminate	Fragment		1	
460	Mammalia	Indeterminate		Indeterminate	Fragment		1	
461	Mammalia	Indeterminate		Indeterminate	Fragment		1	
462	Mammalia	Indeterminate		Indeterminate	Fragment		1	
463	Mammalia	Indeterminate		Indeterminate	Fragment		1	
464	Mammalia	Indeterminate		Indeterminate	Fragment		1	
465	Mammalia	Indeterminate		Indeterminate	Fragment		1	
466	Mammalia	Indeterminate		Indeterminate	Fragment		1	
467	Mammalia	Indeterminate		Indeterminate	Fragment		1	
468	Mammalia	Indeterminate		Indeterminate	Fragment		1	
469	Mammalia	Indeterminate		Indeterminate	Fragment		1	

Supplemental Table S2. Catalogue of GDA Specimens: Identifiable Postcranial Specimens and Indeterminate Specimens

GA #	Class or Order	Family	Bovid Size Class	Element	Part or Portion	Side	Overall Size (cm)	Specimen Count
470	Mammalia	Indeterminate		Indeterminate	Fragment		1	
471	Mammalia	Indeterminate		Indeterminate	Fragment		1	
472	Mammalia	Indeterminate		Indeterminate	Fragment		1	
473	Mammalia	Indeterminate		Indeterminate	Fragment		1	
474	Mammalia	Indeterminate		Indeterminate	Fragment		1	
475	Mammalia	Indeterminate		Indeterminate	Fragment		1	
476	Mammalia	Indeterminate		Indeterminate	Fragment		1	
477	Mammalia	Indeterminate		Indeterminate	Fragment		1	
478	Mammalia	Indeterminate		Indeterminate	Fragment		1	
479	Mammalia	Indeterminate		Indeterminate	Fragment		1	
480	Mammalia	Indeterminate		Indeterminate	Fragment		1	
481	Mammalia	Indeterminate		Indeterminate	Fragment		1	
482	Mammalia	Indeterminate		Indeterminate	Fragment		1	
483	Mammalia	Indeterminate		Indeterminate	Fragment		1	
484	Mammalia	Indeterminate		Indeterminate	Fragment		1	
485	Mammalia	Indeterminate		Indeterminate	Fragment		1	
486	Mammalia	Indeterminate		Indeterminate	Fragment		1	
487	Mammalia	Indeterminate		Indeterminate	Fragment		1	
488	Mammalia	Indeterminate		Indeterminate	Fragment		1	
489	Mammalia	Indeterminate		Indeterminate	Fragment		1	
490	Mammalia	Indeterminate		Indeterminate	Fragment		1	
491	Mammalia	Indeterminate		Indeterminate	Fragment		1	
492	Mammalia	Indeterminate		Indeterminate	Fragment		1	
493	Mammalia	Indeterminate		Indeterminate	Fragment		1	
494	Mammalia	Indeterminate		Indeterminate	Fragment		1	
495	Mammalia	Indeterminate		Indeterminate	Fragment		1	
496	Mammalia	Indeterminate		Indeterminate	Fragment		1	
497	Mammalia	Indeterminate		Indeterminate	Fragment		1	
498	Mammalia	Indeterminate		Indeterminate	Fragment		1	
499	Mammalia	Indeterminate		Indeterminate	Fragment		1	
500	Mammalia	Indeterminate		Indeterminate	Fragment		1	
501	Mammalia	Indeterminate		Indeterminate	Fragment		1	
502	Mammalia	Indeterminate		Indeterminate	Fragment		1	
503	Mammalia	Indeterminate		Indeterminate	Fragment		1	
504	Mammalia	Indeterminate		Indeterminate	Fragment		1	
505	Mammalia	Indeterminate		Indeterminate	Fragment		1	
506	Mammalia	Indeterminate		Indeterminate	Fragment		1	
507	Mammalia	Indeterminate		Indeterminate	Fragment		1	
508	Mammalia	Indeterminate		Indeterminate	Fragment		1	
509	Mammalia	Indeterminate		Indeterminate	Fragment		1	
510	Mammalia	Indeterminate		Indeterminate	Fragment		1	
511	Mammalia	Indeterminate		Indeterminate	Fragment		1	
512	Mammalia	Indeterminate		Indeterminate	Fragment		1	
513	Mammalia	Indeterminate		Indeterminate	Fragment		1	
514	Mammalia	Indeterminate		Indeterminate	Fragment		1	
515	Mammalia	Indeterminate		Indeterminate	Fragment		1	
516	Mammalia	Indeterminate		Indeterminate	Fragment		1	
517	Mammalia	Indeterminate		Indeterminate	Fragment		1	
518	Mammalia	Indeterminate		Indeterminate	Fragment		1	
519	Mammalia	Indeterminate		Indeterminate	Fragment		1	
520	Mammalia	Indeterminate		Indeterminate	Fragment		1	
521	Mammalia	Indeterminate		Indeterminate	Fragment		1	
522	Mammalia	Indeterminate		Indeterminate	Fragment		1	
523	Mammalia	Indeterminate		Indeterminate	Fragment		1	
524	Mammalia	Indeterminate		Indeterminate	Fragment		1	
525	Mammalia	Indeterminate		Indeterminate	Fragment		1	
526	Mammalia	Indeterminate		Indeterminate	Fragment		1	
527	Mammalia	Indeterminate		Indeterminate	Fragment		1	
528	Mammalia	Indeterminate		Indeterminate	Fragment		1	
529	Mammalia	Indeterminate		Indeterminate	Fragment		1	
530	Mammalia	Indeterminate		Indeterminate	Fragment		1	
531	Mammalia	Indeterminate		Indeterminate	Fragment		1	
532	Mammalia	Indeterminate		Indeterminate	Fragment		1	
533	Mammalia	Indeterminate		Indeterminate	Fragment		1	
534	Mammalia	Indeterminate		Indeterminate	Fragment		1	

Supplemental Table S2. Catalogue of GDA Specimens: Identifiable Postcranial Specimens and Indeterminate Specimens

GA #	Class or Order	Family	Bovid Size Class	Element	Part or Portion	Side	Overall Size (cm)	Specimen Count
535	Mammalia	Indeterminate		Indeterminate	Fragment		1	
536	Mammalia	Indeterminate		Indeterminate	Fragment		1	
537	Mammalia	Indeterminate		Indeterminate	Fragment		1	
538	Mammalia	Indeterminate		Indeterminate	Fragment		1	
539	Mammalia	Indeterminate		Indeterminate	Fragment		1	
540	Mammalia	Indeterminate		Indeterminate	Fragment		1	
541	Mammalia	Indeterminate		Indeterminate	Fragment		1	
542	Mammalia	Indeterminate		Indeterminate	Fragment		1	
543	Mammalia	Indeterminate		Indeterminate	Fragment		1	
544	Mammalia	Indeterminate		Indeterminate	Fragment		1	
545	Mammalia	Indeterminate		Indeterminate	Fragment		1	
546	Mammalia	Indeterminate		Indeterminate	Fragment		1	
547	Mammalia	Indeterminate		Indeterminate	Fragment		1	
548	Mammalia	Indeterminate		Indeterminate	Fragment		1	
549	Mammalia	Indeterminate		Indeterminate	Fragment		1	
550	Mammalia	Indeterminate		Indeterminate	Fragment		1	
551	Mammalia	Indeterminate		Indeterminate	Fragment		1	
552	Mammalia	Indeterminate		Indeterminate	Fragment		1	
553	Mammalia	Indeterminate		Indeterminate	Fragment		1	
554	Mammalia	Indeterminate		Indeterminate	Fragment		1	
555	Mammalia	Indeterminate		Indeterminate	Fragment		1	
556	Mammalia	Indeterminate		Indeterminate	Fragment		1	
557	Mammalia	Indeterminate		Indeterminate	Fragment		1	
558	Mammalia	Indeterminate		Indeterminate	Fragment		1	
559	Mammalia	Indeterminate		Indeterminate	Fragment		1	
560	Mammalia	Indeterminate		Indeterminate	Fragment		1	
561	Mammalia	Indeterminate		Indeterminate	Fragment		1	
562	Mammalia	Indeterminate		Indeterminate	Fragment		1	
563	Mammalia	Indeterminate		Indeterminate	Fragment		1	
564	Mammalia	Indeterminate		Indeterminate	Fragment		1	
565	Mammalia	Indeterminate		Indeterminate	Fragment		1	
566	Mammalia	Indeterminate		Indeterminate	Fragment		1	
567	Mammalia	Indeterminate		Indeterminate	Fragment		1	
568	Mammalia	Indeterminate		Indeterminate	Fragment		1	
569	Mammalia	Indeterminate		Indeterminate	Fragment		1	
570	Mammalia	Indeterminate		Indeterminate	Fragment		1	
571	Mammalia	Indeterminate		Indeterminate	Fragment		1	
572	Mammalia	Indeterminate		Indeterminate	Fragment		1	
573	Mammalia	Indeterminate		Indeterminate	Fragment		1	
574	Mammalia	Indeterminate		Indeterminate	Fragment		1	
575	Mammalia	Indeterminate		Indeterminate	Fragment		1	
576	Mammalia	Indeterminate		Indeterminate	Fragment		1	
577	Mammalia	Indeterminate		Indeterminate	Fragment		1	
578	Mammalia	Indeterminate		Indeterminate	Fragment		1	
579	Mammalia	Indeterminate		Indeterminate	Fragment		1	
580	Mammalia	Indeterminate		Indeterminate	Fragment		1	
581	Mammalia	Indeterminate		Indeterminate	Fragment		1	
582	Mammalia	Indeterminate		Indeterminate	Fragment		1	
583	Mammalia	Indeterminate		Indeterminate	Fragment		1	
584	Mammalia	Indeterminate		Indeterminate	Fragment		1	
585	Mammalia	Indeterminate		Indeterminate	Fragment		1	
586	Mammalia	Indeterminate		Indeterminate	Fragment		1	
587	Mammalia	Indeterminate		Indeterminate	Fragment		1	
588	Mammalia	Indeterminate		Indeterminate	Fragment		1	
589	Mammalia	Indeterminate		Indeterminate	Fragment		1	
590	Mammalia	Indeterminate		Indeterminate	Fragment		1	
591	Mammalia	Indeterminate		Indeterminate	Fragment		1	
592	Mammalia	Indeterminate		Indeterminate	Fragment		1	
593	Mammalia	Indeterminate		Indeterminate	Fragment		1	
594	Mammalia	Indeterminate		Indeterminate	Fragment		1	
595	Mammalia	Indeterminate		Indeterminate	Fragment		1	
596	Mammalia	Indeterminate		Indeterminate	Fragment		1	
597	Mammalia	Indeterminate		Indeterminate	Fragment		1	
598	Mammalia	Indeterminate		Indeterminate	Fragment		1	
599	Mammalia	Indeterminate		Indeterminate	Fragment		1	

Supplemental Table S2. Catalogue of GDA Specimens: Identifiable Postcranial Specimens and Indeterminate Specimens

GA #	Class or Order	Family	Bovid Size Class	Element	Part or Portion	Side	Overall Size (cm)	Specimen Count
600	Mammalia	Indeterminate		Indeterminate	Fragment		1	
601	Mammalia	Indeterminate		Indeterminate	Fragment		1	
602	Mammalia	Indeterminate		Indeterminate	Fragment		6	
603	Mammalia	Indeterminate		Indeterminate	Fragment		6	
604	Mammalia	Indeterminate		Indeterminate	Fragment		6	
605	Mammalia	Indeterminate		Indeterminate	Fragment		5	
606	Mammalia	Indeterminate		Indeterminate	Fragment		5	
607	Mammalia	Indeterminate		Indeterminate	Fragment		4	
608	Mammalia	Indeterminate		Indeterminate	Fragment		4	
609	Mammalia	Indeterminate		Indeterminate	Fragment		4	
610	Mammalia	Indeterminate		Indeterminate	Fragment		4	
611	Mammalia	Indeterminate		Indeterminate	Fragment		4	
612	Mammalia	Indeterminate		Indeterminate	Fragment		4	
613	Mammalia	Indeterminate		Indeterminate	Fragment		4	
614	Mammalia	Indeterminate		Indeterminate	Fragment		4	
615	Mammalia	Indeterminate		Indeterminate	Fragment		4	
616	Mammalia	Indeterminate		Indeterminate	Fragment		4	
617	Mammalia	Indeterminate		Indeterminate	Fragment		4	
618	Mammalia	Indeterminate		Indeterminate	Fragment		4	
619	Mammalia	Indeterminate		Indeterminate	Fragment		4	
620	Mammalia	Indeterminate		Indeterminate	Fragment		4	
621	Mammalia	Indeterminate		Indeterminate	Fragment		4	
622	Mammalia	Indeterminate		Indeterminate	Fragment		4	
623	Mammalia	Indeterminate		Indeterminate	Fragment		4	
624	Mammalia	Indeterminate		Indeterminate	Fragment		4	
625	Mammalia	Indeterminate		Indeterminate	Fragment		4	
626	Mammalia	Indeterminate		Indeterminate	Fragment		4	
627	Mammalia	Indeterminate		Indeterminate	Fragment		4	
628	Mammalia	Indeterminate		Indeterminate	Fragment		4	
629	Mammalia	Indeterminate		Indeterminate	Fragment		4	
630	Mammalia	Indeterminate		Indeterminate	Fragment		5	
631	Mammalia	Indeterminate		Indeterminate	Fragment		5	
632	Mammalia	Indeterminate		Indeterminate	Fragment		5	
632	Mammalia	Indeterminate		Indeterminate	Fragment		5	
633	Mammalia	Indeterminate		Indeterminate	Fragment		5	
634	Mammalia	Indeterminate		Indeterminate	Fragment		5	
635	Mammalia	Indeterminate		Indeterminate	Fragment		5	
636	Mammalia	Indeterminate		Indeterminate	Fragment		5	
637	Mammalia	Indeterminate		Indeterminate	Fragment		0	N=24
638	Mammalia	Indeterminate		Indeterminate	Fragment		0	N=65
639	Mammalia	Indeterminate		Indeterminate	Fragment		0	N=1133
640	Mammalia	Indeterminate		Indeterminate	Fragment		2	
641	Mammalia	Indeterminate		Indeterminate	Fragment		2	
642	Mammalia	Indeterminate		Indeterminate	Fragment		2	
643	Mammalia	Indeterminate		Indeterminate	Fragment		1	
644	Mammalia	Indeterminate		Indeterminate	Fragment		1	
645	Cetartiodactyla	Bovidae	II	Humerus	Distal epiphysis	Right	4	
646	Mammalia	Indeterminate		Indeterminate	Fragment		3	
647	Mammalia	Indeterminate		Indeterminate	Fragment		1	
648	Mammalia	Indeterminate		Indeterminate	Fragment		1	
649	Mammalia	Indeterminate		Indeterminate	Fragment		0	N=3
650	Mammalia	Indeterminate		Indeterminate	Fragment		2	
651	Mammalia	Indeterminate		Indeterminate	Fragment		2	
652	Mammalia	Indeterminate		Indeterminate	Fragment		2	
653	Mammalia	Indeterminate		Indeterminate	Fragment		2	
654	Mammalia	Indeterminate		Indeterminate	Fragment		2	
655	Mammalia	Indeterminate		Indeterminate	Fragment		2	
656	Mammalia	Indeterminate		Indeterminate	Fragment		2	
657	Mammalia	Indeterminate		Indeterminate	Fragment		2	
658	Mammalia	Indeterminate		Indeterminate	Fragment		2	
659	Mammalia	Indeterminate		Indeterminate	Fragment		2	
660	Mammalia	Indeterminate		Indeterminate	Fragment		2	
661	Mammalia	Indeterminate		Indeterminate	Fragment		2	
662	Mammalia	Indeterminate		Indeterminate	Fragment		2	
663	Mammalia	Indeterminate		Indeterminate	Fragment		2	

Supplemental Table S2. Catalogue of GDA Specimens: Identifiable Postcranial Specimens and Indeterminate Specimens

GA #	Class or Order	Family	Bovid Size Class	Element	Part or Portion	Side	Overall Size (cm)	Specimen Count
664	Mammalia	Indeterminate		Indeterminate	Fragment		2	
665	Mammalia	Indeterminate		Indeterminate	Fragment		2	
666	Mammalia	Indeterminate		Indeterminate	Fragment		2	
667	Mammalia	Indeterminate		Indeterminate	Fragment		2	
668	Mammalia	Indeterminate		Indeterminate	Fragment		2	
669	Mammalia	Indeterminate		Indeterminate	Fragment		2	
670	Mammalia	Indeterminate		Indeterminate	Fragment		2	
671	Mammalia	Indeterminate		Indeterminate	Fragment		2	
672	Mammalia	Indeterminate		Indeterminate	Fragment		2	
673	Mammalia	Indeterminate		Indeterminate	Fragment		2	
674	Mammalia	Indeterminate		Indeterminate	Fragment		2	
675	Mammalia	Indeterminate		Indeterminate	Fragment		2	
676	Mammalia	Indeterminate		Indeterminate	Fragment		2	
677	Mammalia	Indeterminate		Indeterminate	Fragment		2	
678	Mammalia	Indeterminate		Indeterminate	Fragment		2	
679	Mammalia	Indeterminate		Indeterminate	Fragment		2	
680	Mammalia	Indeterminate		Indeterminate	Fragment		2	
681	Mammalia	Indeterminate		Indeterminate	Fragment		2	
682	Mammalia	Indeterminate		Indeterminate	Fragment		2	
683	Mammalia	Indeterminate		Indeterminate	Fragment		2	
684	Mammalia	Indeterminate		Indeterminate	Fragment		2	
685	Mammalia	Indeterminate		Indeterminate	Fragment		2	
686	Mammalia	Indeterminate		Indeterminate	Fragment		2	
687	Mammalia	Indeterminate		Indeterminate	Fragment		2	
688	Mammalia	Indeterminate		Indeterminate	Fragment		2	
689	Mammalia	Indeterminate		Indeterminate	Fragment		2	
690	Mammalia	Indeterminate		Indeterminate	Fragment		2	
691	Mammalia	Indeterminate		Indeterminate	Fragment		2	
692	Mammalia	Indeterminate		Indeterminate	Fragment		2	
693	Mammalia	Indeterminate		Indeterminate	Fragment		2	
694	Mammalia	Indeterminate		Indeterminate	Fragment		2	
695	Mammalia	Indeterminate		Indeterminate	Fragment		2	
696	Mammalia	Indeterminate		Indeterminate	Fragment		2	
697	Mammalia	Indeterminate		Indeterminate	Fragment		2	
698	Mammalia	Indeterminate		Indeterminate	Fragment		2	
699	Mammalia	Indeterminate		Indeterminate	Fragment		2	
700	Mammalia	Indeterminate		Indeterminate	Fragment		4	
701	Mammalia	Indeterminate		Indeterminate	Fragment		4	
702	Mammalia	Indeterminate		Indeterminate	Fragment		1	
703	Mammalia	Indeterminate		Indeterminate	Fragment		1	
704	Mammalia	Indeterminate		Indeterminate	Fragment		2	
705	Mammalia	Indeterminate		Indeterminate	Fragment		2	
706	Mammalia	Indeterminate		Indeterminate	Fragment		3	
707	Mammalia	Indeterminate		Indeterminate	Fragment		6	
708	Mammalia	Indeterminate		Indeterminate	Fragment		6	
709	Mammalia	Indeterminate		Cranial	Enamel Fragment		1	
710	Mammalia	Indeterminate		Indeterminate	Fragment		3	
711	Mammalia	Indeterminate		Indeterminate	Fragment		3	
712	Mammalia	Indeterminate		Indeterminate	Fragment		3	
713	Mammalia	Indeterminate		Indeterminate	Fragment		3	
714	Mammalia	Indeterminate		Indeterminate	Fragment		3	
715	Mammalia	Indeterminate		Indeterminate	Fragment		3	
716	Cetartiodactyla	Bovidae	II	Intermediate Phalanx	Complete		2	
717	Mammalia	Indeterminate		Indeterminate	Fragment		6	
718	Mammalia	Indeterminate		Indeterminate	Fragment		4	
719	Mammalia	Indeterminate		Indeterminate	Fragment		4	
720	Mammalia	Indeterminate		Rib	Proximal epiphysis		2	
721	Mammalia	Indeterminate		Cranial	Enamel Fragment		1	
722	Mammalia	Indeterminate		Indeterminate	Fragment		1	
723	Mammalia	Indeterminate		Indeterminate	Fragment		1	
724	Mammalia	Indeterminate		Cranial	Enamel Fragment		1	
725	Mammalia	Indeterminate		Indeterminate	Fragment		1	
726	Mammalia	Indeterminate		Cranial	Enamel Fragment		1	
727	Mammalia	Indeterminate		Indeterminate	Fragment		1	
728	Mammalia	Indeterminate		Indeterminate	Fragment		2	

Supplemental Table S2. Catalogue of GDA Specimens: Identifiable Postcranial Specimens and Indeterminate Specimens

GA #	Class or Order	Family	Bovid Size Class	Element	Part or Portion	Side	Overall Size (cm)	Specimen Count
729	Mammalia	Indeterminate		Indeterminate	Fragment		1	
730	Mammalia	Indeterminate		Indeterminate	Fragment		1	
731	Mammalia	Indeterminate		Indeterminate	Fragment		1	
732	Mammalia	Indeterminate		Indeterminate	Fragment		1	
733	Mammalia	Indeterminate		Indeterminate	Fragment		1	
734	Mammalia	Indeterminate		Indeterminate	Fragment		1	
735	Mammalia	Indeterminate		Indeterminate	Fragment		1	
736	Mammalia	Indeterminate		Indeterminate	Fragment		1	
737	Mammalia	Indeterminate		Indeterminate	Fragment		1	
738	Mammalia	Indeterminate		Indeterminate	Fragment		1	
739	Mammalia	Indeterminate		Indeterminate	Fragment		1	
740	Mammalia	Indeterminate		Indeterminate	Fragment		1	
741	Mammalia	Indeterminate		Indeterminate	Fragment		1	
742	Mammalia	Indeterminate		Cranial	Fragment		2	
743	Mammalia	Indeterminate		Indeterminate	Fragment		1	
744	Mammalia	Indeterminate		Vertebra	Fragment		1	
745	Mammalia	Indeterminate		Indeterminate	Fragment		1	
746	Mammalia	Indeterminate		Indeterminate	Fragment		1	
747	Mammalia	Indeterminate		Indeterminate	Fragment		1	
748	Mammalia	Indeterminate		Indeterminate	Fragment		2	
749	Mammalia	Indeterminate		Indeterminate	Fragment		1	
750	Mammalia	Indeterminate		Indeterminate	Fragment		3	
751	Mammalia	Indeterminate		Indeterminate	Fragment		4	
752	Mammalia	Indeterminate		Indeterminate	Fragment		1	
753	Mammalia	Indeterminate		Indeterminate	Fragment		1	
754	Mammalia	Indeterminate		Indeterminate	Fragment		1	
755	Mammalia	Indeterminate		Rib	Diaphysis		2	
756	Cetartiodactyla	Bovidae	II	Vertebra	Enamel Fragment		4	
757	Mammalia	Indeterminate		Vertebra	Fragment		4	
758	Mammalia	Indeterminate		Indeterminate	Fragment		2	
759	Mammalia	Indeterminate		Cranial	Fragment		1	
760	Mammalia	Indeterminate		Cranial	Fragment		1	
761	Mammalia	Indeterminate		Cranial	Temporal		2	
762	Cetartiodactyla	Bovidae	II	Metacarpal	Proximal epiphysis	Left	7	
763	Mammalia	Indeterminate		Indeterminate	Fragment		3	
764	Mammalia	Indeterminate		Indeterminate	Fragment		2	
765	Mammalia	Indeterminate		Indeterminate	Fragment		2	
766	Mammalia	Indeterminate		Indeterminate	Fragment		2	
767	Mammalia	Indeterminate		Indeterminate	Fragment		2	
768	Mammalia	Indeterminate		Cranial	Fragment		2	
769	Mammalia	Indeterminate		Indeterminate	Fragment		2	
770	Mammalia	Indeterminate		Indeterminate	Fragment		2	
771	Mammalia	Indeterminate		Indeterminate	Fragment		2	
772	Mammalia	Indeterminate		Indeterminate	Fragment		2	
773	Mammalia	Indeterminate		Indeterminate	Fragment		2	
774	Mammalia	Indeterminate		Indeterminate	Fragment		2	
775	Mammalia	Indeterminate		Indeterminate	Fragment		2	
776	Mammalia	Indeterminate		Indeterminate	Fragment		2	
777	Mammalia	Indeterminate		Indeterminate	Fragment		2	
778	Mammalia	Indeterminate		Indeterminate	Fragment		2	
779	Mammalia	Indeterminate		Indeterminate	Fragment		2	
780	Mammalia	Indeterminate		Indeterminate	Fragment		2	
781	Mammalia	Indeterminate		Indeterminate	Fragment		2	
782	Cetartiodactyla	Bovidae	I	Proximal Phalanx	Distal epiphysis		2	
783	Mammalia	Indeterminate		Indeterminate	Fragment		2	
784	Mammalia	Indeterminate		Indeterminate	Fragment		2	
785	Mammalia	Indeterminate		Indeterminate	Fragment		2	
786	Mammalia	Indeterminate		Indeterminate	Fragment		2	
787	Mammalia	Indeterminate		Indeterminate	Fragment		2	
788	Mammalia	Indeterminate		Indeterminate	Fragment		1	
789	Mammalia	Indeterminate		Indeterminate	Fragment		2	
790	Mammalia	Indeterminate		Indeterminate	Fragment		2	
791	Mammalia	Indeterminate		Indeterminate	Fragment		2	
792	Mammalia	Indeterminate		Indeterminate	Fragment		2	
793	Cetartiodactyla	Bovidae	II	Metapodial	Diaphysis		2	

Supplemental Table S2. Catalogue of GDA Specimens: Identifiable Postcranial Specimens and Indeterminate Specimens

GA #	Class or Order	Family	Bovid Size Class	Element	Part or Portion	Side	Overall Size (cm)	Specimen Count
794	Mammalia	Indeterminate		Indeterminate	Fragment		2	
795	Mammalia	Indeterminate		Indeterminate	Fragment		3	
796	Mammalia	Indeterminate		Indeterminate	Fragment		3	
797	Mammalia	Indeterminate		Indeterminate	Fragment		3	
798	Mammalia	Indeterminate		Indeterminate	Fragment		3	
799	Mammalia	Indeterminate		Indeterminate	Fragment		3	
800	Mammalia	Indeterminate		Indeterminate	Fragment		3	
801	Mammalia	Indeterminate		Indeterminate	Fragment		3	
802	Mammalia	Indeterminate		Indeterminate	Fragment		3	
803	Mammalia	Indeterminate		Indeterminate	Fragment		3	
804	Mammalia	Indeterminate		Indeterminate	Fragment		3	
805	Mammalia	Indeterminate		Indeterminate	Fragment		3	
806	Mammalia	Indeterminate		Indeterminate	Fragment		3	
807	Mammalia	Indeterminate		Indeterminate	Fragment		3	
808	Mammalia	Indeterminate		Indeterminate	Fragment		3	
809	Mammalia	Indeterminate		Indeterminate	Fragment		3	
810	Mammalia	Indeterminate		Indeterminate	Fragment		3	
811	Mammalia	Indeterminate		Indeterminate	Fragment		3	
812	Mammalia	Indeterminate		Indeterminate	Fragment		3	
813	Mammalia	Indeterminate		Indeterminate	Fragment		1	
814	Mammalia	Indeterminate		Indeterminate	Fragment		1	
815	Mammalia	Indeterminate		Indeterminate	Fragment		1	
816	Mammalia	Indeterminate		Indeterminate	Fragment		1	
817	Mammalia	Indeterminate		Indeterminate	Fragment		1	
818	Mammalia	Indeterminate		Indeterminate	Fragment		1	
819	Mammalia	Indeterminate		Indeterminate	Fragment		1	
820	Mammalia	Indeterminate		Indeterminate	Fragment		1	
821	Mammalia	Indeterminate		Indeterminate	Fragment		1	
822	Mammalia	Indeterminate		Indeterminate	Fragment		1	
823	Mammalia	Indeterminate		Indeterminate	Fragment		1	
824	Mammalia	Indeterminate		Indeterminate	Fragment		1	
825	Mammalia	Indeterminate		Indeterminate	Fragment		1	
826	Mammalia	Indeterminate		Indeterminate	Fragment		1	
827	Mammalia	Indeterminate		Indeterminate	Fragment		1	
828	Mammalia	Indeterminate		Indeterminate	Fragment		1	
829	Mammalia	Indeterminate		Indeterminate	Fragment		1	
830	Mammalia	Indeterminate		Indeterminate	Fragment		1	
831	Mammalia	Indeterminate		Indeterminate	Fragment		1	
832	Mammalia	Indeterminate		Indeterminate	Fragment		1	
833	Mammalia	Indeterminate		Indeterminate	Fragment		2	
834	Mammalia	Indeterminate		Indeterminate	Fragment		2	
835	Mammalia	Indeterminate		Indeterminate	Fragment		2	
836	Mammalia	Indeterminate		Indeterminate	Fragment		2	
837	Cetartiodactyla	Bovidae	II	Sesamoid	Distal		0	
838	Cetartiodactyla	Bovidae	II	Sesamoid	Distal		0	
840	Mammalia	Indeterminate		Cranial	Enamel Fragment		2	
845	Mammalia	Indeterminate		Cranial	Enamel Fragment		2	
847	Cetartiodactyla	Bovidae		Cranial	Enamel Fragment		4	
848	Mammalia	Indeterminate		Indeterminate	Fragment		2	
849	Mammalia	Indeterminate		Cranial	Enamel Fragment		1	
850	Mammalia	Indeterminate		Cranial	Enamel Fragment		1	
851	Mammalia	Indeterminate		Indeterminate	Fragment		1	
852	Mammalia	Indeterminate		Cranial	Enamel Fragment		1	
853	Mammalia	Indeterminate		Cranial	Enamel Fragment		1	
854	Mammalia	Indeterminate		Cranial	Enamel Fragment		1	
855	Mammalia	Indeterminate		Cranial	Enamel Fragment		1	
857	Cetartiodactyla	Bovidae		Cranial	Enamel Fragment		1	
860	Mammalia	Indeterminate		Cranial	Enamel Fragment		1	
861	Mammalia	Indeterminate		Cranial	Enamel Fragment		1	
862	Cetartiodactyla	Bovidae		Cranial	Enamel Fragment		1	
863	Mammalia	Indeterminate		Cranial	Enamel Fragment		1	
864	Mammalia	Indeterminate		Cranial	Enamel Fragment		1	
865	Mammalia	Indeterminate		Cranial	Enamel Fragment		1	
866	Mammalia	Indeterminate		Cranial	Enamel Fragment		0	
867	Mammalia	Indeterminate		Cranial	Enamel Fragment		0	

Supplemental Table S2. Catalogue of GDA Specimens: Identifiable Postcranial Specimens and Indeterminate Specimens

GA #	Class or Order	Family	Bovid Size Class	Element	Part or Portion	Side	Overall Size (cm)	Specimen Count
869	Mammalia	Indeterminate		Cranial	Enamel Fragment		1	
870	Mammalia	Indeterminate		Cranial	Enamel Fragment		1	
871	Mammalia	Indeterminate		Cranial	Enamel Fragment		1	
872	Mammalia	Indeterminate		Cranial	Enamel Fragment		1	
873	Cetartiodactyla	Bovidae	II	Metapodial	Distal epiphysis		1	
874	Mammalia	Indeterminate		Cranial	Enamel Fragment		1	
875	Mammalia	Indeterminate		Cranial	Enamel Fragment		1	
876	Mammalia	Indeterminate		Indeterminate	Fragment		2	
877	Mammalia	Indeterminate		Cranial	Enamel Fragment		1	
878	Mammalia	Indeterminate		Cranial	Enamel Fragment		2	
879	Mammalia	Indeterminate		Cranial	Enamel Fragment		2	
881	Mammalia	Indeterminate		Cranial	Enamel Fragment		3	
882	Mammalia	Indeterminate		Cranial	Enamel Fragment		3	
883	Mammalia	Indeterminate		Indeterminate	Fragment		1	
884	Mammalia	Indeterminate		Cranial	Enamel Fragment		0	
885	Mammalia	Indeterminate		Cranial	Enamel Fragment		1	
886	Cetartiodactyla	Bovidae		Cranial	Enamel Fragment		1	
887	Mammalia	Indeterminate		Cranial	Enamel Fragment		1	
888	Cetartiodactyla	Bovidae		Cranial	Enamel Fragment		2	
889	Mammalia	Indeterminate		Indeterminate	Fragment		2	
890	Cetartiodactyla	Bovidae		Cranial	Enamel Fragment		2	
891	Mammalia	Indeterminate		Cranial	Enamel Fragment		3	
892	Cetartiodactyla	Bovidae	II	Vertebra	Lumbar		3	
893	Mammalia	Indeterminate		Indeterminate	Fragment		1	
894	Mammalia	Indeterminate		Indeterminate	Fragment		1	
895	Cetartiodactyla	Bovidae		Cranial	Enamel Fragment		1	
896	Mammalia	Indeterminate		Indeterminate	Fragment		2	
897	Cetartiodactyla	Bovidae	II	Carpal	Second	Left	2	
898	Cetartiodactyla	Bovidae		Cranial	Enamel Fragment		1	
899	Mammalia	Indeterminate		Cranial	Enamel Fragment		1	
900	Cetartiodactyla	Bovidae	II	Sesamoid	Distal epiphysis		0	
901	Cetartiodactyla	Bovidae	II	Sesamoid	Proximal epiphysis		1	
902	Mammalia	Indeterminate		Cranial	Enamel Fragment		1	
903	Cetartiodactyla	Bovidae		Cranial	Enamel Fragment		2	
904	Mammalia	Indeterminate		Indeterminate	Fragment		1	
905	Cetartiodactyla	Bovidae		Metapodial	Distal epiphysis		1	
906	Mammalia	Indeterminate		Indeterminate	Fragment		1	
907	Mammalia	Indeterminate		Indeterminate	Fragment		1	
908	Mammalia	Indeterminate		Indeterminate	Fragment		1	
909	Mammalia	Indeterminate		Indeterminate	Fragment		1	
910	Mammalia	Indeterminate		Indeterminate	Fragment		1	
911	Mammalia	Indeterminate		Indeterminate	Fragment		1	
912	Mammalia	Indeterminate		Indeterminate	Fragment		1	
913	Mammalia	Indeterminate		Indeterminate	Fragment		1	
914	Mammalia	Indeterminate		Indeterminate	Fragment		1	
915	Mammalia	Indeterminate		Indeterminate	Fragment		1	
916	Mammalia	Indeterminate		Indeterminate	Fragment		1	
917	Mammalia	Indeterminate		Indeterminate	Fragment		1	
918	Mammalia	Indeterminate		Indeterminate	Fragment		1	
919	Mammalia	Indeterminate		Indeterminate	Fragment		1	
920	Mammalia	Indeterminate		Indeterminate	Fragment		1	
921	Mammalia	Indeterminate		Indeterminate	Fragment		1	
922	Mammalia	Indeterminate		Indeterminate	Fragment		1	
923	Mammalia	Indeterminate		Indeterminate	Fragment		1	
924	Mammalia	Indeterminate		Indeterminate	Fragment		1	
925	Mammalia	Indeterminate		Indeterminate	Fragment		1	
926	Mammalia	Indeterminate		Indeterminate	Fragment		1	
927	Mammalia	Indeterminate		Indeterminate	Fragment		1	
928	Mammalia	Indeterminate		Indeterminate	Fragment		1	
929	Mammalia	Indeterminate		Indeterminate	Fragment		1	
930	Mammalia	Indeterminate		Indeterminate	Fragment		1	
931	Mammalia	Indeterminate		Indeterminate	Fragment		1	
932	Mammalia	Indeterminate		Indeterminate	Fragment		1	
933	Mammalia	Indeterminate		Indeterminate	Fragment		1	
934	Mammalia	Indeterminate		Indeterminate	Fragment		1	

Supplemental Table S2. Catalogue of GDA Specimens: Identifiable Postcranial Specimens and Indeterminate Specimens

GA #	Class or Order	Family	Bovid Size Class	Element	Part or Portion	Side	Overall Size (cm)	Specimen Count
935	Mammalia	Indeterminate		Indeterminate	Fragment		1	
936	Mammalia	Indeterminate		Indeterminate	Fragment		1	
937	Mammalia	Indeterminate		Indeterminate	Fragment		1	
938	Mammalia	Indeterminate		Indeterminate	Fragment		1	
939	Mammalia	Indeterminate		Indeterminate	Fragment		1	
940	Mammalia	Indeterminate		Indeterminate	Fragment		1	
941	Mammalia	Indeterminate		Indeterminate	Fragment		1	
942	Mammalia	Indeterminate		Indeterminate	Fragment		1	
943	Mammalia	Indeterminate		Indeterminate	Fragment		1	
945	Mammalia	Indeterminate		Indeterminate	Fragment		1	
946	Mammalia	Indeterminate		Indeterminate	Fragment		1	
947	Mammalia	Indeterminate		Indeterminate	Fragment		1	
948	Mammalia	Indeterminate		Indeterminate	Fragment		1	
949	Mammalia	Indeterminate		Indeterminate	Fragment		1	
950	Mammalia	Indeterminate		Indeterminate	Fragment		1	
951	Mammalia	Indeterminate		Indeterminate	Fragment		1	
952	Mammalia	Indeterminate		Indeterminate	Fragment		4	
953	Mammalia	Indeterminate		Indeterminate	Fragment		4	
954	Mammalia	Indeterminate		Indeterminate	Fragment		4	
955	Mammalia	Indeterminate		Indeterminate	Fragment		5	
956	Mammalia	Indeterminate		Indeterminate	Fragment		1	
957	Mammalia	Indeterminate		Indeterminate	Fragment		1	
958	Mammalia	Indeterminate		Indeterminate	Fragment		1	
959	Mammalia	Indeterminate		Indeterminate	Fragment		1	
960	Mammalia	Indeterminate		Indeterminate	Fragment		1	
961	Mammalia	Indeterminate		Indeterminate	Fragment		1	
962	Mammalia	Indeterminate		Indeterminate	Fragment		1	
963	Mammalia	Indeterminate		Indeterminate	Fragment		1	
964	Mammalia	Indeterminate		Indeterminate	Fragment		1	
965	Mammalia	Indeterminate		Indeterminate	Fragment		1	
966	Mammalia	Indeterminate		Indeterminate	Fragment		1	
967	Mammalia	Indeterminate		Indeterminate	Fragment		1	
968	Mammalia	Indeterminate		Indeterminate	Fragment		1	
969	Mammalia	Indeterminate		Indeterminate	Fragment		1	
970	Mammalia	Indeterminate		Indeterminate	Fragment		1	
971	Mammalia	Indeterminate		Indeterminate	Fragment		1	
972	Mammalia	Indeterminate		Indeterminate	Fragment		1	
973	Mammalia	Indeterminate		Cranial	Enamel Fragment		1	
974	Mammalia	Indeterminate		Indeterminate	Fragment		1	
975	Mammalia	Indeterminate		Indeterminate	Fragment		1	
976	Mammalia	Indeterminate		Indeterminate	Fragment		1	
977	Mammalia	Indeterminate		Indeterminate	Fragment		1	
979	Mammalia	Indeterminate		Indeterminate	Fragment		1	
980	Mammalia	Indeterminate		Indeterminate	Fragment		1	
981	Mammalia	Indeterminate		Indeterminate	Fragment		1	
982	Mammalia	Indeterminate		Indeterminate	Fragment		1	
983	Mammalia	Indeterminate		Indeterminate	Fragment		1	
984	Mammalia	Indeterminate		Indeterminate	Fragment		1	
985	Mammalia	Indeterminate		Indeterminate	Fragment		1	
986	Mammalia	Indeterminate		Indeterminate	Fragment		1	
987	Mammalia	Indeterminate		Indeterminate	Fragment		1	
988	Mammalia	Indeterminate		Indeterminate	Fragment		1	
989	Mammalia	Indeterminate		Indeterminate	Fragment		1	
990	Mammalia	Indeterminate		Indeterminate	Fragment		1	
991	Mammalia	Indeterminate		Indeterminate	Fragment		1	
992	Mammalia	Indeterminate		Indeterminate	Fragment		1	
994	Mammalia	Indeterminate		Indeterminate	Fragment		1	
995	Mammalia	Indeterminate		Indeterminate	Fragment		1	
997	Mammalia	Indeterminate		Indeterminate	Fragment		1	
998	Mammalia	Indeterminate		Indeterminate	Fragment		1	
1000	Mammalia	Indeterminate		Indeterminate	Fragment		1	
1001	Mammalia	Indeterminate		Indeterminate	Fragment		1	
1002	Mammalia	Indeterminate		Indeterminate	Fragment		1	
1003	Mammalia	Indeterminate		Indeterminate	Fragment		1	
1004	Mammalia	Indeterminate		Indeterminate	Fragment		1	

Supplemental Table S2. Catalogue of GDA Specimens: Identifiable Postcranial Specimens and Indeterminate Specimens

GA #	Class or Order	Family	Bovid Size Class	Element	Part or Portion	Side	Overall Size (cm)	Specimen Count
1005	Mammalia	Indeterminate		Indeterminate	Fragment		1	
1006	Mammalia	Indeterminate		Indeterminate	Fragment		1	
1020	Mammalia	Indeterminate		Indeterminate	Fragment		4	
1021	Mammalia	Indeterminate		Indeterminate	Fragment		4	
1022	Mammalia	Indeterminate		Indeterminate	Fragment		4	
1023	Mammalia	Indeterminate		Indeterminate	Fragment		4	
1024	Mammalia	Indeterminate		Indeterminate	Fragment		4	
1025	Mammalia	Indeterminate		Indeterminate	Fragment		4	
1026	Cetartiodactyla	Bovidae		Cranial	Enamel Fragment		1	
1027	Mammalia	Indeterminate		Cranial	Enamel Fragment		1	
1030	Mammalia	Indeterminate		Indeterminate	Fragment		2	
1031	Mammalia	Indeterminate		Indeterminate	Fragment		2	
1032	Mammalia	Indeterminate		Indeterminate	Fragment		2	
1033	Mammalia	Indeterminate		Indeterminate	Fragment		2	
1034	Mammalia	Indeterminate		Indeterminate	Fragment		2	
1035	Mammalia	Indeterminate		Indeterminate	Fragment		2	
1036	Mammalia	Indeterminate		Indeterminate	Fragment		2	
1037	Mammalia	Indeterminate		Indeterminate	Fragment		2	
1038	Mammalia	Indeterminate		Indeterminate	Fragment		2	
1039	Mammalia	Indeterminate		Indeterminate	Fragment		2	
1040	Mammalia	Indeterminate		Indeterminate	Fragment		2	
1041	Mammalia	Indeterminate		Indeterminate	Fragment		2	
1042	Mammalia	Indeterminate		Indeterminate	Fragment		1	
1043	Mammalia	Indeterminate		Indeterminate	Fragment		2	
1044	Mammalia	Indeterminate		Indeterminate	Fragment		2	
1045	Mammalia	Indeterminate		Indeterminate	Fragment		2	
1046	Mammalia	Indeterminate		Indeterminate	Fragment		2	
1047	Mammalia	Indeterminate		Indeterminate	Fragment		2	
1048	Mammalia	Indeterminate		Indeterminate	Fragment		2	
1049	Mammalia	Indeterminate		Indeterminate	Fragment		2	
1050	Mammalia	Indeterminate		Indeterminate	Fragment		2	
1051	Mammalia	Indeterminate		Indeterminate	Fragment		2	
1052	Mammalia	Indeterminate		Indeterminate	Fragment		2	
1053	Cetartiodactyla	Bovidae		Cranial	Enamel Fragment		0	
1054	Mammalia	Indeterminate		Cranial	Enamel Fragment		1	
1055	Mammalia	Indeterminate		Cranial	Enamel Fragment		2	
1056	Cetartiodactyla	Bovidae		Cranial	Enamel Fragment		2	
1057	Mammalia	Indeterminate		Indeterminate	Fragment		2	
1058	Mammalia	Indeterminate		Indeterminate	Fragment		2	
1059	Mammalia	Indeterminate		Indeterminate	Fragment		2	
1060	Mammalia	Indeterminate		Indeterminate	Fragment		2	
1061	Mammalia	Indeterminate		Indeterminate	Fragment		3	
1062	Mammalia	Indeterminate		Indeterminate	Fragment		3	
1063	Cetartiodactyla	Bovidae		Cranial	Maxillary molar		2	
1064	Mammalia	Indeterminate		Indeterminate	Fragment		3	
1065	Mammalia	Indeterminate		Indeterminate	Fragment		3	
1066	Mammalia	Indeterminate		Indeterminate	Fragment		3	
1067	Mammalia	Indeterminate		Indeterminate	Fragment		3	
1068	Mammalia	Indeterminate		Indeterminate	Fragment		3	
1069	Mammalia	Indeterminate		Indeterminate	Fragment		3	
1070	Mammalia	Indeterminate		Indeterminate	Fragment		3	
1071	Mammalia	Indeterminate		Indeterminate	Fragment		3	
1072	Mammalia	Indeterminate		Indeterminate	Fragment		3	
1073	Cetartiodactyla	Bovidae	III	Metacarpal	Proximal epiphysis	Right	4	
1074	Mammalia	Indeterminate		Indeterminate	Fragment		5	
1075	Mammalia	Indeterminate		Indeterminate	Fragment		5	
1076	Mammalia	Indeterminate		Indeterminate	Fragment		5	
1077	Mammalia	Indeterminate		Indeterminate	Fragment		5	
1078	Mammalia	Indeterminate		Indeterminate	Fragment		0	N=22
1079	Mammalia	Indeterminate		Indeterminate	Fragment		1	
1080	Mammalia	Indeterminate		Indeterminate	Fragment		1	
1081	Mammalia	Indeterminate		Indeterminate	Fragment		1	
1082	Mammalia	Indeterminate		Indeterminate	Fragment		1	
1083	Mammalia	Indeterminate		Indeterminate	Fragment		1	
1084	Mammalia	Indeterminate		Indeterminate	Fragment		1	

Supplemental Table S2. Catalogue of GDA Specimens: Identifiable Postcranial Specimens and Indeterminate Specimens

GA #	Class or Order	Family	Bovid Size Class	Element	Part or Portion	Side	Overall Size (cm)	Specimen Count
1085	Mammalia	Indeterminate		Indeterminate	Fragment		2	
1086	Mammalia	Indeterminate		Indeterminate	Fragment		4	
1087	Cetartiodactyla	Bovidae		Cranial	Enamel Fragment		1	
1088	Mammalia	Indeterminate		Cranial	Enamel Fragment		1	
1089	Mammalia	Indeterminate		Cranial	Enamel Fragment		1	
1090	Cetartiodactyla	Bovidae		Cranial	Enamel Fragment		1	
1092	Mammalia	Indeterminate		Indeterminate	Fragment		1	
1093	Mammalia	Indeterminate		Cranial	Enamel Fragment		1	
1094	Cetartiodactyla	Bovidae		Cranial	Molar		1	
1095	Cetartiodactyla	Bovidae		Cranial	Enamel Fragment		1	
1096	Cetartiodactyla	Bovidae		Cranial	Enamel Fragment		1	
1097	Mammalia	Indeterminate		Cranial	Enamel Fragment		1	
1098	Cetartiodactyla	Bovidae		Cranial	Enamel Fragment		1	
1100	Cetartiodactyla	Bovidae		Cranial	Enamel Fragment		1	
1101	Cetartiodactyla	Bovidae		Cranial	Enamel Fragment		1	
1102	Cetartiodactyla	Bovidae		Cranial	Enamel Fragment		1	
1103	Mammalia	Indeterminate		Cranial	Enamel Fragment		1	
1104	Mammalia	Indeterminate		Cranial	Enamel Fragment		1	
1105	Cetartiodactyla	Bovidae		Cranial	Enamel Fragment		1	
1106	Mammalia	Indeterminate		Cranial	Enamel Fragment		1	
1107	Mammalia	Indeterminate		Cranial	Enamel Fragment		1	
1108	Mammalia	Indeterminate		Indeterminate	Fragment		1	
1109	Cetartiodactyla	Bovidae		Cranial	Enamel Fragment		1	
1109	Mammalia	Indeterminate		Indeterminate	Fragment		1	
1110	Cetartiodactyla	Bovidae		Cranial	Enamel Fragment		1	
1110	Mammalia	Indeterminate		Indeterminate	Fragment		1	
1111	Cetartiodactyla	Bovidae		Cranial	Enamel Fragment		1	
1111	Mammalia	Indeterminate		Indeterminate	Fragment		1	
1112	Cetartiodactyla	Bovidae		Cranial	Enamel Fragment		1	
1112	Mammalia	Indeterminate		Indeterminate	Fragment		1	
1113	Cetartiodactyla	Bovidae		Cranial	Enamel Fragment		2	
1113	Mammalia	Indeterminate		Cranial	Enamel Fragment		1	
1114	Cetartiodactyla	Bovidae		Cranial	Enamel Fragment		1	
1114	Mammalia	Indeterminate		Indeterminate	Fragment		1	
1115	Mammalia	Indeterminate		Indeterminate	Fragment		1	
1116	Mammalia	Indeterminate		Indeterminate	Fragment		1	
1117	Cetartiodactyla	Bovidae	II	Proximal Phalanx	Proximal epiphysis		1	
1117	Mammalia	Indeterminate		Indeterminate	Fragment		1	
1118	Mammalia	Indeterminate		Indeterminate	Fragment		1	
1119	Mammalia	Indeterminate		Indeterminate	Fragment		1	
1120	Mammalia	Indeterminate		Indeterminate	Fragment		1	
1121	Mammalia	Indeterminate		Indeterminate	Fragment		1	
1122	Mammalia	Indeterminate		Cranial	Fragment		1	
1123	Mammalia	Indeterminate		Indeterminate	Fragment		1	
1124	Mammalia	Indeterminate		Indeterminate	Fragment		1	
1125	Mammalia	Indeterminate		Indeterminate	Fragment		1	
1126	Mammalia	Indeterminate		Indeterminate	Fragment		1	
1127	Mammalia	Indeterminate		Indeterminate	Fragment		1	
1128	Mammalia	Indeterminate		Indeterminate	Fragment		1	
1129	Mammalia	Indeterminate		Indeterminate	Fragment		1	
1130	Mammalia	Indeterminate		Indeterminate	Fragment		1	
1131	Mammalia	Indeterminate		Indeterminate	Fragment		1	
1132	Mammalia	Indeterminate		Indeterminate	Fragment		1	
1133	Mammalia	Indeterminate		Indeterminate	Fragment		1	
1134	Mammalia	Indeterminate		Indeterminate	Fragment		1	
1135	Mammalia	Indeterminate		Indeterminate	Fragment		1	
1136	Mammalia	Indeterminate		Indeterminate	Fragment		1	
1137	Mammalia	Indeterminate		Indeterminate	Fragment		1	
1138	Mammalia	Indeterminate		Indeterminate	Fragment		1	
1139	Mammalia	Indeterminate		Indeterminate	Fragment		1	
1140	Mammalia	Indeterminate		Indeterminate	Fragment		1	
1141	Mammalia	Indeterminate		Indeterminate	Fragment		1	
1142	Mammalia	Indeterminate		Indeterminate	Fragment		1	
1143	Mammalia	Indeterminate		Indeterminate	Fragment		1	
1144	Mammalia	Indeterminate		Indeterminate	Fragment		1	

Supplemental Table S2. Catalogue of GDA Specimens: Identifiable Postcranial Specimens and Indeterminate Specimens

GA #	Class or Order	Family	Bovid Size Class	Element	Part or Portion	Side	Overall Size (cm)	Specimen Count
1145	Mammalia	Indeterminate		Indeterminate	Fragment		1	
1146	Mammalia	Indeterminate		Indeterminate	Fragment		1	
1147	Mammalia	Indeterminate		Indeterminate	Fragment		1	
1148	Mammalia	Indeterminate		Indeterminate	Fragment		1	
1149	Mammalia	Indeterminate		Indeterminate	Fragment		1	
1150	Mammalia	Indeterminate		Indeterminate	Fragment		1	
1151	Mammalia	Indeterminate		Indeterminate	Fragment		1	
1152	Mammalia	Indeterminate		Indeterminate	Fragment		1	
1153	Mammalia	Indeterminate		Indeterminate	Fragment		1	
1154	Mammalia	Indeterminate		Indeterminate	Fragment		1	
1155	Mammalia	Indeterminate		Indeterminate	Fragment		1	
1156	Mammalia	Indeterminate		Indeterminate	Fragment		1	
1157	Mammalia	Indeterminate		Indeterminate	Fragment		1	
1158	Mammalia	Indeterminate		Indeterminate	Fragment		1	
1159	Mammalia	Indeterminate		Indeterminate	Fragment		1	
1160	Mammalia	Indeterminate		Indeterminate	Fragment		1	
1161	Mammalia	Indeterminate		Indeterminate	Fragment		1	
1162	Mammalia	Indeterminate		Indeterminate	Fragment		1	
1163	Mammalia	Indeterminate		Indeterminate	Fragment		1	
1164	Mammalia	Indeterminate		Indeterminate	Fragment		1	
1165	Mammalia	Indeterminate		Indeterminate	Fragment		1	
1166	Mammalia	Indeterminate		Indeterminate	Fragment		1	
1167	Mammalia	Indeterminate		Cranial	Fragment		1	
1168	Mammalia	Indeterminate		Indeterminate	Fragment		1	
1169	Mammalia	Indeterminate		Indeterminate	Fragment		1	
1170	Mammalia	Indeterminate		Indeterminate	Fragment		1	
1171	Mammalia	Indeterminate		Indeterminate	Fragment		1	
1172	Mammalia	Indeterminate		Indeterminate	Fragment		1	
1173	Mammalia	Indeterminate		Indeterminate	Fragment		1	
1174	Mammalia	Indeterminate		Indeterminate	Fragment		1	
1175	Mammalia	Indeterminate		Indeterminate	Fragment		1	
1176	Mammalia	Indeterminate		Indeterminate	Fragment		1	
1177	Mammalia	Indeterminate		Indeterminate	Fragment		1	
1178	Mammalia	Indeterminate		Indeterminate	Fragment		1	
1179	Mammalia	Indeterminate		Indeterminate	Fragment		1	
1180	Mammalia	Indeterminate		Indeterminate	Fragment		1	
1181	Mammalia	Indeterminate		Indeterminate	Fragment		1	
1182	Mammalia	Indeterminate		Indeterminate	Fragment		1	
1183	Mammalia	Indeterminate		Indeterminate	Fragment		1	
1184	Mammalia	Indeterminate		Indeterminate	Fragment		1	
1185	Mammalia	Indeterminate		Indeterminate	Fragment		1	
1186	Mammalia	Indeterminate		Indeterminate	Fragment		1	
1187	Mammalia	Indeterminate		Indeterminate	Fragment		1	
1188	Mammalia	Indeterminate		Indeterminate	Fragment		1	
1189	Mammalia	Indeterminate		Indeterminate	Fragment		1	
1190	Mammalia	Indeterminate		Indeterminate	Fragment		1	
1191	Mammalia	Indeterminate		Indeterminate	Fragment		1	
1192	Mammalia	Indeterminate		Indeterminate	Fragment		1	
1193	Mammalia	Indeterminate		Indeterminate	Fragment		1	
1194	Mammalia	Indeterminate		Indeterminate	Fragment		1	
1195	Mammalia	Indeterminate		Indeterminate	Fragment		1	
1196	Mammalia	Indeterminate		Indeterminate	Fragment		1	
1197	Mammalia	Indeterminate		Indeterminate	Fragment		1	
1198	Mammalia	Indeterminate		Indeterminate	Fragment		1	
1199	Mammalia	Indeterminate		Indeterminate	Fragment		1	
1200	Mammalia	Indeterminate		Indeterminate	Fragment		1	
1201	Mammalia	Indeterminate		Indeterminate	Fragment		1	
1202	Mammalia	Indeterminate		Indeterminate	Fragment		1	
1203	Mammalia	Indeterminate		Indeterminate	Fragment		1	
1204	Mammalia	Indeterminate		Indeterminate	Fragment		1	
1205	Mammalia	Indeterminate		Indeterminate	Fragment		1	
1206	Mammalia	Indeterminate		Indeterminate	Fragment		1	
1207	Mammalia	Indeterminate		Indeterminate	Fragment		1	
1208	Mammalia	Indeterminate		Indeterminate	Fragment		1	
1209	Mammalia	Indeterminate		Indeterminate	Fragment		1	

Supplemental Table S2. Catalogue of GDA Specimens: Identifiable Postcranial Specimens and Indeterminate Specimens

GA #	Class or Order	Family	Bovid Size Class	Element	Part or Portion	Side	Overall Size (cm)	Specimen Count
1210	Mammalia	Indeterminate		Indeterminate	Fragment		1	
1211	Mammalia	Indeterminate		Indeterminate	Fragment		1	
1212	Mammalia	Indeterminate		Indeterminate	Fragment		1	
1213	Mammalia	Indeterminate		Indeterminate	Fragment		1	
1214	Mammalia	Indeterminate		Indeterminate	Fragment		1	
1215	Mammalia	Indeterminate		Indeterminate	Fragment		1	
1216	Mammalia	Indeterminate		Indeterminate	Fragment		1	
1217	Mammalia	Indeterminate		Indeterminate	Fragment		1	
1218	Mammalia	Indeterminate		Indeterminate	Fragment		1	
1219	Mammalia	Indeterminate		Indeterminate	Fragment		1	
1220	Mammalia	Indeterminate		Indeterminate	Fragment		1	
1221	Mammalia	Indeterminate		Indeterminate	Fragment		1	
1222	Mammalia	Indeterminate		Indeterminate	Fragment		1	
1223	Mammalia	Indeterminate		Indeterminate	Fragment		1	
1224	Mammalia	Indeterminate		Indeterminate	Fragment		1	
1226	Mammalia	Indeterminate		Indeterminate	Fragment		1	
1227	Mammalia	Indeterminate		Indeterminate	Fragment		1	
1228	Mammalia	Indeterminate		Indeterminate	Fragment		1	
1230	Mammalia	Indeterminate		Indeterminate	Fragment		1	
1231	Mammalia	Indeterminate		Indeterminate	Fragment		1	
1232	Mammalia	Indeterminate		Indeterminate	Fragment		1	
1233	Mammalia	Indeterminate		Indeterminate	Fragment		1	
1234	Mammalia	Indeterminate		Indeterminate	Fragment		1	
1235	Mammalia	Indeterminate		Indeterminate	Fragment		1	
1237	Mammalia	Indeterminate		Indeterminate	Fragment		1	
1238	Mammalia	Indeterminate		Indeterminate	Fragment		1	
1239	Mammalia	Indeterminate		Indeterminate	Fragment		1	
1240	Mammalia	Indeterminate		Indeterminate	Fragment		1	
1241	Mammalia	Indeterminate		Indeterminate	Fragment		1	
1242	Mammalia	Indeterminate		Indeterminate	Fragment		1	
1243	Mammalia	Indeterminate		Indeterminate	Fragment		1	
1244	Mammalia	Indeterminate		Indeterminate	Fragment		1	
1245	Mammalia	Indeterminate		Indeterminate	Fragment		1	
1246	Mammalia	Indeterminate		Indeterminate	Fragment		1	
1249	Mammalia	Indeterminate		Cranial	Fragment		1	
1250	Mammalia	Indeterminate		Indeterminate	Fragment		1	
1251	Mammalia	Indeterminate		Indeterminate	Fragment		1	
1252	Mammalia	Indeterminate		Indeterminate	Fragment		1	
1253	Mammalia	Indeterminate		Indeterminate	Fragment		1	
1255	Mammalia	Indeterminate		Indeterminate	Fragment		1	
1256	Mammalia	Indeterminate		Indeterminate	Fragment		1	
1257	Mammalia	Indeterminate		Indeterminate	Fragment		1	
1260	Mammalia	Indeterminate		Indeterminate	Fragment		1	
1261	Mammalia	Indeterminate		Indeterminate	Fragment		1	
1262	Mammalia	Indeterminate		Indeterminate	Fragment		1	
1263	Mammalia	Indeterminate		Indeterminate	Fragment		1	
1264	Mammalia	Indeterminate		Indeterminate	Fragment		1	
1265	Mammalia	Indeterminate		Indeterminate	Fragment		1	
1266	Mammalia	Indeterminate		Indeterminate	Fragment		1	
1267	Mammalia	Indeterminate		Indeterminate	Fragment		1	
1268	Mammalia	Indeterminate		Indeterminate	Fragment		1	
1269	Mammalia	Indeterminate		Indeterminate	Fragment		1	
1270	Mammalia	Indeterminate		Indeterminate	Fragment		1	
1271	Mammalia	Indeterminate		Indeterminate	Fragment		1	
1273	Mammalia	Indeterminate		Indeterminate	Fragment		1	
1274	Mammalia	Indeterminate		Indeterminate	Fragment		1	
1275	Mammalia	Indeterminate		Indeterminate	Fragment		1	
1276	Mammalia	Indeterminate		Indeterminate	Fragment		1	
1277	Mammalia	Indeterminate		Indeterminate	Fragment		1	
1278	Mammalia	Indeterminate		Indeterminate	Fragment		1	
1279	Mammalia	Indeterminate		Indeterminate	Fragment		1	
1280	Mammalia	Indeterminate		Indeterminate	Fragment		1	
1281	Mammalia	Indeterminate		Indeterminate	Fragment		1	
1282	Mammalia	Indeterminate		Indeterminate	Fragment		1	
1283	Mammalia	Indeterminate		Indeterminate	Fragment		1	

Supplemental Table S2. Catalogue of GDA Specimens: Identifiable Postcranial Specimens and Indeterminate Specimens

GA #	Class or Order	Family	Bovid Size Class	Element	Part or Portion	Side	Overall Size (cm)	Specimen Count
1284	Mammalia	Indeterminate		Indeterminate	Fragment		1	
1285	Mammalia	Indeterminate		Indeterminate	Fragment		1	
1286	Mammalia	Indeterminate		Indeterminate	Fragment		1	
1287	Mammalia	Indeterminate		Indeterminate	Fragment		1	
1288	Mammalia	Indeterminate		Indeterminate	Fragment		1	
1289	Mammalia	Indeterminate		Indeterminate	Fragment		1	
1290	Mammalia	Indeterminate		Indeterminate	Fragment		1	
1291	Mammalia	Indeterminate		Indeterminate	Fragment		1	
1292	Mammalia	Indeterminate		Indeterminate	Fragment		1	
1293	Mammalia	Indeterminate		Indeterminate	Fragment		1	
1294	Mammalia	Indeterminate		Indeterminate	Fragment		1	
1296	Mammalia	Indeterminate		Indeterminate	Fragment		1	
1297	Mammalia	Indeterminate		Indeterminate	Fragment		1	
1298	Mammalia	Indeterminate		Indeterminate	Fragment		1	
1299	Mammalia	Indeterminate		Indeterminate	Fragment		1	
1300	Mammalia	Indeterminate		Indeterminate	Fragment		1	
1301	Mammalia	Indeterminate		Indeterminate	Fragment		1	
1302	Mammalia	Indeterminate		Indeterminate	Fragment		1	
1304	Mammalia	Indeterminate		Indeterminate	Fragment		1	
1305	Mammalia	Indeterminate		Indeterminate	Fragment		1	
1307	Mammalia	Indeterminate		Indeterminate	Fragment		6	
1308	Mammalia	Indeterminate		Indeterminate	Fragment		3	
1309	Mammalia	Indeterminate		Indeterminate	Fragment		3	
1310	Cetartiodactyla	Bovidae	II	Pelvis	Ischium	Left	7	
1311	Mammalia	Indeterminate		Indeterminate	Fragment		1	
1312	Mammalia	Indeterminate		Indeterminate	Fragment		1	
1314	Mammalia	Indeterminate		Indeterminate	Fragment		1	
1315	Mammalia	Indeterminate		Indeterminate	Fragment		1	
1316	Mammalia	Indeterminate		Indeterminate	Fragment		2	
1317	Mammalia	Indeterminate		Indeterminate	Fragment		1	
1318	Mammalia	Indeterminate		Indeterminate	Fragment		2	
1319	Mammalia	Indeterminate		Indeterminate	Fragment		2	
1320	Mammalia	Indeterminate		Indeterminate	Fragment		2	
1321	Mammalia	Indeterminate		Indeterminate	Fragment		2	
1322	Mammalia	Indeterminate		Indeterminate	Fragment		3	
1323	Mammalia	Indeterminate		Indeterminate	Fragment		3	
1324	Mammalia	Indeterminate		Indeterminate	Fragment		3	
1325	Mammalia	Indeterminate		Indeterminate	Fragment		3	
1326	Mammalia	Indeterminate		Indeterminate	Fragment		1	
1327	Mammalia	Indeterminate		Indeterminate	Fragment		1	
1328	Mammalia	Indeterminate		Indeterminate	Fragment		1	
1329	Mammalia	Indeterminate		Indeterminate	Fragment		1	
1330	Cetartiodactyla	Bovidae		Cranial	Enamel Fragment		1	
1331	Mammalia	Indeterminate		Cranial	Temporal		2	
1332	Mammalia	Indeterminate		Indeterminate	Fragment		1	
1333	Mammalia	Indeterminate		Indeterminate	Fragment		1	
1334	Mammalia	Indeterminate		Indeterminate	Fragment		1	
1335	Mammalia	Indeterminate		Indeterminate	Fragment		1	
1336	Mammalia	Indeterminate		Indeterminate	Fragment		1	
1337	Mammalia	Indeterminate		Indeterminate	Fragment		1	
1338	Mammalia	Indeterminate		Indeterminate	Fragment		1	
1339	Mammalia	Indeterminate		Indeterminate	Fragment		1	
1340	Mammalia	Indeterminate		Indeterminate	Fragment		1	
1341	Mammalia	Indeterminate		Indeterminate	Fragment		1	
1343	Mammalia	Indeterminate		Indeterminate	Fragment		0	N=10
1345	Mammalia	Indeterminate		Indeterminate	Fragment		1	
1347	Mammalia	Indeterminate		Cranial	Enamel Fragment		1	
1348	Mammalia	Indeterminate		Indeterminate	Fragment		1	
1350	Mammalia	Indeterminate		Indeterminate	Fragment		1	
1352	Cetartiodactyla	Bovidae	I	Vertebra	Lumbar		2	
1356	Mammalia	Indeterminate		Indeterminate	Fragment		3	
1357	Mammalia	Indeterminate		Indeterminate	Fragment		3	
1359	Mammalia	Indeterminate		Indeterminate	Fragment		8	
1361	Mammalia	Indeterminate		Indeterminate	Fragment		0	N=2
1362	Mammalia	Indeterminate		Indeterminate	Fragment		1	

Supplemental Table S2. Catalogue of GDA Specimens: Identifiable Postcranial Specimens and Indeterminate Specimens

GA #	Class or Order	Family	Bovid Size Class	Element	Part or Portion	Side	Overall Size (cm)	Specimen Count
1363	Mammalia	Indeterminate		Indeterminate	Fragment		1	
1364	Mammalia	Indeterminate		Indeterminate	Fragment		1	
1365	Mammalia	Indeterminate		Indeterminate	Fragment		1	
1366	Mammalia	Indeterminate		Rib	Proximal epiphysis		1	
1367	Mammalia	Indeterminate		Cranial	Enamel Fragment		1	
1368	Mammalia	Indeterminate		Cranial	Enamel Fragment		1	
1370	Cetartiodactyla	Bovidae		Cranial	Enamel Fragment		1	
1372	Mammalia	Indeterminate		Indeterminate	Fragment		2	
1373	Mammalia	Indeterminate		Indeterminate	Fragment		2	
1374	Mammalia	Indeterminate		Indeterminate	Fragment		2	
1375	Mammalia	Indeterminate		Indeterminate	Fragment		2	
1376	Mammalia	Indeterminate		Indeterminate	Fragment		2	
1377	Mammalia	Indeterminate		Indeterminate	Fragment		3	
1378	Cetartiodactyla	Bovidae	III	Cranial	Temporal		3	
1379	Mammalia	Indeterminate		Cranial	Enamel Fragment		4	
1381	Mammalia	Indeterminate		Cranial	Fragment		2	
1384	Mammalia	Indeterminate		Cranial	Enamel Fragment		1	
1385	Cetartiodactyla	Bovidae		Cranial	Enamel Fragment		1	
1386	Cetartiodactyla	Bovidae		Cranial	Enamel Fragment		2	
1387	Mammalia	Indeterminate		Indeterminate	Fragment		1	
1388	Mammalia	Indeterminate		Indeterminate	Fragment		1	
1389	Mammalia	Indeterminate		Indeterminate	Fragment		1	
1390	Mammalia	Indeterminate		Indeterminate	Fragment		1	
1391	Mammalia	Indeterminate		Vertebra	Fragment		1	
1393	Mammalia	Indeterminate		Indeterminate	Fragment		1	
1394	Mammalia	Indeterminate		Indeterminate	Fragment		1	
1395	Mammalia	Indeterminate		Indeterminate	Fragment		1	
1396	Mammalia	Indeterminate		Indeterminate	Fragment		1	
1397	Mammalia	Indeterminate		Indeterminate	Fragment		1	
1398	Mammalia	Indeterminate		Indeterminate	Fragment		1	
1399	Mammalia	Indeterminate		Indeterminate	Fragment		1	
1400	Mammalia	Indeterminate		Indeterminate	Fragment		2	
1401	Mammalia	Indeterminate		Indeterminate	Fragment		3	
1402	Cetartiodactyla	Bovidae		Cranial	Enamel Fragment		1	
1403	Cetartiodactyla	Bovidae		Cranial	Enamel Fragment		1	
1404	Cetartiodactyla	Bovidae		Cranial	Enamel Fragment		1	
1405	Cetartiodactyla	Bovidae		Cranial	Enamel Fragment		1	
1406	Cetartiodactyla	Bovidae		Cranial	Enamel Fragment		1	
1407	Cetartiodactyla	Bovidae		Cranial	Enamel Fragment		1	
1409	Cetartiodactyla	Bovidae		Cranial	Enamel Fragment		1	
1410	Mammalia	Indeterminate		Indeterminate	Fragment		1	
1414	Cetartiodactyla	Bovidae		Cranial	Enamel Fragment		2	
1422	Cetartiodactyla	Bovidae		Cranial	Enamel Fragment		1	
1423	Cetartiodactyla	Bovidae		Cranial	Enamel Fragment		1	
1424	Mammalia	Indeterminate		Indeterminate	Fragment		1	
1425	Cetartiodactyla	Bovidae		Cranial	Enamel Fragment		1	
1426	Mammalia	Indeterminate		Indeterminate	Fragment		1	
1427	Mammalia	Indeterminate		Cranial	Enamel Fragment		1	
1428	Mammalia	Indeterminate		Indeterminate	Fragment		1	
1429	Mammalia	Indeterminate		Scapula	Glenoid		4	
1430	Cetartiodactyla	Bovidae	III	Radius	Distal epiphysis	Left	5	
1431	Mammalia	Indeterminate		Indeterminate	Fragment		10	
1432	Cetartiodactyla	Bovidae	II	Metacarpal	Proximal epiphysis	Left	10	
1433	Mammalia	Indeterminate		Indeterminate	Fragment		1	
1434	Mammalia	Indeterminate		Indeterminate	Fragment		1	
1435	Mammalia	Indeterminate		Indeterminate	Fragment		1	
1436	Mammalia	Indeterminate		Indeterminate	Fragment		1	
1437	Mammalia	Indeterminate		Indeterminate	Fragment		1	
1438	Mammalia	Indeterminate		Indeterminate	Fragment		1	
1439	Mammalia	Indeterminate		Indeterminate	Fragment		1	
1440	Mammalia	Indeterminate		Indeterminate	Fragment		1	
1441	Mammalia	Indeterminate		Indeterminate	Fragment		1	
1442	Mammalia	Indeterminate		Indeterminate	Fragment		1	
1444	Mammalia	Indeterminate		Indeterminate	Fragment		1	
1445	Mammalia	Indeterminate		Indeterminate	Fragment		1	

Supplemental Table S2. Catalogue of GDA Specimens: Identifiable Postcranial Specimens and Indeterminate Specimens

GA #	Class or Order	Family	Bovid Size Class	Element	Part or Portion	Side	Overall Size (cm)	Specimen Count
1446	Mammalia	Indeterminate		Indeterminate	Fragment		1	
1447	Mammalia	Indeterminate		Indeterminate	Fragment		1	
1448	Cetartiodactyla	Bovidae		Cranial	Enamel Fragment		1	
1450	Mammalia	Indeterminate		Cranial	Enamel Fragment		1	
1451	Cetartiodactyla	Bovidae		Cranial	Enamel Fragment		1	
1452	Mammalia	Indeterminate		Cranial	Enamel Fragment		1	
1453	Cetartiodactyla	Bovidae		Cranial	Enamel Fragment		1	
1455	Mammalia	Indeterminate		Indeterminate	Fragment		0	N=42
1456	Mammalia	Indeterminate		Cranial	Enamel Fragment		1	
1457	Mammalia	Indeterminate		Indeterminate	Fragment		5	
1458	Cetartiodactyla	Bovidae	II	Vertebra	Atlas		5	
1459	Mammalia	Indeterminate		Cranial	Enamel Fragment		0	
1460	Mammalia	Indeterminate		Cranial	Enamel Fragment		1	
1462	Cetartiodactyla	Bovidae	II	Cranial	Maxillary molar		2	
1463	Mammalia	Indeterminate		Indeterminate	Fragment		0	N=3
1464	Mammalia	Indeterminate		Indeterminate	Fragment		1	
1466	Mammalia	Indeterminate		Cranial	Enamel Fragment		1	
1467	Mammalia	Indeterminate		Indeterminate	Fragment		1	
1468	Mammalia	Indeterminate		Indeterminate	Fragment		1	
1469	Mammalia	Indeterminate		Indeterminate	Fragment		1	
1470	Mammalia	Indeterminate		Indeterminate	Fragment		1	
1471	Mammalia	Indeterminate		Indeterminate	Fragment		1	
1472	Mammalia	Indeterminate		Indeterminate	Fragment		2	
1475	Mammalia	Indeterminate		Indeterminate	Fragment		1	
1477	Mammalia	Indeterminate		Indeterminate	Fragment		1	
1478	Mammalia	Indeterminate		Indeterminate	Fragment		2	
1480	Mammalia	Indeterminate		Indeterminate	Fragment		1	
1481	Mammalia	Indeterminate		Indeterminate	Fragment		2	
1485	Mammalia	Indeterminate		Indeterminate	Fragment		6	
1486	Mammalia	Indeterminate		Indeterminate	Fragment		7	
1491	Mammalia	Indeterminate		Indeterminate	Fragment		1	
1492	Mammalia	Indeterminate		Indeterminate	Fragment		1	
1493	Mammalia	Indeterminate		Indeterminate	Fragment		5	
1494	Mammalia	Indeterminate		Indeterminate	Fragment		0	
1496	Mammalia	Indeterminate		Vertebra	Fragment		1	
1498	Cetartiodactyla	Bovidae	III	Cranial	Maxillary premolar		2	
1500	Mammalia	Indeterminate		Indeterminate	Fragment		6	
1501	Mammalia	Indeterminate		Indeterminate	Fragment		0	N=3
1502	Mammalia	Indeterminate		Indeterminate	Fragment		1	
1503	Mammalia	Indeterminate		Indeterminate	Fragment		1	
1504	Mammalia	Indeterminate		Indeterminate	Fragment		1	
1505	Cetartiodactyla	Bovidae	II	Cranial	Enamel Fragment		1	
1506	Mammalia	Indeterminate		Indeterminate	Fragment		1	
1507	Mammalia	Indeterminate		Indeterminate	Fragment		2	
1508	Mammalia	Indeterminate		Indeterminate	Fragment		2	
1509	Mammalia	Indeterminate		Cranial	Enamel Fragment		2	
1510	Mammalia	Indeterminate		Indeterminate	Fragment		2	
1511	Mammalia	Indeterminate		Indeterminate	Fragment		3	
1512	Cetartiodactyla	Bovidae	II	Cranial	Enamel Fragment		3	
1513	Mammalia	Indeterminate		Indeterminate	Fragment		0	N=15
1514	Mammalia	Indeterminate		Cranial	Enamel Fragment		1	
1515	Mammalia	Indeterminate		Indeterminate	Fragment		1	
1516	Mammalia	Indeterminate		Indeterminate	Fragment		1	
1517	Mammalia	Indeterminate		Indeterminate	Fragment		1	
1518	Mammalia	Indeterminate		Indeterminate	Fragment		1	
1519	Mammalia	Indeterminate		Indeterminate	Fragment		1	
1520	Mammalia	Indeterminate		Indeterminate	Fragment		1	
1521	Mammalia	Indeterminate		Indeterminate	Fragment		1	
1522	Cetartiodactyla	Bovidae	II	Vertebra	Lumbar		1	
1523	Mammalia	Indeterminate		Indeterminate	Fragment		1	
1524	Cetartiodactyla	Bovidae	II	Vertebra	Lumbar		1	
1525	Mammalia	Indeterminate		Indeterminate	Fragment		1	
1526	Mammalia	Indeterminate		Indeterminate	Fragment		1	
1527	Mammalia	Indeterminate		Indeterminate	Fragment		1	
1528	Mammalia	Indeterminate		Indeterminate	Fragment		1	

Supplemental Table S2. Catalogue of GDA Specimens: Identifiable Postcranial Specimens and Indeterminate Specimens

GA #	Class or Order	Family	Bovid Size Class	Element	Part or Portion	Side	Overall Size (cm)	Specimen Count
1529	Cetartiodactyla	Bovidae	II	Cranial	Mandible		2	
1530	Cetartiodactyla	Bovidae	II	Metapodial	Distal epiphysis		2	
1531	Mammalia	Indeterminate		Indeterminate	Fragment		2	
1532	Mammalia	Indeterminate		Indeterminate	Fragment		2	
1533	Mammalia	Indeterminate		Indeterminate	Fragment		2	
1534	Mammalia	Indeterminate		Indeterminate	Fragment		2	
1535	Mammalia	Indeterminate		Indeterminate	Fragment		1	
1536	Mammalia	Indeterminate		Indeterminate	Fragment		2	
1537	Mammalia	Indeterminate		Cranial	Enamel Fragment		1	
1538	Mammalia	Indeterminate		Cranial	Enamel Fragment		1	
1539	Mammalia	Indeterminate		Cranial	Enamel Fragment		1	
1540	Cetartiodactyla	Bovidae		Cranial	Enamel Fragment		1	
1541	Cetartiodactyla	Bovidae		Cranial	Enamel Fragment		1	
1542	Mammalia	Indeterminate		Cranial	Enamel Fragment		1	
1543	Mammalia	Indeterminate		Cranial	Enamel Fragment		1	
1544	Mammalia	Indeterminate		Cranial	Enamel Fragment		1	
1545	Cetartiodactyla	Bovidae		Cranial	Enamel Fragment		1	
1546	Cetartiodactyla	Bovidae		Cranial	Enamel Fragment		1	
1547	Mammalia	Indeterminate		Cranial	Enamel Fragment		1	
1548	Mammalia	Indeterminate		Indeterminate	Fragment		1	
1549	Cetartiodactyla	Bovidae		Cranial	Enamel Fragment		1	
1550	Mammalia	Indeterminate		Cranial	Enamel Fragment		1	
1551	Cetartiodactyla	Bovidae		Cranial	Enamel Fragment		1	
1552	Mammalia	Indeterminate		Cranial	Enamel Fragment		1	
1553	Cetartiodactyla	Bovidae		Cranial	Enamel Fragment		1	
1554	Cetartiodactyla	Bovidae		Cranial	Enamel Fragment		1	
1555	Cetartiodactyla	Bovidae		Cranial	Enamel Fragment		1	
1556	Cetartiodactyla	Bovidae		Cranial	Enamel Fragment		1	
1557	Cetartiodactyla	Bovidae		Cranial	Enamel Fragment		2	
1558	Mammalia	Indeterminate		Cranial	Enamel Fragment		2	
1559	Cetartiodactyla	Bovidae		Cranial	Enamel Fragment		2	
1560	Cetartiodactyla	Bovidae		Cranial	Enamel Fragment		2	
1561	Mammalia	Indeterminate		Cranial	Enamel Fragment		1	
1562	Cetartiodactyla	Bovidae		Cranial	Enamel Fragment		1	
1563	Mammalia	Indeterminate		Cranial	Enamel Fragment		1	
1564	Mammalia	Indeterminate		Cranial	Enamel Fragment		1	
1565	Mammalia	Indeterminate		Cranial	Enamel Fragment		1	
1566	Cetartiodactyla	Bovidae		Cranial	Enamel Fragment		1	
1567	Mammalia	Indeterminate		Indeterminate	Fragment		1	
1570	Mammalia	Indeterminate		Indeterminate	Fragment		1	
1571	Mammalia	Indeterminate		Indeterminate	Fragment		1	
1572	Mammalia	Indeterminate		Indeterminate	Fragment		1	
1573	Mammalia	Indeterminate		Indeterminate	Fragment		1	
1574	Mammalia	Indeterminate		Indeterminate	Fragment		1	
1575	Mammalia	Indeterminate		Indeterminate	Fragment		1	
1576	Mammalia	Indeterminate		Indeterminate	Fragment		1	
1577	Mammalia	Indeterminate		Indeterminate	Fragment		1	
1578	Mammalia	Indeterminate		Indeterminate	Fragment		1	
1579	Mammalia	Indeterminate		Indeterminate	Fragment		1	
1580	Mammalia	Indeterminate		Indeterminate	Fragment		1	
1581	Mammalia	Indeterminate		Indeterminate	Fragment		1	
1582	Mammalia	Indeterminate		Indeterminate	Fragment		1	
1583	Mammalia	Indeterminate		Indeterminate	Fragment		1	
1584	Mammalia	Indeterminate		Indeterminate	Fragment		1	
1585	Cetartiodactyla	Bovidae		Cranial	Enamel Fragment		1	
1586	Mammalia	Indeterminate		Cranial	Enamel Fragment		1	
1587	Cetartiodactyla	Bovidae		Cranial	Enamel Fragment		1	
1588	Cetartiodactyla	Bovidae		Cranial	Enamel Fragment		1	
1589	Mammalia	Indeterminate		Cranial	Enamel Fragment		1	
1590	Mammalia	Indeterminate		Cranial	Enamel Fragment		1	
1591	Mammalia	Indeterminate		Cranial	Enamel Fragment		1	
1592	Mammalia	Indeterminate		Cranial	Enamel Fragment		1	
1593	Mammalia	Indeterminate		Cranial	Enamel Fragment		1	
1594	Mammalia	Indeterminate		Cranial	Enamel Fragment		1	
1595	Mammalia	Indeterminate		Cranial	Enamel Fragment		1	

Supplemental Table S2. Catalogue of GDA Specimens: Identifiable Postcranial Specimens and Indeterminate Specimens

GA #	Class or Order	Family	Bovid Size Class	Element	Part or Portion	Side	Overall Size (cm)	Specimen Count
1596	Cetartiodactyla	Bovidae		Cranial	Enamel Fragment		1	
1597	Mammalia	Indeterminate		Cranial	Enamel Fragment		1	
1598	Cetartiodactyla	Bovidae		Cranial	Enamel Fragment		1	
1599	Mammalia	Indeterminate		Cranial	Enamel Fragment		1	
1600	Mammalia	Indeterminate		Cranial	Enamel Fragment		1	
1601	Cetartiodactyla	Bovidae		Cranial	Enamel Fragment		1	
1602	Mammalia	Indeterminate		Cranial	Enamel Fragment		1	
1603	Cetartiodactyla	Bovidae		Cranial	Enamel Fragment		1	
1605	Mammalia	Indeterminate		Indeterminate	Fragment		1	
1606	Mammalia	Indeterminate		Indeterminate	Fragment		1	
1607	Mammalia	Indeterminate		Indeterminate	Fragment		1	
1608	Mammalia	Indeterminate		Indeterminate	Fragment		1	
1609	Mammalia	Indeterminate		Indeterminate	Fragment		1	
1610	Mammalia	Indeterminate		Indeterminate	Fragment		1	
1611	Mammalia	Indeterminate		Indeterminate	Fragment		1	
1612	Mammalia	Indeterminate		Indeterminate	Fragment		1	
1613	Mammalia	Indeterminate		Indeterminate	Fragment		1	
1614	Mammalia	Indeterminate		Indeterminate	Fragment		1	
1615	Mammalia	Indeterminate		Indeterminate	Fragment		1	
1616	Mammalia	Indeterminate		Indeterminate	Fragment		1	
1617	Mammalia	Indeterminate		Indeterminate	Fragment		2	
1618	Mammalia	Indeterminate		Indeterminate	Fragment		2	
1619	Mammalia	Indeterminate		Indeterminate	Fragment		1	
1620	Mammalia	Indeterminate		Indeterminate	Fragment		1	
1623	Mammalia	Indeterminate		Cranial	Fragment		3	
1624	Cetartiodactyla	Bovidae		Cranial	Enamel Fragment		1	N=2
1625	Mammalia	Indeterminate		Cranial	Fragment		1	
1626	Mammalia	Indeterminate		Indeterminate	Fragment		2	N=2
1627	Mammalia	Indeterminate		Cranial	Enamel Fragment		0	
1628	Mammalia	Indeterminate		Cranial	Fragment		3	
1629	Mammalia	Indeterminate		Indeterminate	Fragment		1	N=3
1630	Mammalia	Indeterminate		Cranial	Fragment		1	
1631	Mammalia	Indeterminate		Indeterminate	Fragment		2	N=4
1632	Mammalia	Indeterminate		Indeterminate	Fragment		1	N=33
1633	Mammalia	Indeterminate		Indeterminate	Fragment		0	N=26
1634	Mammalia	Indeterminate		Indeterminate	Fragment		2	
1635	Mammalia	Indeterminate		Indeterminate	Fragment		2	
1636	Mammalia	Indeterminate		Indeterminate	Fragment		2	
1637	Mammalia	Indeterminate		Indeterminate	Fragment		2	
1638	Mammalia	Indeterminate		Indeterminate	Fragment		2	
1639	Mammalia	Indeterminate		Indeterminate	Fragment		2	
1640	Mammalia	Indeterminate		Indeterminate	Fragment		2	N=17
1641	Mammalia	Indeterminate		Indeterminate	Fragment		1	N=38
1643	Mammalia	Indeterminate		Indeterminate	Fragment		2	N=17
1644	Mammalia	Indeterminate		Indeterminate	Fragment		1	N=127
1645	Mammalia	Indeterminate		Indeterminate	Fragment		0	N=163
1646	Mammalia	Indeterminate		Indeterminate	Fragment		3	N=2
1647	Mammalia	Indeterminate		Indeterminate	Fragment		3	
1648	Mammalia	Indeterminate		Indeterminate	Fragment		4	
1649	Mammalia	Indeterminate		Indeterminate	Fragment		4	
1651	Mammalia	Indeterminate		Indeterminate	Fragment		1	N=9
1652	Cetartiodactyla	Bovidae		Cranial	Mandibular premolar	Left	1	
1653	Mammalia	Indeterminate		Cranial	Enamel Fragment		1	
1654	Mammalia	Indeterminate		Indeterminate	Fragment		2	
1655	Mammalia	Indeterminate		Indeterminate	Fragment		1	N=14
1657	Mammalia	Indeterminate		Indeterminate	Fragment		2	N=6
1658	Mammalia	Indeterminate		Indeterminate	Fragment		1	N=41
1659	Mammalia	Indeterminate		Indeterminate	Fragment		0	N=70
1660	Mammalia	Indeterminate		Indeterminate	Fragment		3	N=2
1661	Cetartiodactyla	Bovidae		Cranial	Mandible	Right	4	
1662	Mammalia	Indeterminate		Indeterminate	Fragment		4	
1663	Mammalia	Indeterminate		Indeterminate	Fragment		2	N=2
1664	Mammalia	Indeterminate		Indeterminate	Fragment		1	N=14
1665	Mammalia	Indeterminate		Cranial	Enamel Fragment		1	

Supplemental Table S2. Catalogue of GDA Specimens: Identifiable Postcranial Specimens and Indeterminate Specimens

GA #	Class or Order	Family	Bovid Size Class	Element	Part or Portion	Side	Overall Size (cm)	Specimen Count
1666	Cetartiodactyla	Bovidae		Cranial	Enamel Fragment		2	N=3
1667	Mammalia	Indeterminate		Cranial	Enamel Fragment		1	N=5
1668	Mammalia	Indeterminate		Indeterminate	Fragment		1	
1669	Mammalia	Indeterminate		Indeterminate	Fragment		2	N=6
1670	Mammalia	Indeterminate		Indeterminate	Fragment		1	
1671	Mammalia	Indeterminate		Cranial	Enamel Fragment		0	
1672	Mammalia	Indeterminate		Indeterminate	Fragment		1	
1673	Mammalia	Indeterminate		Indeterminate	Fragment		1	N=35
1674	Mammalia	Indeterminate		Indeterminate	Fragment		0	N=60
1675	Mammalia	Indeterminate		Indeterminate	Fragment		3	
1676	Mammalia	Indeterminate		Indeterminate	Fragment		4	
1678	Cetartiodactyla	Bovidae		Cranial	Enamel Fragment		1	
1679	Cetartiodactyla	Bovidae		Cranial	Enamel Fragment		1	
1680	Mammalia	Indeterminate		Indeterminate	Fragment		1	
1681	Cetartiodactyla	Bovidae		Cranial	Enamel Fragment		1	
1682	Mammalia	Indeterminate		Indeterminate	Fragment		2	
1683	Mammalia	Indeterminate		Indeterminate	Fragment		2	N=5
1684	Mammalia	Indeterminate		Indeterminate	Fragment		1	N=12
1685	Mammalia	Indeterminate		Cranial	Enamel Fragment		0	N=3
1686	Mammalia	Indeterminate		Indeterminate	Fragment		2	N=7
1687	Mammalia	Indeterminate		Indeterminate	Fragment		1	N=52
1688	Mammalia	Indeterminate		Indeterminate	Fragment		0	N=90
1689	Mammalia	Indeterminate		Indeterminate	Fragment		2	
1690	Mammalia	Indeterminate		Indeterminate	Fragment		2	N=2
1691	Mammalia	Indeterminate		Indeterminate	Fragment		1	N=20
1692	Mammalia	Indeterminate		Cranial	Enamel Fragment		0	N=8
1693	Mammalia	Indeterminate		Cranial	Enamel Fragment		1	
1694	Mammalia	Indeterminate		Rib	Proximal epiphysis		0	
1696	Mammalia	Indeterminate		Indeterminate	Fragment		2	N=8
1697	Mammalia	Indeterminate		Indeterminate	Fragment		1	N=7
1699	Mammalia	Indeterminate		Indeterminate	Fragment		2	N=9
1700	Mammalia	Indeterminate		Indeterminate	Fragment		1	N=49
1701	Mammalia	Indeterminate		Indeterminate	Fragment		0	N=39
1702	Mammalia	Indeterminate		Indeterminate	Fragment		3	N=3
1703	Mammalia	Indeterminate		Indeterminate	Fragment		4	
1704	Mammalia	Indeterminate		Indeterminate	Fragment		3	N=4
1706	Cetartiodactyla	Bovidae		Cranial	Enamel Fragment		1	N=36
1707	Mammalia	Indeterminate		Cranial	Enamel Fragment		0	
1708	Mammalia	Indeterminate		Cranial	Enamel Fragment		1	
1709	Mammalia	Indeterminate		Cranial	Enamel Fragment		1	
1710	Mammalia	Indeterminate		Indeterminate	Fragment		1	N=2
1711	Mammalia	Indeterminate		Cranial	Enamel Fragment		0	N=2
1712	Mammalia	Indeterminate		Indeterminate	Fragment		1	N=8
1713	Mammalia	Indeterminate		Indeterminate	Fragment		0	N=22
1714	Mammalia	Indeterminate		Indeterminate	Fragment		1	
1715	Mammalia	Indeterminate		Indeterminate	Fragment		1	N=3
1716	Mammalia	Indeterminate		Indeterminate	Fragment		0	
1717	Mammalia	Indeterminate		Indeterminate	Fragment		2	N=3
1719	Mammalia	Indeterminate		Cranial	Enamel Fragment		1	
1720	Mammalia	Indeterminate		Indeterminate	Fragment		1	N=3
1721	Cetartiodactyla	Bovidae		Cranial	Enamel Fragment		1	
1722	Mammalia	Indeterminate		Indeterminate	Fragment		1	N=32
1723	Mammalia	Indeterminate		Indeterminate	Fragment		0	N=103
1724	Mammalia	Indeterminate		Indeterminate	Fragment		1	
1725	Mammalia	Indeterminate		Indeterminate	Fragment		1	N=5
1727	Mammalia	Indeterminate		Indeterminate	Fragment		2	
1728	Mammalia	Indeterminate		Cranial	Enamel Fragment		1	
1729	Mammalia	Indeterminate		Indeterminate	Fragment		0	N=23
1732	Mammalia	Indeterminate		Cranial	Enamel Fragment		1	
1733	Mammalia	Indeterminate		Indeterminate	Fragment		2	
1734	Mammalia	Indeterminate		Indeterminate	Fragment		1	N=4
1735	Cetartiodactyla	Bovidae	III	Cranial	Mandibular second incisor	Right	2	
1736	Mammalia	Indeterminate		Indeterminate	Fragment		3	
1737	Mammalia	Indeterminate		Indeterminate	Fragment		2	N=3

Supplemental Table S2. Catalogue of GDA Specimens: Identifiable Postcranial Specimens and Indeterminate Specimens

GA #	Class or Order	Family	Bovid Size Class	Element	Part or Portion	Side	Overall Size (cm)	Specimen Count
1738	Mammalia	Indeterminate		Indeterminate	Fragment		1	N=3
1739	Mammalia	Indeterminate		Indeterminate	Fragment		1	N=4
1740	Mammalia	Indeterminate		Rib	Proximal epiphysis		1	
1742	Mammalia	Indeterminate		Carpal or Tarsal	Fragment		1	
1744	Mammalia	Indeterminate		Indeterminate	Fragment		2	N=6
1745	Mammalia	Indeterminate		Indeterminate	Fragment		1	N=3
1746	Mammalia	Indeterminate		Cranial	Enamel Fragment		0	N=4
1747	Mammalia	Indeterminate		Indeterminate	Fragment		2	N=4
1748	Mammalia	Indeterminate		Indeterminate	Fragment		1	N=30
1749	Mammalia	Indeterminate		Indeterminate	Fragment		0	N=19
1750	Mammalia	Indeterminate		Indeterminate	Fragment		3	
1751	Mammalia	Indeterminate		Indeterminate	Fragment		4	
1753	Mammalia	Indeterminate		Indeterminate	Fragment		1	N=28
1754	Cetartiodactyla	Bovidae	I	Proximal Phalanx	Proximal epiphysis		1	
1755	Mammalia	Indeterminate		Indeterminate	Fragment		2	N=5
1757	Cetartiodactyla	Bovidae	II	Astragalus	Proximal epiphysis		1	
1758	Mammalia	Indeterminate		Indeterminate	Fragment		2	N=15
1759	Mammalia	Indeterminate		Indeterminate	Fragment		1	N=42
1760	Mammalia	Indeterminate		Indeterminate	Fragment		3	
1761	Mammalia	Indeterminate		Indeterminate	Fragment		2	N=15
1762	Mammalia	Indeterminate		Indeterminate	Fragment		1	N=101
1763	Mammalia	Indeterminate		Indeterminate	Fragment		0	N=119
1764	Mammalia	Indeterminate		Indeterminate	Fragment		3	N=6
1765	Mammalia	Indeterminate		Indeterminate	Fragment		1	N=6
1766	Cetartiodactyla	Bovidae		Cranial	Mandibular third molar		1	
1767	Mammalia	Indeterminate		Indeterminate	Fragment		1	N=2
1768	Mammalia	Indeterminate		Indeterminate	Fragment		0	N=96
1769	Mammalia	Indeterminate		Indeterminate	Fragment		1	N=9
1770	Cetartiodactyla	Bovidae		Cranial	Enamel Fragment		0	
1772	Mammalia	Indeterminate		Indeterminate	Fragment		1	
1773	Mammalia	Indeterminate		Indeterminate	Fragment		2	
1775	Cetartiodactyla	Bovidae	I	Pelvis	Pubis	Right	2	
1776	Mammalia	Indeterminate		Indeterminate	Fragment		1	
1777	Mammalia	Indeterminate		Indeterminate	Fragment		0	N=23
1778	Mammalia	Indeterminate		Cranial	Enamel Fragment		1	N=14
1779	Mammalia	Indeterminate		Indeterminate	Fragment		1	N=2
1781	Mammalia	Indeterminate		Indeterminate	Fragment		2	N=4
1782	Cetartiodactyla	Bovidae		Cranial	Enamel Fragment		2	N=5
1783	Mammalia	Indeterminate		Indeterminate	Fragment		4	
1784	Mammalia	Indeterminate		Indeterminate	Fragment		2	N=12
1785	Mammalia	Indeterminate		Indeterminate	Fragment		1	N=11
1786	Mammalia	Indeterminate		Indeterminate	Fragment		1	
1787	Mammalia	Indeterminate		Indeterminate	Fragment		2	N=20
1788	Mammalia	Indeterminate		Indeterminate	Fragment		1	N=84
1789	Mammalia	Indeterminate		Indeterminate	Fragment		0	N=34
1790	Mammalia	Indeterminate		Indeterminate	Fragment		3	N=5
1791	Mammalia	Indeterminate		Indeterminate	Fragment		4	
1792	Mammalia	Indeterminate		Indeterminate	Fragment		2	
1793	Mammalia	Indeterminate		Indeterminate	Fragment		1	
1794	Mammalia	Indeterminate		Cranial	Enamel Fragment		0	
1795	Mammalia	Indeterminate		Cranial	Enamel Fragment		0	
1796	Mammalia	Indeterminate		Indeterminate	Fragment		2	
1797	Mammalia	Indeterminate		Indeterminate	Fragment		1	N=5
1798	Mammalia	Indeterminate		Indeterminate	Fragment		0	N=8
1799	Mammalia	Indeterminate		Indeterminate	Fragment		3	
1800	Mammalia	Indeterminate		Indeterminate	Fragment		2	
1801	Mammalia	Indeterminate		Indeterminate	Fragment		2	
1802	Mammalia	Indeterminate		Indeterminate	Fragment		1	
1803	Cetartiodactyla	Bovidae	II	Metapodial	Distal epiphysis		1	
1804	Mammalia	Indeterminate		Indeterminate	Fragment		1	N=2
1805	Mammalia	Indeterminate		Rib	Diaphysis		1	
1806	Mammalia	Indeterminate		Indeterminate	Fragment		0	N=45
1807	Mammalia	Indeterminate		Tibia	Proximal epiphysis	Left	0	
1808	Mammalia	Indeterminate		Indeterminate	Fragment		2	N=2

Supplemental Table S2. Catalogue of GDA Specimens: Identifiable Postcranial Specimens and Indeterminate Specimens

GA #	Class or Order	Family	Bovid Size Class	Element	Part or Portion	Side	Overall Size (cm)	Specimen Count
1810	Mammalia	Indeterminate		Rib	Diaphysis		2	
1812	Cetartiodactyla	Bovidae	II	Cranial	Mandible	Right	3	
1813	Mammalia	Indeterminate		Vertebra	Fragment		1	
1814	Mammalia	Indeterminate		Indeterminate	Fragment		2	N=2
1815	Mammalia	Indeterminate		Indeterminate	Fragment		1	N=6
1817	Mammalia	Indeterminate		Indeterminate	Fragment		1	N=33
1818	Mammalia	Indeterminate		Indeterminate	Fragment		0	N=35
1820	Mammalia	Indeterminate		Vertebra	Fragment		1	
1821	Mammalia	Indeterminate		Indeterminate	Fragment		7	
1822	Cetartiodactyla	Bovidae	I	Metacarpal	Proximal epiphysis	Left	1	
1823	Mammalia	Indeterminate		Indeterminate	Fragment		2	
1824	Mammalia	Indeterminate		Indeterminate	Fragment		3	
1825	Mammalia	Indeterminate		Indeterminate	Fragment		2	N=2
1826	Mammalia	Indeterminate		Indeterminate	Fragment		1	N=2
1827	Mammalia	Indeterminate		Indeterminate	Fragment		3	
1828	Mammalia	Indeterminate		Indeterminate	Fragment		1	N=5
1829	Mammalia	Indeterminate		Indeterminate	Fragment		0	N=13
1830	Cetartiodactyla	Bovidae		Cranial	Enamel Fragment		1	
1831	Mammalia	Indeterminate		Indeterminate	Fragment		1	N=3
1832	Mammalia	Indeterminate		Indeterminate	Fragment		2	
1833	Mammalia	Indeterminate		Indeterminate	Fragment		3	
1835	Mammalia	Indeterminate		Indeterminate	Fragment		1	N=9
1836	Mammalia	Indeterminate		Indeterminate	Fragment		0	N=5
1837	Mammalia	Indeterminate		Indeterminate	Fragment		1	
1838	Cetartiodactyla	Bovidae		Cranial	Enamel Fragment		1	
1839	Mammalia	Indeterminate		Cranial	Enamel Fragment		2	
1840	Mammalia	Indeterminate		Indeterminate	Fragment		1	N=4
1841	Cetartiodactyla	Bovidae		Cranial	Enamel Fragment		1	
1842	Mammalia	Indeterminate		Indeterminate	Fragment		0	N=11
1843	Mammalia	Indeterminate		Indeterminate	Fragment		1	
1844	Mammalia	Indeterminate		Indeterminate	Fragment		1	N=2
1845	Cetartiodactyla	Bovidae	II	Metatarsal	Distal epiphysis		4	
1846	Mammalia	Indeterminate		Indeterminate	Fragment		2	N=3
1847	Mammalia	Indeterminate		Indeterminate	Fragment		1	N=2
1848	Cetartiodactyla	Bovidae	II	Metatarsal	Diaphysis		3	
1849	Mammalia	Indeterminate		Indeterminate	Fragment		2	N=3
1850	Mammalia	Indeterminate		Indeterminate	Fragment		1	N=8
1851	Mammalia	Indeterminate		Indeterminate	Fragment		0	N=44
1852	Mammalia	Indeterminate		Indeterminate	Fragment		2	
1853	Mammalia	Indeterminate		Indeterminate	Fragment		1	
1854	Mammalia	Indeterminate		Cranial	Enamel Fragment		1	
1855	Mammalia	Indeterminate		Cranial	Enamel Fragment		0	
1856	Mammalia	Indeterminate		Indeterminate	Fragment		2	N=5
1857	Mammalia	Indeterminate		Indeterminate	Fragment		1	N=4
1859	Mammalia	Indeterminate		Indeterminate	Fragment		1	N=18
1860	Mammalia	Indeterminate		Indeterminate	Fragment		0	N=44
1861	Mammalia	Indeterminate		Indeterminate	Fragment		1	N=6
1862	Mammalia	Indeterminate		Vertebra	Enamel Fragment		2	
1863	Cetartiodactyla	Bovidae	II	Vertebra	Cervical		2	
1864	Cetartiodactyla	Bovidae	II	Tarsal	Second	Left	1	
1865	Mammalia	Indeterminate		Cranial	Fragment		4	
1866	Mammalia	Indeterminate		Vertebra	Fragment		3	
1867	Mammalia	Indeterminate		Indeterminate	Fragment		1	N=2
1868	Mammalia	Indeterminate		Indeterminate	Fragment		0	N=16
1869	Mammalia	Indeterminate		Indeterminate	Fragment		2	N=2
1870	Mammalia	Indeterminate		Indeterminate	Fragment		1	N=14
1871	Cetartiodactyla	Bovidae	I	Vertebra	Lumbar		2	
1872	Cetartiodactyla	Bovidae	I	Cranial	Temporal	Right	2	
1873	Cetartiodactyla	Bovidae	I	Vertebra	Lumbar		1	
1874	Mammalia	Indeterminate		Rib	Diaphysis		1	
1875	Mammalia	Indeterminate		Indeterminate	Fragment		1	N=11
1876	Mammalia	Indeterminate		Vertebra	Caudal		1	

Supplemental Table S2. Catalogue of GDA Specimens: Identifiable Postcranial Specimens and Indeterminate Specimens

GA #	Class or Order	Family	Bovid Size Class	Element	Part or Portion	Side	Overall Size (cm)	Specimen Count
1877	Mammalia	Indeterminate		Indeterminate	Fragment		2	N=4
1878	Mammalia	Indeterminate		Indeterminate	Fragment		1	N=46
1879	Mammalia	Indeterminate		Indeterminate	Fragment		0	N=33
1880	Mammalia	Indeterminate		Indeterminate	Fragment		3	
1881	Mammalia	Indeterminate		Cranial	Enamel Fragment		0	N=2
1882	Mammalia	Indeterminate		Indeterminate	Fragment		1	N=5
1883	Mammalia	Indeterminate		Indeterminate	Fragment		2	
1884	Mammalia	Indeterminate		Cranial	Enamel Fragment		1	
1885	Mammalia	Indeterminate		Indeterminate	Fragment		1	
1886	Mammalia	Indeterminate		Indeterminate	Fragment		0	N=45
1887	Mammalia	Indeterminate		Indeterminate	Fragment		2	N=3
1888	Mammalia	Indeterminate		Indeterminate	Fragment		1	N=5
1889	Mammalia	Indeterminate		Cranial	Fragment		2	
1889	Mammalia	Indeterminate		Indeterminate	Fragment		1	
1890	Cetartiodactyla	Bovidae		Cranial	Enamel Fragment		1	
1891	Mammalia	Indeterminate		Rib	Diaphysis		2	
1892	Mammalia	Indeterminate		Indeterminate	Fragment		1	N=8
1893	Mammalia	Indeterminate		Cranial	Fragment		2	
1893	Mammalia	Indeterminate		Indeterminate	Fragment		1	
1894	Mammalia	Indeterminate		Indeterminate	Fragment		1	N=21
1895	Mammalia	Indeterminate		Indeterminate	Fragment		0	N=51
1896	Mammalia	Indeterminate		Cranial	Fragment		2	
1896	Mammalia	Indeterminate		Cranial	Enamel Fragment		1	
1897	Mammalia	Indeterminate		Indeterminate	Fragment		1	N=5
1898	Cetartiodactyla	Bovidae	II	Vertebra	Lumbar		4	
1899	Cetartiodactyla	Bovidae	II	Tibia	Diaphysis	Left	4	
1900	Mammalia	Indeterminate		Indeterminate	Fragment		1	N=2
1901	Mammalia	Indeterminate		Indeterminate	Fragment		0	N=3
1902	Mammalia	Indeterminate		Indeterminate	Fragment		2	N=2
1903	Mammalia	Indeterminate		Indeterminate	Fragment		1	N=4
1904	Mammalia	Indeterminate		Indeterminate	Fragment		1	N=2
1905	Mammalia	Indeterminate		Indeterminate	Fragment		1	
1906	Cetartiodactyla	Bovidae	II	Vertebra	Enamel Fragment		4	
1907	Mammalia	Indeterminate		Indeterminate	Fragment		3	
1909	Mammalia	Indeterminate		Indeterminate	Fragment		0	N=45
1910	Cetartiodactyla	Bovidae	II	Intermediate Phalanx	Proximal epiphysis		1	
1911	Mammalia	Indeterminate		Indeterminate	Fragment		4	
1912	Mammalia	Indeterminate		Indeterminate	Fragment		4	N=3
1913	Mammalia	Indeterminate		Indeterminate	Fragment		4	N=9
1914	Mammalia	Indeterminate		Indeterminate	Fragment		4	N=7
1916	Mammalia	Indeterminate		Indeterminate	Fragment		3	N=4
1917	Cetartiodactyla	Bovidae	II	Metapodial	Diaphysis		3	
1918	Mammalia	Indeterminate		Indeterminate	Fragment		3	N=27
1919	Mammalia	Indeterminate		Indeterminate	Fragment		3	N=72
1920	Mammalia	Indeterminate		Indeterminate	Fragment		3	N=44
1921	Mammalia	Indeterminate		Indeterminate	Fragment		1	N=239
1922	Mammalia	Indeterminate		Indeterminate	Fragment		1	N=25
1923	Mammalia	Indeterminate		Indeterminate	Fragment		1	N=66
1924	Mammalia	Indeterminate		Cranial	Fragment		1	
1925	Mammalia	Indeterminate		Indeterminate	Fragment		1	N=4194
1927	Mammalia	Indeterminate		Indeterminate	Fragment		2	N=593
1928	Mammalia	Indeterminate		Indeterminate	Fragment		2	N=52
1929	Mammalia	Indeterminate		Indeterminate	Fragment		2	N=3
1930	Cetartiodactyla	Bovidae	I	Pelvis	Acetabulum		3	
1931	Mammalia	Indeterminate		Indeterminate	Fragment		2	N=186
1932	Mammalia	Indeterminate		Indeterminate	Fragment		2	
1933	Cetartiodactyla	Bovidae		Cranial	Enamel Fragment		5	
1934	Cetartiodactyla	Bovidae		Cranial	Enamel Fragment		4	N=5
1935	Cetartiodactyla	Bovidae		Cranial	Enamel Fragment		0	N=100
1936	Cetartiodactyla	Bovidae		Cranial	Enamel Fragment		3	N=33
1937	Cetartiodactyla	Bovidae		Cranial	Enamel Fragment		2	N=149
1938	Cetartiodactyla	Bovidae		Cranial	Enamel Fragment		1	N=636
1940	Mammalia	Indeterminate		Cranial	Enamel Fragment		1	N=4

Supplemental Table S2. Catalogue of GDA Specimens: Identifiable Postcranial Specimens and Indeterminate Specimens

GA #	Class or Order	Family	Bovid Size Class	Element	Part or Portion	Side	Overall Size (cm)	Specimen Count
1941	Cetartiodactyla	Bovidae		Cranial	Enamel Fragment		0	
1944	Cetartiodactyla	Bovidae		Cranial	Enamel Fragment		3	N=2
1945	Mammalia	Indeterminate		Cranial	Enamel Fragment		2	N=3
1946	Mammalia	Indeterminate		Cranial	Enamel Fragment		1	N=20
1947	Mammalia	Indeterminate		Cranial	Enamel Fragment		0	N=105
1948	Cetartiodactyla	Bovidae		Cranial	Enamel Fragment		2	N=3
1949	Mammalia	Indeterminate		Cranial	Enamel Fragment		1	
1950	Mammalia	Indeterminate		Cranial	Enamel Fragment		0	N=156
1952	Cetartiodactyla	Bovidae		Cranial	Enamel Fragment		2	
1953	Cetartiodactyla	Bovidae		Cranial	Enamel Fragment		1	N=7
1955	Cetartiodactyla	Bovidae		Cranial	Enamel Fragment		1	N=8
1956	Cetartiodactyla	Bovidae		Cranial	Enamel Fragment		0	
1958	Mammalia	Indeterminate		Cranial	Enamel Fragment		0	N=2
1961	Mammalia	Indeterminate		Vertebra	Lumbar		0	
1962	Cetartiodactyla	Bovidae		Cranial	Enamel Fragment		1	N=2
1963	Cetartiodactyla	Bovidae		Cranial	Enamel Fragment		2	
1965	Mammalia	Indeterminate		Cranial	Enamel Fragment		0	N=6
1969	Cetartiodactyla	Bovidae		Cranial	Enamel Fragment		1	
1970	Cetartiodactyla	Bovidae		Cranial	Enamel Fragment		0	N=2
1971	Mammalia	Indeterminate		Cranial	Enamel Fragment		0	
1977	Cetartiodactyla	Bovidae		Cranial	Enamel Fragment		2	N=3
1978	Cetartiodactyla	Bovidae		Cranial	Enamel Fragment		1	N=6
1980	Cetartiodactyla	Bovidae		Cranial	Enamel Fragment		2	N=5
1981	Cetartiodactyla	Bovidae		Cranial	Enamel Fragment		1	N=8
1982	Mammalia	Indeterminate		Cranial	Enamel Fragment		0	N=82
1983	Mammalia	Indeterminate		Cranial	Enamel Fragment		1	N=12
1986	Cetartiodactyla	Bovidae		Cranial	Enamel Fragment		1	
1988	Mammalia	Indeterminate		Cranial	Enamel Fragment		2	
1991	Mammalia	Indeterminate		Indeterminate	Fragment		1	N=29
1992	Mammalia	Indeterminate		Indeterminate	Fragment		1	N=5
1994	Mammalia	Indeterminate		Indeterminate	Fragment		1	N=10
1995	Mammalia	Indeterminate		Indeterminate	Fragment		0	N=210
1997	Mammalia	Indeterminate		Indeterminate	Fragment		1	
1998	Mammalia	Indeterminate		Cranial	Enamel Fragment		1	
1999	Mammalia	Indeterminate		Indeterminate	Fragment		2	N=4
2000	Mammalia	Indeterminate		Indeterminate	Fragment		3	
2001	Cetartiodactyla	Bovidae		Cranial	Enamel Fragment		1	N=5
2002	Mammalia	Indeterminate		Cranial	Enamel Fragment		0	N=14
2005	Mammalia	Indeterminate		Indeterminate	Fragment		4	
2006	Cetartiodactyla	Bovidae	II	Metapodial	Distal epiphysis		4	
2007	Cetartiodactyla	Bovidae	II	Astragalus	Complete	Left	4	
2008	Cetartiodactyla	Bovidae	II	Pelvis	Ischium	Left	3	
2009	Mammalia	Indeterminate		Indeterminate	Fragment		3	
2010	Cetartiodactyla	Bovidae	II	Calcaneus	Distal epiphysis	Right	2	
2011	Mammalia	Indeterminate		Indeterminate	Fragment		2	
2012	Cetartiodactyla	Bovidae	II	Cranial	Temporal	Right	2	
2013	Mammalia	Indeterminate		Cranial	Maxilla		2	
2014	Cetartiodactyla	Bovidae	I	Pelvis	Pubis	Right	2	
2016	Mammalia	Indeterminate		Indeterminate	Fragment		1	
2017	Cetartiodactyla	Bovidae	II	Intermediate Phalanx	Proximal epiphysis		2	
2018	Mammalia	Indeterminate		Indeterminate	Fragment		3	
2019	Mammalia	Indeterminate		Indeterminate	Fragment		3	
2020	Mammalia	Indeterminate		Indeterminate	Fragment		3	
2021	Cetartiodactyla	Bovidae	I	Metacarpal	Distal epiphysis		3	
2022	Mammalia	Indeterminate		Indeterminate	Fragment		3	
2023	Mammalia	Indeterminate		Indeterminate	Fragment		3	
2024	Cetartiodactyla	Bovidae	II	Tibia	Proximal epiphysis		3	
2025	Mammalia	Indeterminate		Indeterminate	Fragment		4	
2027	Mammalia	Indeterminate		Indeterminate	Fragment		6	
2028	Cetartiodactyla	Bovidae	II	Cranial	Mandible	Left	6	
2030	Mammalia	Indeterminate		Indeterminate	Fragment		3	
2037	Mammalia	Indeterminate		Indeterminate	Fragment		3	
2040	Cetartiodactyla	Bovidae		Cranial	Enamel Fragment		1	N=26
2041	Mammalia	Indeterminate		Indeterminate	Fragment		1	N=59
2042	Cetartiodactyla	Bovidae		Cranial	Enamel Fragment		2	N=16

Supplemental Table S2. Catalogue of GDA Specimens: Identifiable Postcranial Specimens and Indeterminate Specimens

GA #	Class or Order	Family	Bovid Size Class	Element	Part or Portion	Side	Overall Size (cm)	Specimen Count
2043	Cetartiodactyla	Bovidae		Cranial	Enamel Fragment		3	N=7
2044	Mammalia	Indeterminate		Cranial	Enamel Fragment		0	N=4
2045	Mammalia	Indeterminate		Cranial	Enamel Fragment		0	
2046	Mammalia	Indeterminate		Indeterminate	Fragment		1	N=11
2047	Mammalia	Indeterminate		Indeterminate	Fragment		2	N=2
2050	Cetartiodactyla	Bovidae	II	Metatarsal	Diaphysis		3	
2051	Mammalia	Indeterminate		Indeterminate	Fragment		2	
2052	Mammalia	Indeterminate		Indeterminate	Fragment		2	
2053	Mammalia	Indeterminate		Indeterminate	Fragment		2	
2054	Mammalia	Indeterminate		Indeterminate	Fragment		2	
2055	Mammalia	Indeterminate		Indeterminate	Fragment		2	
2056	Mammalia	Indeterminate		Indeterminate	Fragment		2	
2057	Mammalia	Indeterminate		Indeterminate	Fragment		2	
2058	Mammalia	Indeterminate		Indeterminate	Fragment		2	
2059	Mammalia	Indeterminate		Indeterminate	Fragment		2	
2060	Mammalia	Indeterminate		Indeterminate	Fragment		2	
2061	Mammalia	Indeterminate		Indeterminate	Fragment		2	
2062	Mammalia	Indeterminate		Indeterminate	Fragment		2	
2063	Mammalia	Indeterminate		Indeterminate	Fragment		2	
2064	Mammalia	Indeterminate		Indeterminate	Fragment		2	
2065	Mammalia	Indeterminate		Indeterminate	Fragment		2	
2066	Mammalia	Indeterminate		Indeterminate	Fragment		2	
2067	Mammalia	Indeterminate		Indeterminate	Fragment		2	
2068	Mammalia	Indeterminate		Indeterminate	Fragment		2	
2069	Mammalia	Indeterminate		Indeterminate	Fragment		2	
2070	Mammalia	Indeterminate		Indeterminate	Fragment		2	
2071	Mammalia	Indeterminate		Indeterminate	Fragment		3	
2072	Mammalia	Indeterminate		Indeterminate	Fragment		3	
2073	Mammalia	Indeterminate		Indeterminate	Fragment		3	
2074	Mammalia	Indeterminate		Indeterminate	Fragment		3	
2075	Mammalia	Indeterminate		Indeterminate	Fragment		3	
2076	Mammalia	Indeterminate		Cranial	Enamel Fragment		0	N=4
2077	Mammalia	Indeterminate		Cranial	Enamel Fragment		0	
2078	Mammalia	Indeterminate		Indeterminate	Fragment		2	N=28
2079	Cetartiodactyla	Bovidae		Cranial	Enamel Fragment		0	N=3
2080	Mammalia	Indeterminate		Cranial	Enamel Fragment		1	N=12
2081	Cetartiodactyla	Bovidae		Cranial	Enamel Fragment		1	N=14
2082	Cetartiodactyla	Bovidae		Cranial	Enamel Fragment		0	N=2
2083	Cetartiodactyla	Bovidae		Cranial	Enamel Fragment		1	N=8
2084	Cetartiodactyla	Bovidae		Cranial	Enamel Fragment		0	N=2
2085	Cetartiodactyla	Bovidae		Cranial	Enamel Fragment		1	
2086	Cetartiodactyla	Bovidae		Cranial	Enamel Fragment		1	N=12
2087	Cetartiodactyla	Bovidae		Cranial	Enamel Fragment		1	N=18
2088	Cetartiodactyla	Bovidae		Cranial	Enamel Fragment		0	N=2
2089	Mammalia	Indeterminate		Indeterminate	Fragment		1	N=6
2090	Mammalia	Indeterminate		Cranial	Enamel Fragment		0	
2091	Cetartiodactyla	Bovidae		Cranial	Enamel Fragment		0	N=2
2092	Mammalia	Indeterminate		Cranial	Enamel Fragment		1	
2093	Cetartiodactyla	Bovidae		Cranial	Enamel Fragment		2	N=2
2094	Cetartiodactyla	Bovidae		Cranial	Enamel Fragment		0	N=2
2095	Cetartiodactyla	Bovidae		Cranial	Enamel Fragment		1	N=4
2097	Mammalia	Indeterminate		Cranial	Enamel Fragment		1	
2098	Mammalia	Indeterminate		Cranial	Enamel Fragment		1	
2099	Mammalia	Indeterminate		Cranial	Enamel Fragment		1	
2101	Mammalia	Indeterminate		Cranial	Enamel Fragment		2	
2103	Cetartiodactyla	Bovidae		Cranial	Enamel Fragment		1	
2104	Cetartiodactyla	Bovidae		Cranial	Enamel Fragment		1	
2106	Cetartiodactyla	Bovidae		Cranial	Enamel Fragment		1	
2107	Cetartiodactyla	Bovidae	II	Cranial	Maxillary molar		1	
2108	Cetartiodactyla	Bovidae		Cranial	Molar		1	
2109	Cetartiodactyla	Bovidae		Cranial	Enamel Fragment		1	
2110	Mammalia	Indeterminate		Cranial	Enamel Fragment		1	
2111	Cetartiodactyla	Bovidae		Cranial	Enamel Fragment		1	
2112	Cetartiodactyla	Bovidae		Cranial	Enamel Fragment		1	
2113	Mammalia	Indeterminate		Cranial	Enamel Fragment		1	

Supplemental Table S2. Catalogue of GDA Specimens: Identifiable Postcranial Specimens and Indeterminate Specimens

GA #	Class or Order	Family	Bovid Size Class	Element	Part or Portion	Side	Overall Size (cm)	Specimen Count
2114	Mammalia	Indeterminate		Cranial	Enamel Fragment		1	
2116	Mammalia	Indeterminate		Cranial	Enamel Fragment		1	
2117	Cetartiodactyla	Bovidae	III	Cranial	Mandibular premolar	Right	1	
2118	Cetartiodactyla	Bovidae		Cranial	Mandibular second premolar	Right	1	
2120	Mammalia	Indeterminate		Cranial	Enamel Fragment		1	
2122	Cetartiodactyla	Bovidae	II	Cranial	Maxillary molar		3	
2124	Mammalia	Indeterminate		Cranial	Enamel Fragment		1	
2125	Cetartiodactyla	Bovidae		Cranial	Enamel Fragment		2	
2127	Mammalia	Indeterminate		Cranial	Enamel Fragment		1	
2128	Cetartiodactyla	Bovidae	II	Cranial	Mandibular first incisor	Right	1	
2130	Cetartiodactyla	Bovidae		Cranial	Enamel Fragment		1	
2131	Cetartiodactyla	Bovidae		Cranial	Enamel Fragment		1	
2133	Mammalia	Indeterminate		Cranial	Enamel Fragment		1	
2133	Mammalia	Indeterminate		Cranial	Enamel Fragment		1	
2134	Mammalia	Indeterminate		Cranial	Enamel Fragment		1	
2135	Cetartiodactyla	Bovidae		Cranial	Enamel Fragment		1	
2136	Mammalia	Indeterminate		Cranial	Enamel Fragment		1	
2137	Mammalia	Indeterminate		Cranial	Enamel Fragment		1	
2138	Cetartiodactyla	Bovidae		Cranial	Enamel Fragment		1	
2139	Cetartiodactyla	Bovidae		Cranial	Enamel Fragment		1	
2142	Cetartiodactyla	Bovidae		Cranial	Enamel Fragment		1	
2143	Cetartiodactyla	Bovidae		Cranial	Enamel Fragment		1	
2144	Mammalia	Indeterminate		Cranial	Fragment		2	
2145	Mammalia	Indeterminate		Cranial	Enamel Fragment		2	
2146	Cetartiodactyla	Bovidae		Cranial	Enamel Fragment		2	
2147	Mammalia	Indeterminate		Cranial	Enamel Fragment		2	
2148	Cetartiodactyla	Bovidae		Cranial	Enamel Fragment		2	
2149	Cetartiodactyla	Bovidae		Cranial	Mandibular third molar		2	
2150	Mammalia	Indeterminate		Cranial	Enamel Fragment		2	
2151	Cetartiodactyla	Bovidae		Cranial	Mandibular third molar		2	
2152	Mammalia	Indeterminate		Cranial	Enamel Fragment		2	
2153	Mammalia	Indeterminate		Cranial	Enamel Fragment		2	
2154	Cetartiodactyla	Bovidae		Cranial	Enamel Fragment		2	
2155	Cetartiodactyla	Bovidae		Cranial	Enamel Fragment		2	
2157	Mammalia	Indeterminate		Cranial	Enamel Fragment		1	
2158	Cetartiodactyla	Bovidae		Cranial	Enamel Fragment		2	
2159	Mammalia	Indeterminate		Indeterminate	Fragment		2	
2162	Cetartiodactyla	Bovidae	II	Humerus	Diaphysis	Left	6	
2163	Mammalia	Indeterminate		Cranial	Fragment		2	
2164	Mammalia	Indeterminate		Indeterminate	Fragment		3	
2166	Mammalia	Indeterminate		Cranial	Enamel Fragment		2	
2167	Mammalia	Indeterminate		Indeterminate	Fragment		2	
2171	Mammalia	Indeterminate		Indeterminate	Fragment		5	
2172	Mammalia	Indeterminate		Indeterminate	Fragment		2	
2178	Mammalia	Indeterminate		Humerus	Proximal epiphysis		4	
2179	Mammalia	Indeterminate		Cranial	Temporal	Right	4	
2180	Mammalia	Indeterminate		Indeterminate	Fragment		2	
2182	Mammalia	Indeterminate		Indeterminate	Fragment		2	
2182	Mammalia	Indeterminate		Indeterminate	Fragment		2	
2183	Mammalia	Indeterminate		Indeterminate	Fragment		3	
2184	Mammalia	Indeterminate		Indeterminate	Fragment		2	
2187	Mammalia	Indeterminate		Cranial	Fragment		2	
2188	Mammalia	Indeterminate		Indeterminate	Fragment		2	
2190	Mammalia	Indeterminate		Cranial	Fragment		1	
2190	Mammalia	Indeterminate		Indeterminate	Fragment		4	
2191	Mammalia	Indeterminate		Cranial	Fragment		2	
2192	Mammalia	Indeterminate		Indeterminate	Fragment		1	
2193	Mammalia	Indeterminate		Indeterminate	Fragment		1	
2194	Cetartiodactyla	Bovidae	II	Cranial	Mandible	Right	2	
2195	Mammalia	Indeterminate		Indeterminate	Fragment		2	
2196	Mammalia	Indeterminate		Cranial	Enamel Fragment		1	
2196	Mammalia	Indeterminate		Indeterminate	Fragment		2	
2197	Mammalia	Indeterminate		Indeterminate	Fragment		2	
2199	Mammalia	Indeterminate		Indeterminate	Fragment		1	
2200	Mammalia	Indeterminate		Cranial	Enamel Fragment		1	

Supplemental Table S2. Catalogue of GDA Specimens: Identifiable Postcranial Specimens and Indeterminate Specimens

GA #	Class or Order	Family	Bovid Size Class	Element	Part or Portion	Side	Overall Size (cm)	Specimen Count
2201	Mammalia	Indeterminate		Cranial	Fragment		1	
2202	Mammalia	Indeterminate		Indeterminate	Fragment		2	
2203	Mammalia	Indeterminate		Indeterminate	Fragment		2	
2204	Mammalia	Indeterminate		Indeterminate	Fragment		2	
2205	Mammalia	Indeterminate		Indeterminate	Fragment		2	
2206	Mammalia	Indeterminate		Indeterminate	Fragment		2	
2207	Mammalia	Indeterminate		Indeterminate	Fragment		1	
2209	Mammalia	Indeterminate		Indeterminate	Fragment		1	
2210	Mammalia	Indeterminate		Indeterminate	Fragment		1	
2211	Mammalia	Indeterminate		Indeterminate	Fragment		1	
2212	Mammalia	Indeterminate		Indeterminate	Fragment		1	
2213	Mammalia	Indeterminate		Indeterminate	Fragment		1	
2225	Cetartiodactyla	Bovidae	II	Cranial	Mandibular first incisor	Left	1	
